# Identification of PANoptosis-related subtypes, construction of a prognosis signature, and tumor microenvironment landscape of hepatocellular carcinoma using bioinformatic analysis and experimental verification

**DOI:** 10.3389/fimmu.2024.1323199

**Published:** 2024-04-29

**Authors:** Guoqing Ouyang, Qiuyun Li, Yangnian Wei, Wenbin Dai, Haojian Deng, Youli Liu, Jiaguang Li, Mingjuan Li, Shunwen Luo, Shuang Li, Yunying Liang, Guandong Pan, Jianqing Yang, Tao Gan

**Affiliations:** ^1^ Department of General Surgery, Liuzhou People’s Hospital Affiliated to Guangxi Medical University, Liuzhou, Guangxi, China; ^2^ Guangxi Key Laboratory of Early Prevention and Treatment for Regional High Frequency Tumor, Guangxi Medical University, Nanning, Guangxi, China; ^3^ Key Laboratory of Early Prevention and Treatment for Regional High Frequency Tumor, Guangxi Medical University, Ministry of Education, Nanning, Guangxi, China; ^4^ Liuzhou Hepatobiliary and Pancreatic Diseases Precision Diagnosis Research Center of Engineering Technology, Liuzhou People’s Hospital Affiliated to Guangxi Medical University, Liuzhou, Guangxi, China; ^5^ Department of Hepatobiliary Surgery, Ruikang Hospital, Guangxi University of Chinese Medicine, Nanning, Guangxi, China; ^6^ Department of Pathology, Liuzhou People’s Hospital Affiliated to Guangxi Medical University, Liuzhou, Guangxi, China; ^7^ Department of Emergency Medical, Liuzhou People’s Hospital Affiliated to Guangxi Medical University, Liuzhou, Guangxi, China; ^8^ Key Specialty Department of Emergency Medicine in Guangxi, Liuzhou People’s Hospital Affiliated to Guangxi Medical University, Liuzhou, Guangxi, China

**Keywords:** PANoptosis, hepatocellular carcinoma, tumor microenvironment, prognosis signature, drugs susceptibility

## Abstract

**Background:**

Hepatocellular carcinoma (HCC) is one of the most lethal malignancies worldwide. PANoptosis is a recently unveiled programmed cell death pathway, Nonetheless, the precise implications of PANoptosis within the context of HCC remain incompletely elucidated.

**Methods:**

We conducted a comprehensive bioinformatics analysis to evaluate both the expression and mutation patterns of PANoptosis-related genes (PRGs). We categorized HCC into two clusters and identified differentially expressed PANoptosis-related genes (DEPRGs). Next, a PANoptosis risk model was constructed using LASSO and multivariate Cox regression analyses. The relationship between PRGs, risk genes, the risk model, and the immune microenvironment was studies. In addition, drug sensitivity between high- and low-risk groups was examined. The expression profiles of these four risk genes were elucidate by qRT-PCR or immunohistochemical (IHC). Furthermore, the effect of CTSC knock down on HCC cell behavior was verified using *in vitro* experiments.

**Results:**

We constructed a prognostic signature of four DEPRGs (CTSC, CDCA8, G6PD, and CXCL9). Receiver operating characteristic curve analyses underscored the superior prognostic capacity of this signature in assessing the outcomes of HCC patients. Subsequently, patients were stratified based on their risk scores, which revealed that the low-risk group had better prognosis than those in the high-risk group. High-risk group displayed a lower Stromal Score, Immune Score, ESTIMATE score, and higher cancer stem cell content, tumor mutation burden (TMB) values. Furthermore, a correlation was noted between the risk model and the sensitivity to 56 chemotherapeutic agents, as well as immunotherapy efficacy, in patient with. These findings provide valuable guidance for personalized clinical treatment strategies. The qRT−PCR analysis revealed that upregulated expression of CTSC, CDCA8, and G6PD, whereas downregulated expression of CXCL9 in HCC compared with adjacent tumor tissue and normal liver cell lines. The knockdown of CTSC significantly reduced both HCC cell proliferation and migration.

**Conclusion:**

Our study underscores the promise of PANoptosis-based molecular clustering and prognostic signatures in predicting patient survival and discerning the intricacies of the tumor microenvironment within the context of HCC. These insights hold the potential to advance our comprehension of the therapeutic contribution of PANoptosis plays in HCC and pave the way for generating more efficacious treatment strategies.

## Introduction

Liver cancer ranking as the seventh most commonly diagnosed malignancy and the second leading cause of cancer-related mortality, is a significant global health concern. In 2020, 906,677 new cases and 830,180 deaths attributed to liver cancer were reported (1). The burden of liver cancer is steadily increasing, with the number of estimated incident projected to exceed one million by 2025 (2). The majority of liver cancer cases are hepatocellular carcinoma (HCC), accounting for 90% of live cancer (2). Current mainstay curative management options for HCC include surgical resection, radiofrequency ablation, and liver transplantation. However, a significant number of patients are diagnosed at an advanced stage, limiting the curative treatment options to transarterial chemoembolization (TACE), tyrosine kinase inhibitors (TKI), and immune checkpoint inhibitors (3). The prognosis for HCC remains poor, with an overall 5-year survival rate of only 18% (4). Therefore, it is essential to uncover the genomic characteristics of HCC and develop reliable and effective models for developing reliable and effective models to predict HCC prognosis and assess therapeutic responses, thereby enabling individualized and precise treatments.

Programmed cell death (PCD), including apoptosis, pyroptosis, and necroptosis has been implicated in the pathophysiology of HCC (5). Although these PCD pathways were traditionally considered independent, mounting evidence suggests intricate crosstalk among apoptosis, pyroptosis, and necroptosis (6). Thus an additional PCD pathway known as PANoptosis has recently emerged (7). It is a newly recognized form of inflammatory programmed cell death, which underscores the coordination and crosstalk between pyroptosis, apoptosis, and necroptosis (6, 7). During PANoptosis, these three pathways are collectively activated, forming the PANoptosome complex, which exhibits characteristics not explained by any individual death pathway (6, 8, 9). Although numerous studies have identified the roles of pyroptosis, apoptosis, and necroptosis in HCC (10–12), the relationship between HCC and PANoptosis, as well as its impact on anticancer immunity, remains unclear. Therefore, understanding the characteristics of PANoptosis may provide vital insight into the mechanisms underlying HCC tumorigenesis and facilitate the development of promising immunotherapy strategies for HCC.

In this study, we comprehensively integrated the expression profiles of 486 HCC patients to assess the PANoptosis-related molecular patterns into mechanisms contributing to HCC tumorigenesis and facilitate the development of promising immunotherapy strategies for HCC. A novel PANoptosis risk scoring system was developed to predict the prognosis of HCC patients and characterize the TME phenotype. Finally, we validated the expression levels of the four genes in our signature using quantitative polymerase chain reaction (qPCR) in both human samples and cells.

## Materials and methods

### HCC dataset and preprocessing

**Figure 1:**
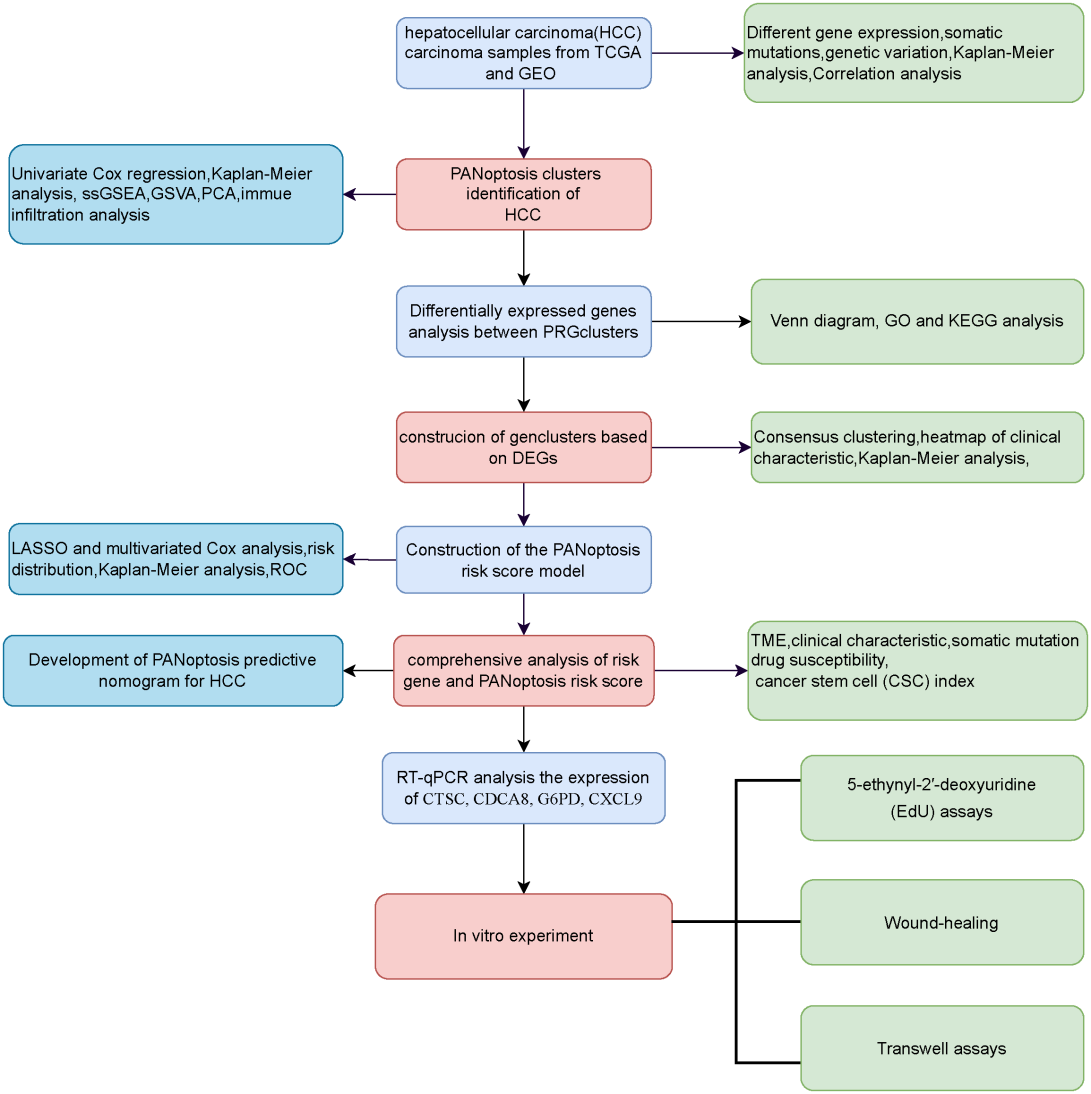
Flowchart of the present study.

The RAN-sequencing and corresponding clinical data of 371 HCC cases and 50 healthy cases were download from the TCGA database (https://portal.gdc.cancer.gov/) (13). The HCC gene expression profiles and clinical characteristics of GSE76427 (n=115) were enrolled from the GEO database (https://www.ncbi.nlm.nih.gov/geo/) (14). Gene symbols were converted from probes based on the GPL10558 platform annotation file. The patients with HCC whose survival information was unavailable excluded from the analysis. The data of TCGA and GEO databases were merged using the “sva” R package (15) to remove the batch effects. A total of 29 PANoptosis-related genes (PRGs) were enrolled from previous studies (6, 8, 16). The data of copy number variation (CNV) was downloaded from UCSC Xena (https://xenabrowser.net). The flowchart of this study is shown in **Figure 1**.

### Differential expression gene and consensus clustering analysis of CRGs

Wilcoxon rank-sum test was used to analysis the differential PRGs expression level between HCC patients and healthy samples using “limma” package (17). DEGs were selected with the threshold of p-value<0.05. We applied consensus clustering algorithms to categorize HCC patients into distinct molecular subtypes based on the expression of PRGs. This analysis was performed using the “ConsensusClusterPlus” (18) R package, and 1000 repetitions were conducted to ensure robustness. We next determined determine the optimal number of subtypes, we utilized a Cumulative Distribution Function (CDF) and evaluated the CDF Delta area. Additionally, Principal Component Analysis (PCA) was performed to confirm the differentiation of transcriptome profiles among the identified subgroups using the “ggplot2” R packages (19).

### Gene set variation analysis and functional enrichment analysis

We used the “GSVA” (20) R package to perform the GSVA analysis to detect biological functions distinguishing different PANoptosis subtypes. The gene sets of KEGG gene set “c2.cp.kegg.symbols.gmt” download from the MSigDB database (https://www.gsea-msigdb.org/gsea/index.jsp), was employed to conducted the GSVA analysis (21). The “clusterProfiler” (22) R package was used to performed the Kyoto Encyclopedia of Genes and Genomes (KEGG) and gene ontology (GO) analysis. The pathways exhibiting a p< 0.05, logFC > 0.5 were deemed statistically significant.

### Construction of PANoptosis risk model

A total of 485 HCC patients were randomly classified into testing and training group with a ratio of 1:1. Afterward, we identified 153 differentially expressed genes (DEGs) through performed three pairwise comparisons between the three PANoptosis subtypes, each time with a Log2 (fold change)> 0.585 and an adjusted P-value<0.05. The DEGs between three PANoptosis subtypes was intersected with each other to create PANoptosis gene signature.

Subsequently, we conducted univariate Cox regression analysis and identify 93 DEGs which significant associations with HCC prognosis to estimate significant genes. To mitigate overfitting, we employed LASSO Cox regression analysis (23). The best-performing gene was selected through multivariate Cox regression analysis, and a PANoptosis risk model was established using the formula: $\text{PANoptosis score}=\sum_{i=1}^{n}\text{exp}(\text{Xi})\times \text{coef}(\text{Xi})$ , where exp (Xi) represents the expression level and coef(Xi) represents the coefficient. Patients were stratified into high- and low-risk groups based on the median risk score. Time-dependent receiver operating characteristic (ROC) analysis was conducted to assess the sensitivity and specificity of the risk signature. A bootstrap method employing 1,000 resamplings was employed to generate the test set.

### Assessment of tumor microenvironment

The CIBERSORT algorithm (24) was utilized to estimate the fractions of 22 immune cell phenotypes in each HCC patients, with the sum of the proportion of all calculated immune cell phenotypes in each sample being equal to 1. Samples with a p-value of< 0.05 were deemed statistically significant. Utilizing CIBERSORT results, correlation analysis was conducted between risk genes and 22 phenotypes of immune cells using the “limma” and “ggplot2” packages. The R “ESTIMATE” package (25) was used to calculated the immune scores, stromal scores, and ESTIMATE scores for each HCC sample (26). Subsequently, we conducted Wilcoxon tests to analyze the differences in these scores between the two risk groups. For a more detailed assessment of immune cell infiltration, we applied the single-sample gene set enrichment analysis (ssGSEA) based on “gsva” package (27).

### Survival analysis of HCC

Afterward, we identified “survminer” and “survival” packages to generate Kaplan–Meier survival plots and assess the significance of differences using log-rank tests. The HCC patients were stratified into different subtypes, including PANoptosisCluster subtype, geneCluster subtype, PANoptosisScore subtype, and TMB subtype.

### Development of nomograms

We developed nomograms to quantitatively predict of 1-, 3-, and 5-year overall survival (OS) by incorporating both clinical characteristics and risk score based on HCC patients’ survival. Within the nomogram scoring system, individual variables such as gender, age, stage, and PRG Risk score were paired with corresponding scores. The cumulative score for each sample was derived by summing the assigned scores across all variables. The prognostic performance of the nomograms was assessed by calibration plots, which evaluated the concordance between predicted and actual values. The “rms” R package was used to construct the nomograms and conducting the calibration plot analysis.

### Assessment of mutation, and cancer stem cell index

We next analyzed the mutations in HCC patients from both high- and low-risk groups, using the R package “maftools” (28) to generate mutation annotations. Initially, the total count of nonsynonymous mutations in each sample was computed. Subsequently, genes with high mutation frequencies were discerned utilizing a threshold of mutation frequency>5. The discrepancies in mutation frequency between different groups were then evaluated. Additionally, we examined the correlation between the cancer stem cell index and risk scores using the Spearman method (29).

### Drug sensitivity analysis

We next assessed the drug sensitivity of every patient utilizing 198 drugs obtained from the genomics of drug sensitivity in the cancer v2 (GDSC) database (https://www.cancerrxgene.org/) and calculated their sensitivity by the “oncoPredict” R package (30). Statistical significance was determined at p< 0.05.

### Cell culture and siRNA transfection

The HCCLM3, huh7, sun449, HepG2, HCCLM3, MHCC97-H of HCC cell lines and THLE-3 of normal liver cells, were cultured in Dulbecco’s Modified Eagle Medium (DMEM) supplemented with penicillin G (100 mg/mL), streptomycin (100 mg/mL), and 10% fetal bovine serum (FBS; Gibco; USA). These cultures were incubated at 37°C in a 5% CO_2_ atmosphere. Logarithmically growing cells were selected to conduct experiments. SUN449 was employed for siRNA transfection. For transfection, we employed Lipofectamine 3000 Transfection Reagent (Invitrogen, Waltham, Massachusetts, USA) in conjunction with 5 nmol of the specified siRNA fragments and a negative control si-NC (GenePharma, Shanghai, China) into approximately 4×105 SUN449 cells following the manufacturer’s instructions. Si-NC (GenePharma) was used as a negative control. To assess transfection efficiency, quantitative reverse transcription-polymerase chain reaction (qRT-PCR) were employed. The sequences listed in **Supplementary Table 1**.

### RNA extraction and quantitative real-time PCR

Total RNA was isolated from human samples of adjacent tumor tissues, HCC, normal liver cells (THLE-3), and HCC cells (huh7, sun449, HepG2, HCCLM3, MHCC97-H) using the Trizol reagent (Thermo Fisher Scientific, United States) following the manufacturer’s instructions. Reverse transcription was carried out using the PrimeScriptTM RT reagent Kit (Takara, Japan). Next, qRT-PCR was performed on an FX Connect system (Bio-Rad, United States) using the SYBR ^®^ Green Supermix (Bio-Rad, United States) to measure the expression levels of hub genes. β-actin was used as an internal control for normalization. RT-qPCR was measured 3 times, with 3 biological replicates each time. The relative expression levels of the target genes were calculated using the 2^-ΔΔCT^ method. 15 patient’s HCC tissue and adjacent tissue were used for qRT-PCR and a Student’s t-test used to analyzed. Primer sequences used in the qRT-PCR assays are provided in **Supplementary Table 1**.

### Human specimens and immunohistochemical staining

Human specimens were collected from 15 patients diagnosed with HCC at LiuZhou People’s Hospital affiliated to Guangxi medical university. The study protocol was reviewed and approved by the Medical Ethics Committee of LiuZhou People’s Hhospital affiliated to Guangxi medical university. All patients provided written informed consent in accordance with the Declaration of Helsinki. Human tissue specimens were fixed with 4% paraformaldehyde, embedded in paraffin, and sectioned into 5 mm slices by a slicer. The specimens were dewaxed with xylene, following which the tissue sections were rehydrated using a graded series of ethanol solutions for antigen retrieval. The tissue sections were repaired with a sodium citrate repair solution (from Fuzhou Maixin Biotechnology Development Co., Ltd.), followed by allowing the sections to cool. Subsequently, an adequate amount of endogenous peroxidase blocker (supplied by Beijing Zhongshan Jinqiao Biotechnology Co., Ltd.) was added, and the sections were incubated at room temperature for 10 minutes. Afterward, the sections were washed three times with PBS, with each wash lasting 3 minutes. The sections were then blocked with 10% goat serum and incubated overnight at 4°C with anti-CTSC antibody (1:100) (Santa Cruze, U.S.A, cat#:sc-74590). Following three washes with PBS, the sections were incubated with a secondary antibody for 30 mins at 25°C, followed by development and then developed with DAB for 10 mins. Next, the sections were counterstained with hematoxylin for 2 mins to visualize nuclei. 15 patient’s tumor and adjacent tumor tissue were used to qRT-PCR and immunohistochemical staining. Student’s t-test or Wilcoxon test was used to compared the two group and p< 0.05 was regarded as significance.

### Wound-healing and Transwell assays

We next studied the invasion capability and cell migration capacity by conducting Transwell assays and wound healing assays, respectively. For the Transwell assays, Prior to the experiment, the experimental cells underwent a period of serum starvation and were cultured in serum-free medium for 24 hours. Following this, the cells were digested, the digestion process was halted, and then centrifuged at 1500 rpm for 3 minutes. After aspirating the supernatant, the cells were washed with PBS and counted. Subsequently, the cells were resuspended in serum-free medium. The cell density was adjusted to 1 × 10^4 cells/mL, and 500 μL of culture medium containing 15% FBS was added to each well of a 24-well plate. Next, 200 μL of cell suspension was added to the chamber, which was carefully placed into the well of a 24-well plate containing complete culture medium to prevent the formation of air bubbles. The cells were then incubated in a cell culture incubator for 48 hours. Following incubation, the cells on the chamber were aspirated, and any remaining cells were gently wiped off using a PBS-dried cotton swab. The cells were fixed with a 10% methanol solution for 30 seconds, stained with 0.1% crystal violet for 20 minutes, and washed with tap water until the background was clear. Finally, 3-5 fields of view were randomly selected under an upright microscope, and the number of cells passing through the membrane was counted. Photomicrographs were captured and counted using Image J software. For the wound-healing assay, transfected SUN449 cells were seeded in a 6-well plate. When the cells reached 90% confluence, a 200 μL pipette tip was used to create a vertical scratch in the cell monolayer. Washed 3 times with PBS, removed the scratched cells, and added serum-free medium. The cells were then cultured for an additional 24 hours in a 37°C 5% CO2 incubator. Images were acquired and documented initially at the 0-hour time point, with additional imaging performed at 24 hours. Kruskal-Wallis test was used to analysis the Wound-healing and transwell assays results.

### 5-ethynyl-2′-deoxyuridine assays

SUN449 cells were seeded into a 12-well plate. After overnight incubation and return to a normal state, the cells were transfected with siRNA. Subsequently, an equal volume of 2X EdU working solution (20 μM) (Beyotime, China), preheated to 37°C, was added to the 12 wells plate, and the cells were incubated for 2 hours. Once EdU labeling was completed, the culture medium was removed, and the cells were fixed with 500 μl of fixative solution for 15 minutes. Following fixation, the cells were washed three times with 500 μl washing solution per well, with each wash lasting 3-5 minutes. After washing, permeabilization solution (500 μl per well) was added and incubated for 15 minutes, followed by 2 additional washes with 1 ml washing solution per well. Subsequently, 200 μl of Click reaction solution (Beyotime, China) was added, and the cells were incubated in the dark for 30 minutes. After removing the Click reaction solution, the cells were washed three times with washing solution for 3-5 minutes each. Nuclear staining was performed using Hoechst 33342, with protection from light, for 10 minutes. Following staining, the cells were washed three times with washing solution for 3-5 minutes each. Finally, fluorescence detection could be carried out.

### Immunohistochemistry

Paraffin sections of HCC tissue from 15 patients and adjacent tumor tissue from the same group were subjected to immunostaining using antibodies against CTSC. Prior to staining, a dual endogenous enzyme blocker (MXB Biotechnologies, China) was applied for 30 minutes. The primary antibodies were left to incubate overnight at 4°C. Following thorough washing, the tissues were treated with the appropriate secondary antibodies and incubated at 37°C for 30 minutes. Next, an appropriate amount of DAB solution was applied for staining, followed by counterstaining with hematoxylin. To complete the process, a layer of neutral gum was used to cover the slides and the slides were sealed. The staining results was observed using an inverted microscope.

### Statistical analysis

All statistical analyses were performed using the R software version 4.2.2 and GraphPad Prism 9. Continuous data are presented as means ± standard deviations. Student’s t-test was used for normally distributed data in two-group comparisons, whereas the Wilcoxon test was used for non-normally distributed data. For comparisons involving more than two groups, the Kruskal-Wallis test was used. Statistical significance was defined as p< 0.05. ALL experiment was repeated three times independently.

## Results

### Differential expression and genetic variation of PRGs in HCC

**Figure 2:**
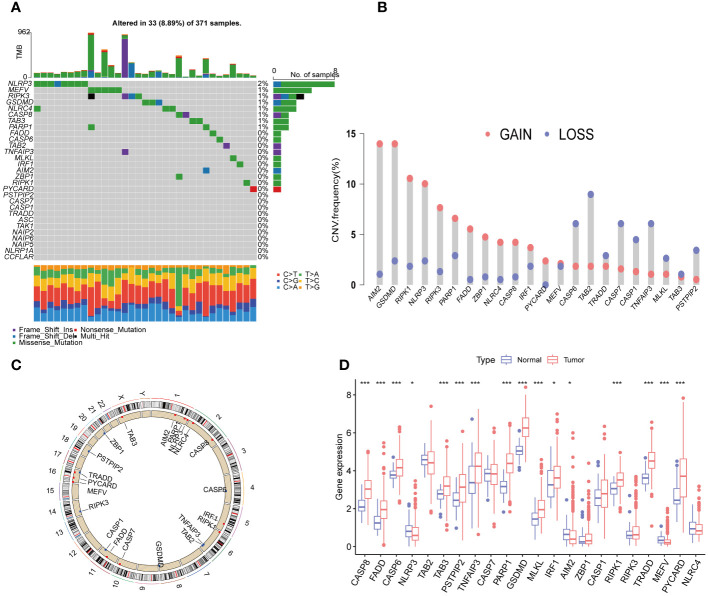
Expression and genetic alteration of PRG in HCC. **(A)** The maftool exhibited incidence of somatic mutations of PRG in 371 HCC patients from TCGA database; **(B)** The CNV frequency of PRG in TCGA cohort. The height of the column showed the proportions of gain or loss variations; **(C)** The location of CNV alteration of 22 PRG on 23 chromosomes. **(D)** The expression of 22 PRG in HCC and normal tissues;. PRGs, PANoptosis-related genes; HCC, hepatocellular carcinoma; CNV, Copy number variation. The p-values were showed as: *p < 0.05; ***p < 0.001.

We first collected a set of 29 PRGs from previously published studies (6, 8, 16). As shown in **Figure 2A**, 33 (8.89%) of 371 samples had somatic mutations. Among the 29 PRGs, NLRP3 and MEFV exhibited the highest mutation frequency. Copy number variation (CNV) analysis showed that AIM2, GSDMD, RIPK1, NLRP3, RIPK3, PARP1, FADD, ZBP1, NLRC4, CASP8, IRF1, PYCARD, and MEFV had the increased CNV, whereas, CASP6, TAB2, TRADD, CASP7, CASP1, TNFAIP3, MLKL, TAB3, and PSTPIP2 displayed decreased an CNV decrease (**Figure 2B**). The locations of the CNV alterations of PRGs on the chromosomes were shown in **Figure 2C**. Furthermore, we conducted mRNA differential expression analysis of these 29 PRGs between 374 HCC samples and 50 healthy samples from TCGA. The result showed that gene, including CASP8, FADD, CASP6, TAB3, PSTPIP2, TNFAIP3, PARP1, GSDMD, MKLK, IRF1, RIPK1, TRADD, PYCARD, was upregulated in HCC, whereas only NLRP3, AIM2, and MEFV were significantly downregulated in HCC samples (**Figure 2D**).

### Identification of PRGs clusters in HCC

**Figure 3:**
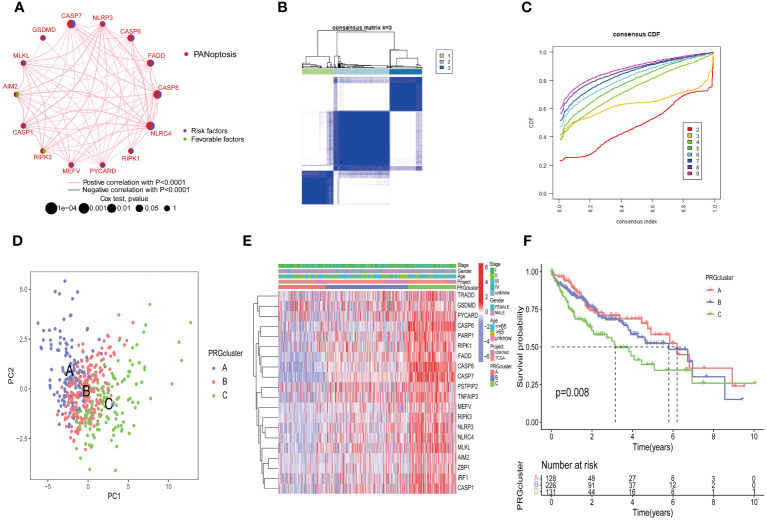
Identification of molecular subtypes of PRGs for HCC. **(A)** A network between PRGs in HCC; **(B, C)** Consensus matrix heatmap defining three clusters (k = 3) and their correlation area; **(D)** PCA diagram of HCC samples in cluster A, B, and C. **(E)** Complex heat maps show clinical correlations among the three clusters; **(F)** Survival analysis of three PRGclusters.

To explore the overall landscape of PRGs interaction, relationships, and prognostic significances, a network map was constructed (**Figure 3A**). The network map showed 14 of 29 genes showed significant correlation in interaction, relationship and prognostic. The relationship between the prognosis of HCC patients and 14 PRGs were assessed using the Kaplan-Meier curves and shown in **Supplementary Figure 1**. The expression of 20 PRGs in HCC were used to conduct an unsupervised clustering algorithm and group the 486 HCC patients into three distinct patterns. The most effective clustering result was achieved at K=3 among K = 2 to K = 9 (**Figures 3B, C**). Thus, we categorized 128 HCC patients into PRGcluster A, 226 into PRGcluster B, and the remaining 132 into PRGcluster C. The principal component analysis (PCA) indicated a satisfactory separation between the three clusters (**Figure 3D**). PRGcluster C exhibited higher expression levels for most PRGs, whereas PRGcluster A displayed lower expression levels for most PRGs (**Figure 3E**). Next, we investigated the relationship between these three PRGcluster and clinical characteristics. Kaplan-Meier curves demonstrated significant differences in OS among the three PRGclusters, with PRGcluster C showing the poorest OS (**Figure 3F**).

**Figure 4:**
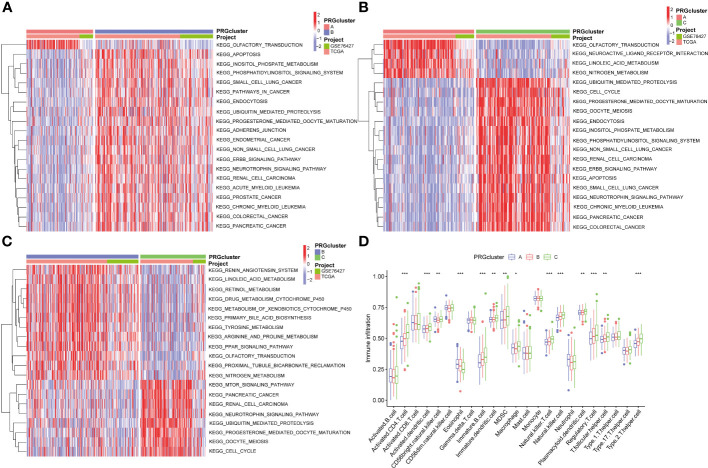
GSVA results between three PRGclusters and relationship of tumor microenvironment in three PRGclusters. **(A–C)** The GSVA heat map showed the differences in pathways in the three clusters; **(D)** The differential analyses between immune cells and the scale of fraction for PRGcluster A, B and C. *p<0.05, **p<0.01, ***p<0.001.

Additionally, we conducted the GSVA analysis to identify distinct pathways associated with PRGclusters A, B, and C (**Figures 4A–C**). The ssGSEA were utilized to assess the immune cell infiltrations in three PRGclusters. The boxplot showed that PRGcluster C was enriched in activated CD4 T cells, activated dendritic cells, CD56 bright nature killer cells, immature B cells, immature dendritic cells, MDSCs, macrophages, natural killer cells, plasmacytoid dendritic cells, regulatory T cells, T follicular helper cells, and type 2 T helper cells. While, PRGcluster A was enrich in eosinophils (**Figure 4D**).

### Generation of PRG signatures in HCC

**Figure 5:**
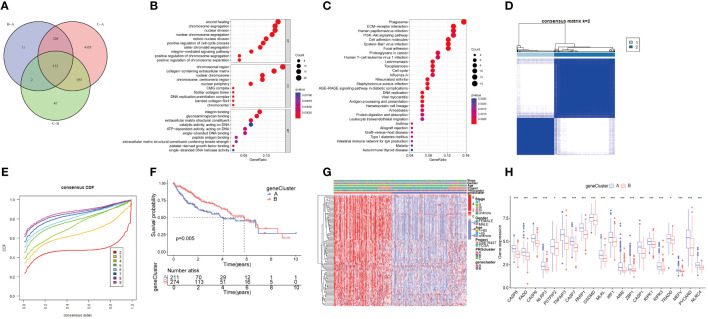
Functional enrichment analysis of PRGs, and identified two genecluster based on 153 DEGs. **(A)** The Venn diagram shows the intersection of three PRGclusters; **(B)** Analysis of BP, CC, and MF terms of GO enrichment demonstrated the possible function of the 153 DEGs; **(C)** Kyoto Encyclopedia of Genes and Genomes (KEGG) pathway enrichment analysis revealed the possible pathways; **(D)** Unsupervised cluster analysis of 153 DEGs developed two geneclusters (k = 2); **(E)** Consensus matrix heatmap defining two clusters and their correlation area; **(F)** Survival analysis of two geneclusters. **(G)** A complex heat map illustrated the expression patterns; **(H)** Expression of PRGs between genecluster A and genecluster B. *p<0.05, ***p<0.001. DEGs, differential expressed genes.

We conducted a differential gene expression analysis of three PRGclusters, comparing them in pairs three times among the three subtypes. We used a Venn diagram to successfully identify 153 DEGs exhibiting intersection across these three clusters (**Figure 5A**). The potential functions and pathways governed by these 153 DEGs were unraveled using GO and KEGG enrichment analyses performed using the “ClusterProfiler” packages. The GO results unveiled that these DEGs were involved in chromosome segregation, wound healing, and positive regulation of the cell cycle process in the biological process (BP). Within the Cellular Component (CC) category, they were prominently associated with chromosomal regions, collagen-containing extracellular matrices, and nuclear chromosomes. The Molecular Function (MF) exhibited closely related to integrin binding, platelet−derived growth factor binding, and single−stranded DNA binding (**Figure 5B**). Furthermore, the KEGG pathway analysis demonstrated their participation in processes such as Phagosome, PI3K-Akt signaling pathway, cell adhesion molecules, ECM-receptor interaction, Proteoglycans in cancer, and Cell cycle (**Figure 5C**).

To further analyze the important roles, univariate Cox regression was performed to identify the relationship between the 153 PRGcluster-related DEGs and the prognosis in HCC. Subsequently, patients were categorized into two major gene clusters, denoted as genecluster A and genecluster B (**Figures 5D, E**). The Kaplan–Meier analysis revealed that patient in genecluster B exhibited a more favorable OS rate compared to those in genecluster A (**Figure 5F**). A complex genecluster-based heatmap was developed by combining the gender, age, HCC clinical stage, PRGcluster, genecluster in TCGA and GSE 76427 (**Figure 5G**). Moreover, the analyzing the transcriptomic profile from the heatmap was analyzed that revealed the upregulation in most genes of genecluster A, whereas those in genecluster B predominantly exhibited downregulation. The DGEs analysis between genecluster A and B showed that CASP8, FADD, CASP6, NLRP3, PSTPIP2, TNFAIP3, CASP7, PARP1, GSDMD, MLKL, IRF1, AIM2, ZBP1, CASP1, RIPK1, RIPK3, TRADD, MEFV, PYCARD, NLRC4 were upregulated in genecluster A (**Figure 5H**).

### Construction of prognostic PANoptosis risk scoring model

**Figure 6:**
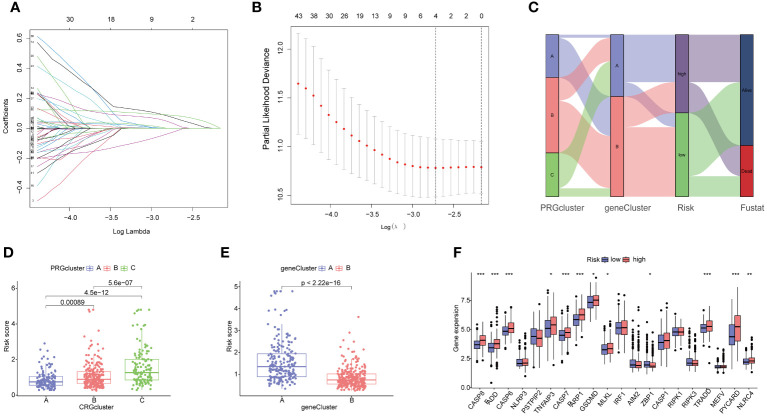
Identification of 4 genes for estimating the risk score and the relationship between molecular classifications, PRGs expression levels and the risk score. **(A, B)** The Least absolute shrinkage and selection operator (LASSO) regression analysis and partial likelihood deviance on the prognostic genes; **(C)** Sankey plot showed the correlation between PRGclusters, geneclusters, risk groups and survival status in HCC patients; **(D)** Boxplots indicate the differences in risk scores in three PRGclusters and **(E)** two geneclusters. **(F)** The differential analysis of PRGs expression in high- and low-risk groups. *p<0.05, **p<0.01, ***p<0.001.

The HCC patients were randomly divided into a training set (243 samples) and a testing set (242 samples) to explore the prognosis related PRG-related DEGs. A univariate Cox regression analysis was performed using the 153 DEPRGs along with survival data within the training datasets. Out of these, 93 DEPRGs were exhibited significant associations with prognosis (p< 0.05). To enhance the precision of gene selection for model construction, we adopted a systematic approach. Specifically, we randomly sampled 80% of the training set specimens for LASSO regression analysis, incorporating tenfold cross-validation and executing 1000 iterations. Subsequently, this rigorous methodology enabled the identification of a refined subset comprising 4 significant genes crucial for model refinement. (**Figures 6A, B**). Subsequently, we performed a multivariate Cox regression analysis using these four significant genes, and identifying the most pivotal genes for prognosis—CTSC, CDCA8, G6PD, and CXCL9The PANoptosis Risk scoring system was constructed based on the following formula in the training sets: Risk score=Exp (CTSC)× (0.215) + Exp (CDCA8) × (0.232) + Exp(G6PD) × (0.138) + Exp (CXCL9) × (-0.196). All set files were combined by the training group and testing group files. The HCC patients were subsequently categorized into high- and low-risk groups based on the median Risk score for each group. The Sankey diagram shows the distribution of PRGs risk scores with three PRGcluster, two geneclusters, and HCC patients survival status (**Figure 6C**). The boxplot showed that PRGcluster C and genecluster A had higher risk scores (**Figures 6D, E**). The differential expression analysis between high- and low-risk group demonstrated that CASP8, FADD, CASP6, TNFAIP3, CASP7, PARP1, GSDMD, MLKL, ZBP1, TRADD, PYCARD, and NLRC4 were upregulated in high-risk group (**Figure 6F**).

### Validation of prognostic PANoptosis risk scoring model

**Figure 7:**
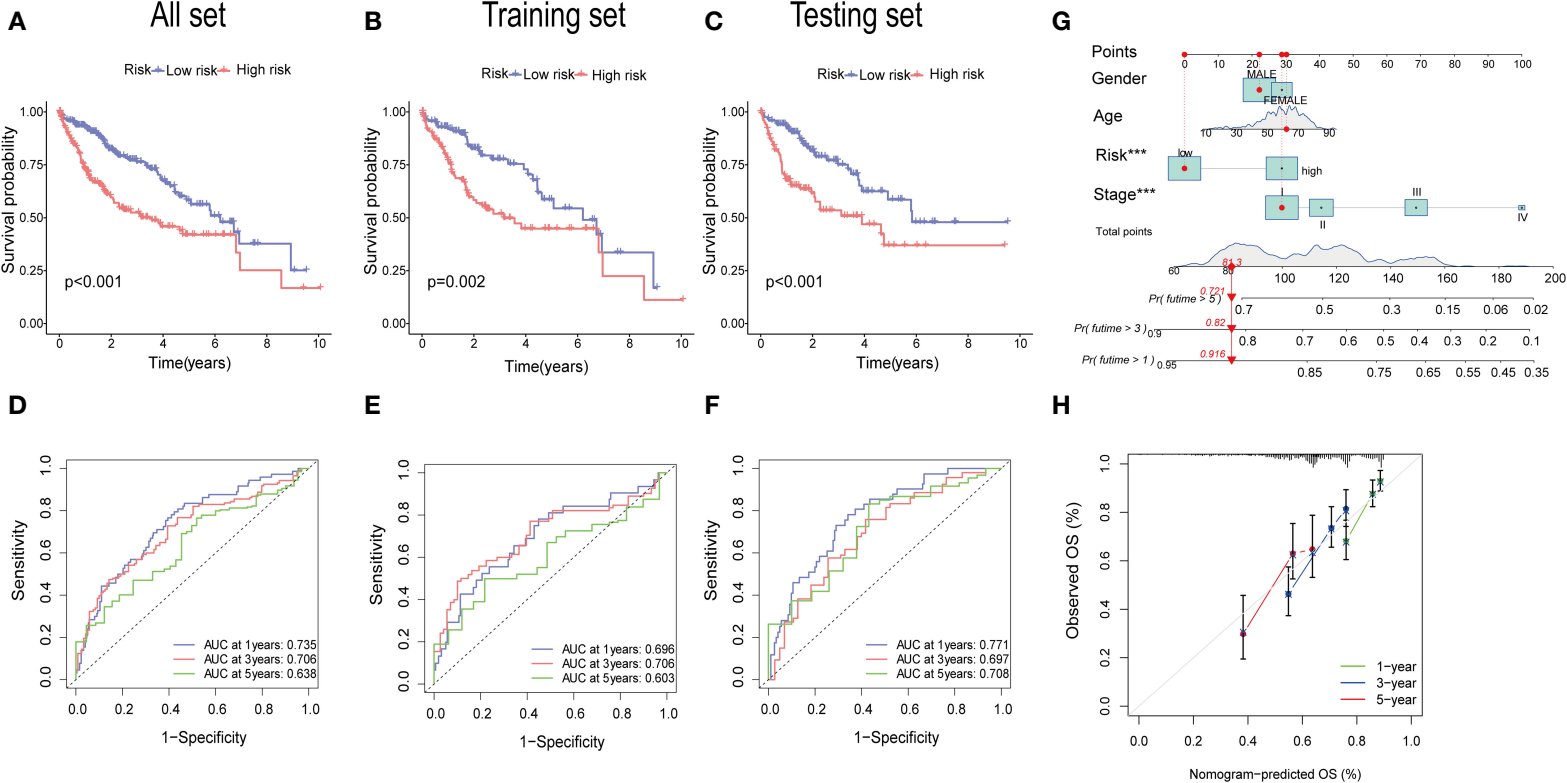
Validation of the prognostic value of the signatures. **(A–C)** K-M survival curve of all sets, testing set, and training set**. (D–F)** The ROS for 1-year, 3-year, and 5-year OS prediction of all sets, testing set, and training set. **(G)** The nomogram of the risk score and clinical features (age, gender, and stage) for predicting the survival of HCC patients. **(H)** The calibration curves showed the accuracy of the nomogram in the 1st, 3rd, and 5th years.

The KM analysis revealed that patients with low-risk had a better survival rate than those with high-risk in both total, training, and testing sets (P< 0.05) (**Figures 7A–C**). Additionally, we utilized the ROC curves to assess the prediction efficiency of the risk score. The AUCs for 1-, 3-, and 5-year survival rates in the training set were 0.696, 0.706, and 0.603, respectively. In total sets, the AUCs for 1-, 3-, and 5-year survival rates was 0.735, 0.706, 0.638, respectively. In testing sets, the AUCs of 1-, 3-, and 5-year survival rates were 0.771, 0.697, and 0.708, respectively (**Figures 7D–F**). These results indicated a favorable predictive performance for the survival of HCC patients. We next constructed a nomogram with using risk score, clinical stage, gender, and age (**Figure 7G**). the calibration curves indicated a relative link between observed and nomogram-predicted OS of HCC patients (**Figure 7H**), confirming the validity of the nomogram model for predicting the survival of HCC patients.

**Figure 8:**
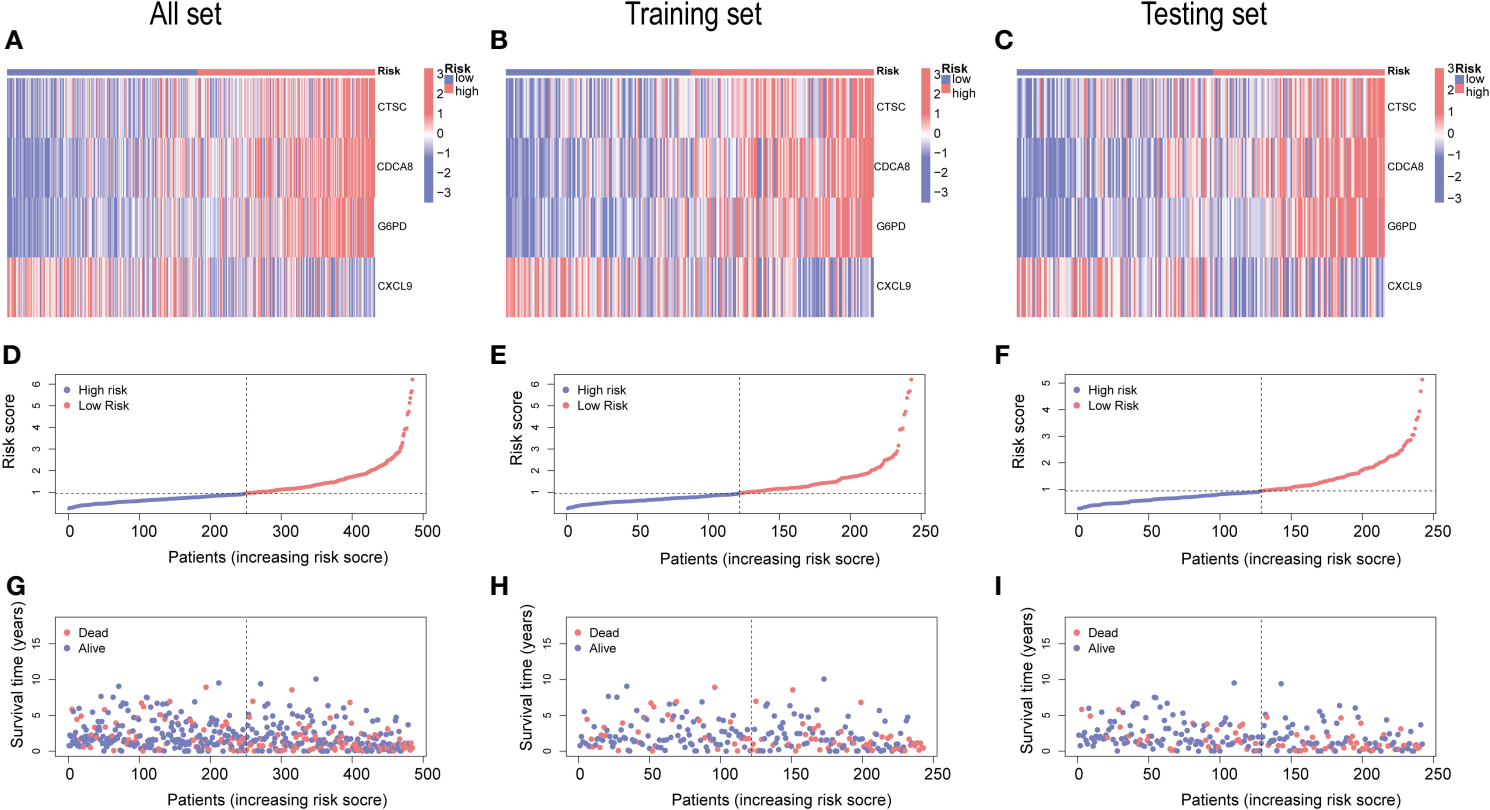
An analysis of risk gene expression and the distribution of risk scores, survival status of HCC patients. **(A–C)** Heatmap of four risk genes across different risk scores in the All, training, and testing sets, respectively. **(D–F)** Exhibition of PRGs risk score model of the All, training, and testing sets, respectively. **(G–I)** Survival status between low-and high-risk groups in the All, training, and testing sets, respectively.

The gene expression differences for CTSC, CDCA8, G6PD, and CXCL9 between high- and low-risk group in all set, training set, and testing set are depicted in **Figures 8A–C**. The heatmap visually represented that CTSC, CDCA8, and G6PD exhibited higher expression levels in the high-risk groups, whereas CXCL9 showed lower expression levels. We observed an inverse correlation between risk score and survival time, as well as a positive association between risk score and the death rate across all sets—total, training, and testing. These findings underscore that HCC patients with higher risk scores had poorer survival outcomes (**Figures 8D–I**).

### Relationship between signature and TME

**Figure 9:**
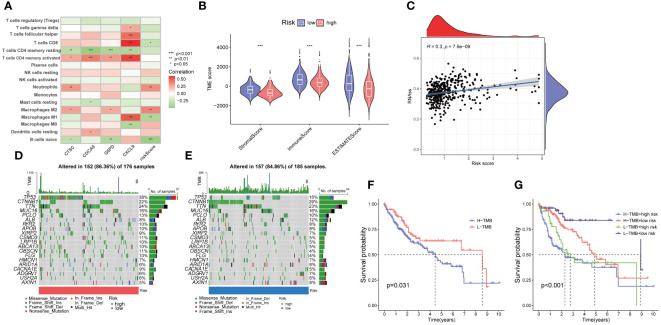
Evaluation of the tumor microenvironment, and tumor mutation burden (TMB) in low- and high-risk groups. **(A)** Correlation between the four risk genes and the abundance of immune cells. **(B)** Comparison of ESTIMATE scores, stromal scores, and immune scores between the low- and high-risk groups. **(C)** Correlation between the stem cell content and the PANoptosis risk score. **(D, E)** The frequency of somatic gene mutations in the high- and low-risk groups, respectively. **(F, G)** The Kaplan-Meier curve of the tumor mutation burden and risk scores versus the overall survival. *p<0.05, **p<0.01, ***p<0.001

The association analysis between immune cell abundance and the risk score showed that neutrophils and macrophages M2 were positively correlated with risk score, whereas CD8 T cells, macrophages M1, and naïve B cells were negatively related with risk score (**Supplementary Figure 2**). Furthermore, **Figure 9A** demonstrates the correlation between immune cells and the four risk genes. The CTSC displayed significant associations with neutrophils, macrophages M2, and CD4 memory resting T cells. In the low-risk group, the Stromal Score, Immune Score, and ESTIMATE score were significantly higher compared to the high-risk group (**Figure 9B**).

Cancer stem cells (CSCs) were thought to play an important role in the recurrence, metastasis, and identifying therapeutic target due to their differentiation and self-renewal capacity l (31). A correlation analysis between the risk score and stem cells unveiled a positive linear correlation between the risk score and stem cell content (R=0.3, p<.001) (**Figure 9C**).

Furthermore, we explored the disparity in tumor somatic mutations between the high- and low-risk groups using “maftools”. The top six mutated genes were TP53, CTNNB1, TTN, MUC16, PCLO, and ALB in both high- and low-risk groups (**Figures 9D, E**). In addition, we observed that patients with high TMB displayed a poorer overall survival rate (**Figure 9F**). The combination of TMB and risk score demonstrated that low risk plus low TMB had the best OS (**Figure 9G**).

### Drugs susceptibility analysis

We next investigated the predictive therapeutic effects in patients with HCC by assessing the relationship between the two risk groups and drug sensitivity. Our analysis revealed significant differences in drug responses between the high- and low-risk groups, with 56 drugs exhibiting noteworthy distinctions. Among them 16 drugs had lower IC50 in high-risk groups, such as Paclitaxel, Sepantronium, and Tozasertib. Low-risk group were more sensitive to Oxaliplatin, sorafenib, irinotecan (**Supplementary Table 4**).

### Validation of the expression levels signature genes

**Figure 10:**
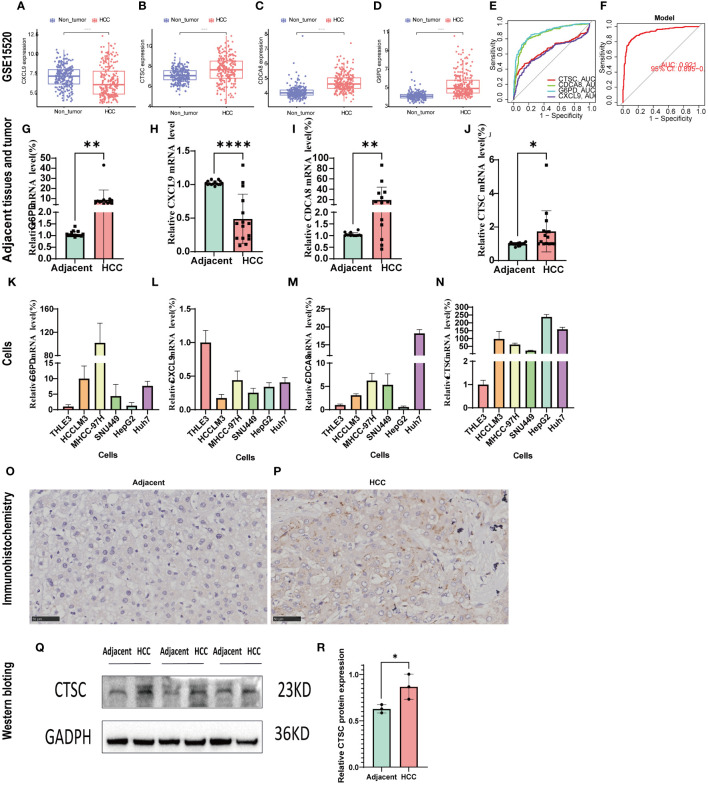
Validation of the signature genes with GSE14520, qRT-PCR, and IHC. **(A–D)** The expression of CXCL9, CTSC, CDCA8, G6PD between HCC and normal tissues in GSE14520; **(E)** The ROC results of 4 marker genes in GSE14520. The AUC value of CXCL9, CTSC, CDCA8, G6PD was 0.656,0.858,0.882,0.621, respectively. **(F)** ROC results of the 4-gene-based model based on 3-fold cross-validation in GSE14520. The AUC value as 0.921. AUC, area under curve; ROC, receiver operating characteristic; DCA, Decision curve analysis. **(G–J)** qRT-PCR confirmed the 4 marker genes expression between HCC tissues and adjacent tumor tissues; **(K–N)** qRT-PCR validated the 4 marker genes expression between HCC cells (HCCLM3, MHCC-97H, SUN449, HepG2, Huh7) and normal liver cell (THLE3). **(O, P)** CTSC representative IHC stained images in adjacent tissues and HCC tissue. **(Q, R)** Western blot analysis the protein expression in adjacent tumor tissues and HCC tissue. *p < 0.05; **p < 0.01; ****p < 0.0001.

GSE14520 was used to validated the mRNA expression and diagnosis probability. The results showed that CTSC, CDCA8, and G6PD were upregulated in HCC tissues, whereas CXCL9 was downregulated (**Figures 10A–D**). The AUC value of CTSC, CDCA8, G6PD, and CXCL9genes were 0.656, 0.858, 0.882, 0.621, respectively and the model AUC value reached to 0.92, suggesting our signature had higher quality of prediction (**Figures 10E, F**). In addition, we used RT-PCR to validated the mRNA expression of signature genes between adjacent tumor tissue and HCC, and normal liver cell THLE3 and liver cancer cell line of HCCLM3, MHCC-97H, SUN449, HepG2, and Huh7. Compared with the adjacent tumor tissue and most liver cancer cells lines, a significant increase expression of G6PD, CDCA8, and CTSC in HCC tissues and liver cancer cells was observed, whereas CXCL9 was significant downregulated (**Figures 10G–N**). However, the mRNA expression of G6PD and CDCA8 showed no significant differences between THLE3 and HepG2 (**Figures 10K, M**). IHC and western blotting further confirmed the higher expression of CTSC in HCC tissues compared to the adjacent tumor tissues (**Figures 10O–R**).

### Effects of CTSC on the proliferation and migration of SUN-449 cell

**Figure 11:**
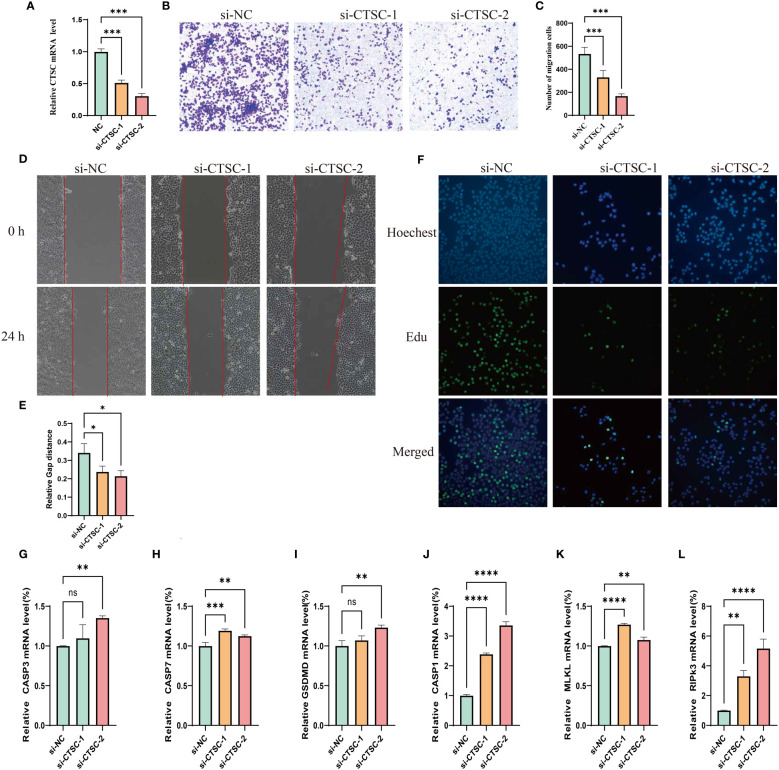
Cell model validation of CTSC in SUN-449 cell transfected with siCTSC and vector. **(A)** Relative CTSC mRNA level after being knocked down. **(B, C)** Transwell assays were employed to assess the ability of SUN-449 cell to migrate after CTSC was knocked down for 24 h; **(D, E)** Representative images and quantitative analysis of the results from the wound healing assay; **(F)** EdU assay was conducted between the si-NC and CTSC knockdown SUN-449 cells; **(G–L)** The mRNA expression of CASP3, CASP7, GSDMD, CASP1, MLKL, RIPK3 after CTSC was knocked down. *p < 0.05; **p < 0.01; ***p < 0.001; ****p < 0.0001. ns, not significant.

We designed three siRNA to study the impact of CTSC downregulation in SUN449 cells due to the upregulated expression of CTSC. qRT-PCR confirmed the CTSC effectiveness of downregulation following siRNA interference (**Figure 11A**). The results of Transwell and Wound-healing assays indicated the inhibition CTSC attenuated the migratory capabilities of SUN449 cell (**Figures 11B–E**). The EdU assay revealed a reduced proportion of EdU-positive cells upon the inhibition of CTSC in SUN449 cells, indicating that CTSC fosters the proliferation of HCC cells (**Figure 11F**). qRT-PCR result showed that inhibition of CTSC could increase the mRNA expression of CASP3, CASP7, GSDMD, CASP1, MLKL, RIPK3(**Figures 11G–L**).

## Discussion

HCC is a common fatal malignancy of the digestive system whose global burden has surged significantly from 1990 to 2019, posing substantial threats to human life, health, and the global economy (32). Despite previous efforts to diagnose and treat patients with HCC, a majority of them are diagnosed at advanced stages, rendering them ineligible for surgical resection and resulting in unfavorable prognoses. Therefore, it is imperative to elucidate the mechanism contributing to the pathogenesis of HCC to explore innovative approaches for diagnosis and treatment. PANoptosis, a component of the host’s innate immune response, has been identified as a novel mechanism governing inflammatory programmed cell death, encompassing pyroptosis, apoptosis, and necroptosis (6). Previous studies have demonstrated the significant role of PANoptosis in tumorigenesis and anti-tumor therapies (16). We identified a valid signature to assess the treatment and prognosis of HCC and developed a signature based on the concept of PANoptosis for HCC patients.

In our study, we used 29 PRGs to evaluate their somatic mutations, CNVs, DEGs. Our findings indicated that the majority of PRGs were significantly upregulated in HCC, with only NLRP3, AIM2, and MEFV demonstrating downregulation in HCC. Notably, NLRP3 and AIM2 were significantly correlated with HCC prognosis. Previous research has confirmed the downregulation of AIM2 expression in human HCC tissues compared to adjacent normal tissues. Furthermore, we revealed that patients with HCC with higher AIM2 expression exhibited improved overall survival rates (33), consistent with our analysis. Regarding NLRP3, it plays dual roles in HCC. On one hand, the NLRP3 inflammasome inhibits HCC development via pyroptosis, while on the other hand, it promotes HCC growth through the mediation of different signaling pathways (34). Additionally, we identified that 14 PRGs were significantly associated with the survival rate of HCC patients. Collectively, these results suggest that PANoptosis may indeed play a pivotal role in the context of HCC.

We initiated our study by conducting a comprehensive clustering analysis to identify the molecular subtype of PANoptosis. All HCC patients were categorized into three distinct PRGclusters. Notably, although PRGcluster C exhibited an overall high expression of most PRGs, it displayed experienced a significantly worse prognosis. Thus, higher expression levels of PRGs could be associated with a lower rate of survival. Additionally, PRGcluster C exhibited heightened immune infiltration, characterized by the presence of various immune cells such as activated CD4 T cells, immature dendritic cells, MDSCs, macrophages, natural killer cells, and regulatory T cells. Previous studies has indicated that certain components within TME, including dendritic cells, macrophages, and natural killer cells, can promote tumor proliferation, invasion, metastasis, and hinder anti-cancer immune responses (35–37). This finding implies that the elevated expression of PRGs could lead to increased immune cell infiltration and subsequently result in a poorer survival rates. Furthermore, we identified 153 DEGs related to PANoptosis among the three PRGclusters, and subsequently categorized patients into two geneclusters. Notably, geneCluster A exhibited higher PRG expression levels and a worse survival prognosis. Altogether, these findings provide valuable insights into the underlying biology of these specific tumor types and offer potential avenues for subgroup screening in HCC.

To improve the prognosis prediction and characterization capabilities of each patient with HCC, LASSO and multivariate Cox regression analyses were employed to construct a novel prognostic signature to better predict HCC prognosis. High-risk groups were characterized by elevated expression levels of most PRGs and poorer prognoses. Furthermore, PRGcluster C and geneCluster A, both associated with reduced survival rates, displayed higher risk scores. This reinforcing the correlation between higher risk scores in our established signature and unfavorable prognostic outcomes. Our risk model has practical applications in treatment personalization, increased surveillance frequency, and patient prognosis prediction. Specifically, high-risk patients may benefit from aggressive therapies, while more frequent monitoring and surveillance can aid in early disease detection. Moreover, our analysis encompassing ROC curves, nomograms, and calibration plots underscored the superior predictive performance and accuracy of the constructed signature. The 1-,3-,5-year AUC was 0.735, 0.706, 0.638 in the present model, while another study PANoptosis-related gene signature model showed 1-,3-,5-year AUC was 0.707, 0.622, and 0.562, respectively. This indicating that the efficiency of diagnosis of our model was superior than previous prognostic model (38).

Four risk gene (G6PD, CTSC, CDCA8, *and* CXCL9) were identified and utilized to calculate the risk score in our study. These four risk genes have been previously associated with various types of malignant tumors, including HCC. G6PD has been recognized as a prognostic signature and a potential treatment target for different tumors (39). Zeng et al. reported that the expression of G6PD in HCC tissues was upregulated compared to the corresponding adjacent normal tissues (39). In our qRT-PCR analysis, we confirmed the elevated expression of *G6PD* in HCC tissues and HCC cell lines. *G6PD* is known to promote HCC cell proliferation, invasion, migration and inhibit ferroptosis. Knockdown *G6PD* or inhibit it with smilax China root extract could suppresses HCC cell growth, tumorigenesis and metastasis (39–41). CDCA8, a crucial regulator of mitosis, is upregulated in numerous cancer types. A high expression of CDCA8 has been associated with higher AFP, larger tumor size, pathological status, T stage, and poor prognosis in HCC. Silencing CDCA8 could suppresses tumor growth, proliferation, and stemness of HCC by inactivating AKT/β–Catenin Signaling, and regulating the CDK1/cyclin B1 signaling axis (42–45). CXCL9, a specific ligand for CXCR3, facilitates tumor-suppressive lymphocytic infiltration in certain solid tumors coupled with its two family members CXCL10 and CXCL11 (46). Increasing evidence has demonstrated that CXCL9 is closely correlated with the prognosis of certain solid tumor patients, such as colorectal cancer lung cancer, and HCC (47). Ding et al. revealed that CXCL9 binding to CXCR3 promotes metastasis and invasion of CD133+ liver cancer cells via the p-ERK1/2-MMP2/MMP9 pathway (48). In addition, increasing the expression of CXCL9 with rhCXCL9 has been reported to enhance the HCC invasion ability by upregulating the PREX2 (49).

Cathepsin C(CTSC), a lysosomal cysteine protease abundantly expressed in multiple tissues and belonging to the papain superfamily, plays a pivotal role in numerous tumor biological processes. Moreover, CTSC regulates breast cancer lung metastases by modulating neutrophil infiltration and the formation of neutrophil extracellular traps (50). Silencing CTSC has the capacity to promote apoptosis, thereby restraining the growth of colorectal cancer. Furthermore, it can enhance colorectal cancer metastasis by modulating immune escape through the upregulation of CSF1 (51, 52). An earlier study has documented the pivotal role of cathepsin C in regulating pyroptosis and lysosome-mediated cell death within cathepsin C-deficient mouse splenocytes (53). For HCC, CTSC collaborates with the TNF-α/p38 MAPK Signaling Pathway to enhance proliferation and metastasis (54). In addition, our results also showed that inhibition CTSC could attenuated HCC cells metastasis and proliferation, confirming the previous results. This indicated that CTSC could be a target for HCC therapy.

Immunoreactivity plays a critical role in the development of tumors and offers a promising target for potential cancer therapies (55). Our risk score was negatively correlated with CD8 T cells, macrophages M1, and naïve B cells, and positively correlated with neutrophils, macrophages M2. A higher number of CD8+ T cell, macrophages M1, cases were positively associated with better OS and DFS in HCC patients, whereas macrophages M2 were related to a poor prognosis and outcome of HCC (56–59). This is consistent with our finding that the low-risk group had a better prognosis, as shown in our previous overall survival analysis. In the present study, we also explore the correlation among risk genes, risk score, and immune cells. The results showed that high-risk group associated with a lower Stromal Score, Immnune Score, and ESTIMATE score, and higher TMB. This suggests that our signature could predict the TME composition. These result of our study was aligned with a previous study based on cuproptosis-related genes (60). However, another model based on the immune-related gene was on the contrast, namely high-risk group have a higher Stromal Score, Immnune Score, and ESTIMATE score (61). CSCs, as a driver of tumor progression and growth, contribute to metastasis, recurrence, and drug resistance (62). A previous study indicated that a high immune score is indicative of improved chemotherapy and immunotherapy efficacy (63). In our research, we found the low-risk group displayed higher immune and lower stem cell content, implying a more favorable anti-tumor treatment. We found that TP53 and CTNNB1 genes were the most frequently mutated genes in both groups, which was consistent with previous study (64). Mutations in TP53 gene is regarded as a major driver of HCC, and higher mutation rate of TP53 was associated with poor overall survival (65). In our study, we found that high-risk group have higher mutation frequency of TP53 and poor prognosis, compared to low-risk group. Our study showed that Oxaliplatin, irinotecan, and sorafenib was more sensitivity in low-risk group, consistent with previous studies (66–68) supporting our risk model possesses the potential to predict the effectiveness of drugs treatment. In addition, one person can be stratified into high- or low-risk group and matching the most suitable personalized medicine through prediction based on based on the expression of risk gene of the person, then increasing the treatment effectiveness.

Nonetheless, our study had certain limitations. Firstly, the majority of our analyses relied on publicly available datasets and all samples were obtained retrospectively, which could have introduce cases selection bias and thus affected the accuracy of our finding. Hence, it is imperative to conduct well-designed prospective studies in order to validate the robustness and applicability of our findings. Secondly, although we conducted expression validation at both tissue and HCC cell levels, the sample size was relatively limited. We plane to are committed to expanding our sample collection efforts to assess this signature in the context of immunotherapy in the future. Thirdly, some crucial clinical variables such as surgical interventions, neoadjuvant chemotherapy, and tumor markers were not included in our study. Fourthly, although we have performed qRT-PCR to validate the relationship between CTSC and PANoptosis marker gene, more research, including Western blotting and IHC need conducted to confirm the result. Finally, although our prognostic model has some benefits, it has some barrier to clinical implementation. For example, the data availability and quality, and cost-effectiveness due to additional tests, monitoring. Consequently, our findings’ validity is relies on the inclusion of clinical cases.

## Conclusion

In conclusion, we have developed a pivotal PANoptosis-based molecular clustering approach and prognostic signature with multifaceted capabilities, including survival prediction, TMB assessment, and clinical therapy guidance. Our study has the potential to advance our understanding of PANoptosis in HCC and contribute to the development of more effective personalized immunotherapy or targeted therapy. Nonetheless, it is imperative to acknowledge the inherent limitations of this study, and further experiments and clinical case validations are warranted to substantiate our findings.

## Data availability statement

The datasets presented in this study can be found in online repositories. The names of the repository/repositories and accession number(s) can be found in the article/**Supplementary Material**.

## Ethics statement

The studies involving humans were approved by The Medical Ethics Committee of LiuZhou People’s Hospital Affiliated to Guangxi Medical University. The studies were conducted in accordance with the local legislation and institutional requirements. The participants provided their written informed consent to participate in this study. The animal studies were approved by The Medical Ethics Committee of LiuZhou People’s Hospital Affiliated to Guangxi Medical University, and all patients provided written informed consent in accordance with the Declaration of Helsinki. The studies were conducted in accordance with the local legislation and institutional requirements. Written informed consent was obtained from the owners for the participation of their animals in this study. Written informed consent was obtained from the individual(s) for the publication of any potentially identifiable images or data included in this article.

## Author contributions

GO: Data curation, Formal analysis, Validation, Writing – original draft, Funding acquisition, Project administration, Software, Writing – review & editing. QL: Validation, Formal analysis, Software, Writing – original draft. YW: Writing – original draft. WD: Writing – review & editing, Project administration. HD: Validation, Writing – review & editing, Project administration. YLL: Software, Writing – review & editing. JL: Writing – review & editing, Software. ML: Writing – review & editing, Software. SWL: Data curation, Writing – review & editing. SL: Data curation, Writing – review & editing. YYL: Writing – review & editing, Data curation. GP: Funding acquisition, Writing – review & editing, Data curation. JY: Writing – review & editing, Writing – original draft. TG: Data curation, Writing – original draft, Writing – review & editing.
