# Supplementary material for: Identification of PANoptosis-related subtypes, construction of a prognosis signature, and tumor microenvironment landscape of hepatocellular carcinoma using bioinformatic analysis and experimental verification
Source: Front Immunol. 2024 Apr 29;15:1323199. doi: 10.3389/fimmu.2024.1323199 (PMC11089137; doi:10.3389/fimmu.2024.1323199)
Supplement: Supplementary file 1 [file DataSheet_1.docx]

**Identification of PANoptosis-related subtypes, construction of a prognosis signature, and tumor microenvironment landscape of** **hepatocellular carcinoma using bioinformatic analysis and experimental verification**

| **Table S1 The target sites of siRNA for CTSC** | |
| --- | --- |
| **si-CTSC#1 sense：** | 5'- GCUACUCAUUUGCUUCUAUTT-3′ |
| **si-CTSC#1 antisense：** | 5'- AUAGAAGCAAAUGAGUAGCTT-3′ |
| **si-CTSC#2 sense：** | 5'- GCGGCUUCCCAUACCUUAUTT-3′ |
| **si-CTSC#2 antisense：** | 5'- AUAAGGUAUGGGAAGCCGCTT-3′ |
| **si-NC sense：** | 5-UUCUCCGAACGUGUC ACGUTT-3′ |
| **si-NC antisense：** | 5'-ACGUGACACGUUCGGAGAATT -3′ |

| **Table S2 the primers of GAPDH, POLD1, NFE2L2, and DLD** | | |
| --- | --- | --- |
| **Genes** | | **Sequences (5’-3’)** |
| **β-actin** | Forward | CACCATTGGCAATGAGCGGTTC |
|  | Reverse | AGGTCTTTGCGGATGTCCACGT |
| **CTSC** | Forward | CAGGAGGTTGTGTCTTGTAGCC |
|  | Reverse | GGAGAATCAGTGCCTGTGTAGG |
| **G6PD** | Forward | CTGTTCCGTGAGGACCAGATCT |
|  | Reverse | TGAAGGTGAGGATAACGCAGGC |
| **CDCA8** | Forward | CAGTGACTTGCAGAGGCACAGT |
|  | Reverse | CTCATTTGTGGGTCCGTATGCTG |
| **CXCL9** | Forward | CTGTTCCTGCATCAGCACCAAC |
|  | Reverse | TGAACTCCATTCTTCAGTGTAGCA |
| **GSDMD** | Forward | GCTTCCACTTCTACGATGCCATG |
|  | Reverse | GCCTGCGATCTTTGCCTGTC |
| **CASP7** | Forward | AGGGACCGAGCTTGATGATGG |
|  | Reverse | ACCGTGGAATAGGCGAAGAAG |
| **CASP3** | Forward | ACAGACAGTGGTGTTGATGATGAC |
|  | Reverse | ATGGCACAAAGCGACTGGATG |
| **RIPK3** | Forward | GGAGCAGGGCACAAGAAGAG |
|  | Reverse | AGGGCTGGGCATCTGGTTTC |
| **MLKL** | Forward | ACTTTGAGGCAGTCTTTGGAATCG |
|  | Reverse | GAGCCAGTCTTCACATTCTTCACC |
| **CASP1** | Forward | GGTGCTGAACAAGGAAGAGATGG |
|  | Reverse | TCGGAATAACGGAGTCAATCAAAGC |

| **Table S3. The prognostic values of 20 DEGs in HCC patients with a univariate Cox regression model.** | | | | |
| --- | --- | --- | --- | --- |
| **id** | HR | HR.95L | HR.95H | p-value |
| **CASP8** | 1.473867 | 1.124288 | 1.932143 | 0.004984 |
| **FADD** | 1.302987 | 1.036988 | 1.637218 | 0.023103 |
| **CASP6** | 1.347394 | 1.030213 | 1.762229 | 0.029457 |
| **NLRP3** | 1.30188 | 0.957268 | 1.770552 | 0.092649 |
| **PSTPIP2** | 0.886744 | 0.747817 | 1.05148 | 0.166799 |
| **TNFAIP3** | 1.081557 | 0.940532 | 1.243727 | 0.271389 |
| **CASP7** | 1.380128 | 1.102461 | 1.727728 | 0.004938 |
| **PARP1** | 1.195439 | 0.963795 | 1.482758 | 0.10429 |
| **GSDMD** | 1.079022 | 0.864778 | 1.346345 | 0.500645 |
| **MLKL** | 1.170903 | 0.897572 | 1.527469 | 0.24473 |
| **IRF1** | 1.006208 | 0.82886 | 1.221501 | 0.95012 |
| **AIM2** | 0.973928 | 0.77053 | 1.231016 | 0.825068 |
| **ZBP1** | 0.786382 | 0.54812 | 1.128214 | 0.191925 |
| **CASP1** | 1.070663 | 0.886647 | 1.29287 | 0.477948 |
| **RIPK1** | 1.201859 | 0.892167 | 1.619052 | 0.226496 |
| **RIPK3** | 0.824168 | 0.609882 | 1.113746 | 0.208124 |
| **TRADD** | 1.222964 | 0.923076 | 1.620278 | 0.160826 |
| **MEFV** | 1.349404 | 0.709082 | 2.567956 | 0.361355 |
| **PYCARD** | 1.033336 | 0.920061 | 1.160557 | 0.579887 |
| **NLRC4** | 1.622165 | 1.128519 | 2.331746 | 0.008974 |

| **Table S4. Drug sensitivity analysis** | | |
| --- | --- | --- |
| **Drugs** | **Low risk group (P value)** | **High risk group (P value)** |
| **Acetalax** | 3.7e−05 |  |
| **AMG-319** | 0.00069 |  |
| **AZD1208** | 3.5e−07 |  |
| **AZD2014** | 1.5e−13 |  |
| **AZD6482.** | 7.5e−07 |  |
| **AZD8186** | 6.2e−06 |  |
| **BI-2536** |  | 5.9e−05 |
| **Bortezomib** |  | 0.00044 |
| **BPD-00008900** |  | 1.8e−05 |
| **Camptothecin** | 0.00068 |  |
| **Cediranib** |  | 3e−05 |
| **Dactinomycin** | 0.00018 |  |
| **Daporinad** |  | 9.2e−07 |
| **Doramapimod** | p＜2.22e-16 |  |
| **Entinostat** | 8.2e-05 |  |
| **EPZ004777** | 0.00045 |  |
| **EPZ5676** | 0.00085 |  |
| **Fludarabine** | 4.2e-06 |  |
| **GDC0810** |  | 8.8e-08 |
| **.IAP_5620** | 2.1e-10 |  |
| **Irinotecan** | 6.2e-08 |  |
| **JAK_8517** | 0.00068 |  |
| **.JAK1_8709** | 5.1e-13 |  |
| **KU-55933** | 3.5e-05 |  |
| **LCL161** | 2e-05 |  |
| **LJI308** | 1.9e-10 |  |
| **Mitoxantrone** | 1.6e-09 |  |
| **MK-1775** |  | 2.2e-11 |
| **ML323** |  | 4.1e-08 |
| **Niraparib** | 1.2e-07 |  |
| **NU7441** | 2.2e-06 |  |
| **Nutlin-3a (-)** | 2.2e-12 |  |
| **Obatoclax Mesylate** | 5.5e-06 |  |
| **Olaparib** | 3.9e-07 |  |
| **Oxaliplatin** | 2.1e-10 |  |
| **Paclitaxel** |  | 3e-05 |
| **PCI-34051** | 3.2e-07 |  |
| **PD0325901** | 0.00015 |  |
| **Picolinici-acid** | 0.00033 |  |
| **PLX-4720** | 0.00022 |  |
| **PRIMA-1MET** | 0.00054 |  |
| **SB216763** | 6.8e-09 |  |
| **SB505124** | 1.3e-09 |  |
| **Selumetinib** | 1.4e-09 |  |
| **Sepantronium bromide** |  | 1.4e-11 |
| **Talazoparib** | 0.00028 |  |
| **Topotecan** | 0.00016 |  |
| **Tozasertib** |  | 9.6e-11 |
| **UMI-77** |  | 0.00081 |
| **Uprosertib** | 1.9e-07 |  |
| **Vinblastine** |  | 1.8e-05 |
| **Wee1 Inhibitor** |  | 2.5e-07 |
| **WEHI-539** |  | 0.00083 |
| **WZ4003** | 1.1e-05 |  |
| **XAV939** | 8.4e-08 |  |
| **YK-4-279** |  | 0.0002 |
|  |  |  |

| **Table 5 The risk score of GSE14520 based on the four risk genes** | | | | | |
| --- | --- | --- | --- | --- | --- |
|  | G6PD | CDCA8 | CXCL9 | CTSC | Risk_score |
| **GSM362947** | 4.173 | 5.873 | 4.721 | 6.924 | 287.9044 |
| **GSM362948** | 5.07 | 5.079 | 5.672 | 8.343 | 905.4124 |
| **GSM362949** | 4.293 | 4.542 | 6.224 | 7.536 | 335.933 |
| **GSM362950** | 6.348 | 4.84 | 7.93 | 8.465 | 583.7508 |
| **GSM362952** | 4.414 | 4.354 | 6.09 | 5.251 | -16.0601 |
| **GSM362954** | 7.982 | 5.717 | 5.844 | 7.934 | 1006.881 |
| **GSM362956** | 6.516 | 6.308 | 4.682 | 7.738 | 692.6444 |
| **GSM363420** | 9.069 | 5.758 | 9.016 | 8.49 | 703.9262 |
| **GSM363422** | 7.728 | 5.377 | 7.09 | 6.87 | 335.456 |
| **GSM363424** | 4.182 | 4.026 | 4.21 | 5.646 | 69.71575 |
| **GSM363426** | 5.912 | 4.558 | 4.756 | 7.393 | 399.6066 |
| **GSM363428** | 5.136 | 5.457 | 4.632 | 10.169 | 5665.337 |
| **GSM363430** | 5.263 | 5.773 | 4.637 | 8.071 | 769.0612 |
| **GSM363432** | 4.95 | 4.505 | 4.488 | 6.093 | 118.2286 |
| **GSM363436** | 4.592 | 4.94 | 5.498 | 5.673 | 60.72712 |
| **GSM363438** | 5.147 | 4.262 | 9.351 | 8.306 | -1345.5 |
| **GSM363440** | 4.916 | 4.839 | 4.666 | 4.412 | 45.03652 |
| **GSM363442** | 4.993 | 5 | 4.645 | 8.859 | 1547.424 |
| **GSM363444** | 6.049 | 4.266 | 4.774 | 7.197 | 338.9061 |
| **GSM363446** | 5.971 | 4.892 | 5.894 | 8.06 | 694.4069 |
| **GSM363448** | 4.888 | 4.69 | 7.708 | 6.464 | -254.799 |
| **GSM362958** | 4.945 | 5.047 | 6.082 | 7.323 | 295.3139 |
| **GSM362959** | 5.761 | 4.376 | 9.58 | 8.523 | -1693.05 |
| **GSM362960** | 5.248 | 4.083 | 5.496 | 6.988 | 225.2041 |
| **GSM362964** | 4.981 | 4.116 | 7.952 | 7.491 | -137.319 |
| **GSM362965** | 5.261 | 4.93 | 5.091 | 7.968 | 647.5545 |
| **GSM362966** | 5.1 | 4.263 | 4.633 | 6.929 | 238.5757 |
| **GSM362970** | 4.729 | 4.896 | 8.599 | 6.076 | -923.305 |
| **GSM362971** | 4.685 | 3.538 | 8.854 | 7.269 | -1040.98 |
| **GSM362972** | 4.098 | 3.959 | 8.635 | 5.982 | -996.868 |
| **GSM362976** | 4.198 | 4.101 | 7.222 | 8.092 | 457.496 |
| **GSM362977** | 4.249 | 4.608 | 4.984 | 6.044 | 94.9422 |
| **GSM362978** | 3.962 | 4.399 | 6.368 | 7.265 | 219.2026 |
| **GSM362982** | 8.365 | 4.994 | 7.201 | 7.375 | 707.0745 |
| **GSM362983** | 6.01 | 5.355 | 3.799 | 8.389 | 1042.246 |
| **GSM362984** | 5.262 | 4.071 | 10.374 | 7.471 | -5857.36 |
| **GSM362986** | 5.446 | 4.49 | 10.661 | 8.54 | -7208.75 |
| **GSM362987** | 4.83 | 4.491 | 4.736 | 7.975 | 640.7184 |
| **GSM362988** | 4.452 | 4.991 | 7.019 | 7.551 | 235.9691 |
| **GSM362992** | 5.24 | 5.098 | 6.281 | 8.477 | 991.9354 |
| **GSM362993** | 4.401 | 4.707 | 5.478 | 6.075 | 83.515 |
| **GSM362994** | 4.271 | 4.992 | 5.005 | 4.7 | 38.44121 |
| **GSM363008** | 6.558 | 5.095 | 8.839 | 8.44 | -221.752 |
| **GSM363009** | 7.5 | 6.041 | 9.729 | 8.654 | -1712.73 |
| **GSM363010** | 5.428 | 4.447 | 7.224 | 9.491 | 2628.928 |
| **GSM363011** | 7.919 | 4.803 | 7.263 | 9.07 | 1996.524 |
| **GSM363012** | 4.999 | 3.987 | 5.597 | 6.25 | 91.49167 |
| **GSM363013** | 7.663 | 4.243 | 9.283 | 9.124 | 174.2751 |
| **GSM363014** | 4.242 | 4.293 | 5.327 | 5.979 | 71.1706 |
| **GSM363015** | 4.339 | 4.067 | 5.486 | 7.159 | 253.236 |
| **GSM363016** | 4.36 | 3.916 | 7.371 | 7.848 | 261.4889 |
| **GSM363017** | 6.907 | 4.719 | 5.01 | 8.797 | 1556.601 |
| **GSM363029** | 6.246 | 4.431 | 8.412 | 8.466 | 229.8966 |
| **GSM363030** | 4.886 | 4.911 | 4.856 | 8.375 | 957.0991 |
| **GSM363031** | 5.166 | 6.098 | 6.715 | 8.118 | 686.9493 |
| **GSM363032** | 7.139 | 4.401 | 10.169 | 9.3 | -2567.58 |
| **GSM363033** | 6.434 | 5.434 | 10.025 | 8.709 | -2985.11 |
| **GSM363034** | 4.245 | 4.408 | 7.333 | 7.162 | 6.041353 |
| **GSM363035** | 4.703 | 4.39 | 7.226 | 7.465 | 139.8421 |
| **GSM363036** | 4.522 | 4.82 | 3.743 | 5.243 | 73.86854 |
| **GSM363037** | 4.933 | 5.544 | 4.714 | 7.756 | 558.7643 |
| **GSM363038** | 3.988 | 4.048 | 4.402 | 7.408 | 359.2998 |
| **GSM363039** | 4.178 | 4.587 | 7.002 | 5.1 | -148.321 |
| **GSM363048** | 7.314 | 5.806 | 6.911 | 7.345 | 420.5286 |
| **GSM363049** | 5.235 | 4.886 | 6.786 | 7.503 | 272.9942 |
| **GSM363050** | 7.106 | 4.576 | 5.811 | 6.324 | 245.2632 |
| **GSM363051** | 4.311 | 5.223 | 8.771 | 9.736 | 2427.057 |
| **GSM363052** | 9.004 | 6.269 | 9.287 | 8.976 | 829.8785 |
| **GSM363053** | 4.3 | 5.898 | 4.796 | 8.271 | 911.3731 |
| **GSM363054** | 5.461 | 3.805 | 4.943 | 7.792 | 535.9696 |
| **GSM363055** | 5.188 | 4.375 | 5.253 | 7.286 | 319.5238 |
| **GSM363056** | 6.505 | 4.864 | 4.026 | 7.824 | 648.7954 |
| **GSM363057** | 5.907 | 4.149 | 6.013 | 8.206 | 772.8396 |
| **GSM363069** | 5.274 | 4.765 | 6.39 | 8.677 | 1198.65 |
| **GSM363070** | 8.576 | 5.672 | 7.879 | 10.208 | 6112.179 |
| **GSM363071** | 5.49 | 4.733 | 4.255 | 6.912 | 261.9001 |
| **GSM363072** | 5.59 | 5.625 | 9.099 | 8.824 | -191.199 |
| **GSM363073** | 4.926 | 6.275 | 4.565 | 6.741 | 305.3906 |
| **GSM363074** | 5.051 | 4.416 | 7.252 | 7.873 | 328.6804 |
| **GSM363075** | 4.747 | 4.676 | 7.015 | 8.407 | 785.4529 |
| **GSM363076** | 6.12 | 5.647 | 4.005 | 8.455 | 1127.953 |
| **GSM363077** | 4.728 | 4.415 | 5.08 | 6.72 | 181.4698 |
| **GSM363078** | 5.08 | 5.093 | 7.515 | 9.716 | 3265.112 |
| **GSM363079** | 4.568 | 4.343 | 4.074 | 7.889 | 593.1945 |
| **GSM363080** | 7.573 | 5.149 | 6.473 | 8.704 | 1477.274 |
| **GSM363081** | 10.118 | 5.676 | 4.204 | 9.393 | 6055.793 |
| **GSM363082** | 5.083 | 4.123 | 5.097 | 8.773 | 1392.892 |
| **GSM363083** | 6.459 | 4.733 | 9.866 | 8.946 | -2010.72 |
| **GSM363084** | 4.754 | 3.638 | 6.054 | 6.636 | 105.2136 |
| **GSM363085** | 5.115 | 4.68 | 9.053 | 8.417 | -654.158 |
| **GSM363086** | 4.463 | 5.086 | 4.01 | 4.939 | 68.70697 |
| **GSM363087** | 4.282 | 4.277 | 5.306 | 8.379 | 923.4466 |
| **GSM363098** | 5.489 | 6.168 | 8.96 | 7.68 | -916.421 |
| **GSM363099** | 6.095 | 5.095 | 10.979 | 8.669 | -10141.1 |
| **GSM363100** | 6.771 | 4.908 | 4.91 | 8.674 | 1382.684 |
| **GSM363101** | 4.502 | 5.312 | 6.753 | 7.02 | 132.1271 |
| **GSM363102** | 4.818 | 4.301 | 3.772 | 7.156 | 301.2503 |
| **GSM363104** | 4.437 | 4.159 | 6.642 | 6.175 | -20.4199 |
| **GSM363105** | 4.523 | 4.662 | 5.578 | 9.079 | 1870.794 |
| **GSM363106** | 6.312 | 4.041 | 7.088 | 8.535 | 948.8568 |
| **GSM363107** | 4.908 | 4.323 | 5.765 | 7.917 | 563.5235 |
| **GSM363108** | 3.947 | 4.373 | 9.222 | 7.519 | -1561.26 |
| **GSM363109** | 7.874 | 5.307 | 3.876 | 9.448 | 3126.82 |
| **GSM363115** | 4.209 | 4.115 | 7.612 | 7.981 | 255.9657 |
| **GSM363121** | 5.466 | 3.79 | 9.705 | 9.249 | -936.63 |
| **GSM363122** | 4.839 | 4.944 | 5.238 | 6.419 | 144.9653 |
| **GSM363123** | 5.196 | 5.432 | 4.243 | 7.245 | 365.5401 |
| **GSM363124** | 4.305 | 3.916 | 4.705 | 7.558 | 412.1522 |
| **GSM363125** | 5.18 | 3.812 | 7.558 | 9.177 | 1738.978 |
| **GSM363126** | 4.593 | 4.869 | 6.289 | 6.3 | 55.35183 |
| **GSM363127** | 6.136 | 5.619 | 4.614 | 9.411 | 2735.701 |
| **GSM363128** | 5.119 | 4.35 | 6.468 | 6.365 | 39.72831 |
| **GSM363129** | 4.663 | 5.099 | 4.493 | 9.928 | 4441.822 |
| **GSM363130** | 3.942 | 4.019 | 4.51 | 5.296 | 45.09951 |
| **GSM363142** | 7.614 | 4.748 | 4.666 | 6.981 | 516.9101 |
| **GSM363143** | 4.163 | 4.24 | 8.164 | 8.253 | 161.9905 |
| **GSM363144** | 6.21 | 4.472 | 5.973 | 8.114 | 730.3212 |
| **GSM363145** | 5.367 | 5.2 | 10.963 | 8.883 | -9687.64 |
| **GSM363146** | 8.684 | 6.022 | 12.125 | 9.557 | -32195.6 |
| **GSM363147** | 6.377 | 4.589 | 5.165 | 8.382 | 1008.75 |
| **GSM363148** | 4.415 | 4.036 | 4.945 | 7.139 | 267.9449 |
| **GSM363149** | 3.797 | 5.298 | 4.346 | 7.208 | 327.7011 |
| **GSM363150** | 6.417 | 5.432 | 4.94 | 9.362 | 2612.213 |
| **GSM363151** | 5.098 | 5.058 | 5.106 | 6.4 | 156.1327 |
| **GSM363152** | 4.241 | 4.517 | 4.895 | 5.261 | 46.06531 |
| **GSM363164** | 4.44 | 4.859 | 4.448 | 5.355 | 70.36104 |
| **GSM363166** | 4.726 | 5.178 | 6.236 | 6.625 | 118.6398 |
| **GSM363168** | 4.174 | 4.19 | 4.312 | 4.466 | 28.37099 |
| **GSM363169** | 5.606 | 4.673 | 5.439 | 8.494 | 1067.604 |
| **GSM363170** | 5.09 | 5.07 | 5.828 | 8.579 | 1136.3 |
| **GSM363172** | 4.054 | 4.728 | 6.818 | 7.378 | 199.0932 |
| **GSM363174** | 4.801 | 4.21 | 8.132 | 7.495 | -247.511 |
| **GSM363176** | 4.356 | 4.321 | 7.363 | 7.372 | 61.23662 |
| **GSM363178** | 4.714 | 4.455 | 4.714 | 7.725 | 500.3125 |
| **GSM363180** | 4.205 | 4.008 | 8.945 | 7.728 | -992.918 |
| **GSM363182** | 4.676 | 4.367 | 5.122 | 6.766 | 186.8169 |
| **GSM363184** | 4.833 | 5.859 | 9.301 | 9.476 | 756.8467 |
| **GSM363186** | 4.277 | 4.196 | 6.933 | 6.771 | 11.85732 |
| **GSM363188** | 4.346 | 4.602 | 6.735 | 7.013 | 107.7338 |
| **GSM363190** | 5.573 | 5.352 | 5.738 | 8.352 | 935.7465 |
| **GSM363192** | 5.393 | 5.073 | 5.441 | 5.84 | 96.08105 |
| **GSM363194** | 7.128 | 5.182 | 6.735 | 11.025 | 13247.22 |
| **GSM363196** | 4.358 | 3.895 | 8.433 | 8.582 | 268.2879 |
| **GSM363198** | 7.42 | 4.656 | 7.596 | 7.316 | 188.0511 |
| **GSM363200** | 4.827 | 5.21 | 6.866 | 8.021 | 526.228 |
| **GSM363202** | 4.614 | 4.324 | 7.422 | 5.856 | -221.245 |
| **GSM363204** | 5.174 | 4.454 | 5.94 | 6.301 | 87.05147 |
| **GSM363205** | 4.298 | 4.475 | 8.231 | 7.322 | -380.233 |
| **GSM363207** | 6.596 | 4.422 | 7.459 | 8.476 | 811.8314 |
| **GSM363209** | 4.22 | 4.276 | 9.77 | 8.342 | -2501.83 |
| **GSM363211** | 4.777 | 4.167 | 6.611 | 9.113 | 1836.265 |
| **GSM363213** | 4.339 | 4.104 | 4.178 | 5.153 | 49.02816 |
| **GSM363215** | 4.872 | 4.419 | 8.471 | 8.07 | -211.104 |
| **GSM363217** | 4.78 | 4.295 | 3.779 | 7.079 | 280.0281 |
| **GSM363218** | 8.757 | 5.324 | 7.505 | 9.382 | 3121.085 |
| **GSM363220** | 5.414 | 4.515 | 9.524 | 9.829 | 1361.415 |
| **GSM363222** | 6.172 | 5.268 | 6.253 | 8.671 | 1263.036 |
| **GSM363224** | 4.515 | 4.048 | 6.582 | 8.641 | 1101.073 |
| **GSM363226** | 4.415 | 3.851 | 5.954 | 6.837 | 147.1196 |
| **GSM363230** | 4.785 | 5.32 | 4.058 | 6.181 | 156.5428 |
| **GSM363232** | 4.529 | 4.501 | 8.594 | 8.703 | 269.9598 |
| **GSM363235** | 8.694 | 5.765 | 9.593 | 9.409 | 646.228 |
| **GSM363237** | 6.885 | 5.338 | 10.032 | 9.814 | -342.479 |
| **GSM363239** | 4.335 | 5.181 | 6.421 | 7.731 | 421.0755 |
| **GSM363241** | 4.379 | 4.503 | 5.701 | 6.306 | 91.10431 |
| **GSM363243** | 4.195 | 4.1 | 9.25 | 7.457 | -1643.77 |
| **GSM363245** | 7.218 | 6.444 | 9.869 | 9.638 | -155.628 |
| **GSM363247** | 5.372 | 4.845 | 5.947 | 6.784 | 174.1812 |
| **GSM363249** | 5.069 | 4.839 | 6.026 | 6.038 | 60.19742 |
| **GSM363251** | 7.442 | 4.487 | 5.826 | 6.669 | 358.9558 |
| **GSM363263** | 8.819 | 5.602 | 7.276 | 7.984 | 1343.426 |
| **GSM363264** | 4.657 | 4.594 | 4.46 | 8.13 | 750.4068 |
| **GSM363265** | 5.03 | 4.459 | 9.169 | 8.816 | -390.107 |
| **GSM363266** | 5.581 | 5.117 | 7.896 | 7.704 | 25.4629 |
| **GSM363267** | 4.328 | 4.905 | 8.456 | 8.454 | 129.1114 |
| **GSM363268** | 7.647 | 6.127 | 8.855 | 8.562 | 145.7211 |
| **GSM363269** | 4.543 | 5.052 | 4.451 | 5.622 | 91.87315 |
| **GSM363270** | 4.281 | 4.637 | 4.664 | 7.223 | 307.8197 |
| **GSM363271** | 6.138 | 4.846 | 10.628 | 9.276 | -5700.47 |
| **GSM363272** | 3.921 | 3.97 | 6.788 | 7.908 | 429.9493 |
| **GSM363273** | 4.291 | 4.038 | 7.879 | 8.301 | 371.5539 |
| **GSM363274** | 4.894 | 4.421 | 4.794 | 8.549 | 1123.787 |
| **GSM363275** | 4.007 | 4.345 | 10.232 | 7.326 | -5092.37 |
| **GSM363288** | 4.049 | 4.044 | 6.147 | 7.156 | 205.1374 |
| **GSM363289** | 4.539 | 4.713 | 3.746 | 5.633 | 90.54942 |
| **GSM363290** | 4.347 | 4.526 | 9.572 | 6.796 | -2589.63 |
| **GSM363291** | 5.703 | 5.002 | 9.911 | 8.338 | -2975.05 |
| **GSM363292** | 4.411 | 5.017 | 5.708 | 8.608 | 1164.525 |
| **GSM363293** | 5.642 | 4.201 | 6.238 | 7.149 | 227.7459 |
| **GSM363294** | 4.587 | 4.368 | 5.939 | 8.909 | 1548.086 |
| **GSM363295** | 4.673 | 4.583 | 6.828 | 5.91 | -64.2434 |
| **GSM363296** | 5.127 | 4.675 | 4.293 | 7.802 | 559.568 |
| **GSM363297** | 8.132 | 5.402 | 10.844 | 9.679 | -6084 |
| **GSM363298** | 4.879 | 4.182 | 9.99 | 7.976 | -3615.18 |
| **GSM363309** | 7.972 | 6.165 | 8.801 | 8.003 | -148.382 |
| **GSM363310** | 5.288 | 5.84 | 5.681 | 7.549 | 457.8492 |
| **GSM363311** | 5.125 | 5.131 | 4.665 | 6.793 | 233.3414 |
| **GSM363312** | 4.227 | 4.759 | 4.981 | 6.275 | 122.1632 |
| **GSM363313** | 5.016 | 4.71 | 5.896 | 6.992 | 209.2112 |
| **GSM363314** | 5.132 | 5.451 | 10.421 | 9.797 | -2634.1 |
| **GSM363315** | 4.512 | 4.424 | 3.913 | 5.802 | 93.27493 |
| **GSM363316** | 4.265 | 5.869 | 4.171 | 5.035 | 112.2729 |
| **GSM363317** | 6.545 | 5.372 | 6.92 | 8.771 | 1333.14 |
| **GSM363326** | 5.005 | 4.711 | 10.778 | 8.713 | -8045.12 |
| **GSM363327** | 3.838 | 4.919 | 4.952 | 5.978 | 95.28465 |
| **GSM363328** | 6.013 | 4.549 | 5.618 | 7.972 | 647.5777 |
| **GSM363329** | 4.597 | 4.54 | 5.684 | 6.634 | 141.2872 |
| **GSM363330** | 5.646 | 4.225 | 4.798 | 8.988 | 1752.552 |
| **GSM363331** | 6.848 | 5.315 | 10.226 | 8.946 | -3584.16 |
| **GSM363332** | 4.719 | 6.443 | 7.571 | 6.357 | -95.2744 |
| **GSM363333** | 5.246 | 4.176 | 4.771 | 7.286 | 332.002 |
| **GSM363335** | 3.832 | 3.92 | 5.803 | 6.308 | 71.15242 |
| **GSM363336** | 4.058 | 4.231 | 9.875 | 7.919 | -3194.92 |
| **GSM363337** | 4.409 | 4.352 | 7.185 | 9.114 | 1723.266 |
| **GSM363339** | 5.243 | 4.796 | 6.656 | 8.003 | 544.6469 |
| **GSM363341** | 6.295 | 4.887 | 4.951 | 7.711 | 557.8774 |
| **GSM363343** | 4.787 | 4.15 | 5.936 | 6.504 | 100.6772 |
| **GSM363344** | 4.338 | 4.389 | 4.775 | 4.713 | 29.9743 |
| **GSM363346** | 5.231 | 4.59 | 6.696 | 7.678 | 354.5214 |
| **GSM363348** | 4.624 | 4.399 | 4.768 | 9.269 | 2289.765 |
| **GSM363350** | 6.806 | 5.732 | 6.386 | 7.653 | 532.9146 |
| **GSM363352** | 6.394 | 3.972 | 6.842 | 8.759 | 1280.412 |
| **GSM363354** | 9.439 | 6.47 | 7.462 | 8.744 | 2891.813 |
| **GSM363355** | 8.459 | 4.728 | 7.088 | 7.301 | 761.0956 |
| **GSM363357** | 4.925 | 5.074 | 4.163 | 8.27 | 883.0459 |
| **GSM363358** | 5.326 | 4.599 | 4.589 | 7.74 | 526.318 |
| **GSM363360** | 4.122 | 4.829 | 5.208 | 6.01 | 89.32627 |
| **GSM363362** | 4.804 | 3.951 | 5.422 | 7.512 | 377.9577 |
| **GSM363364** | 4.607 | 4.201 | 10.213 | 7.395 | -4962.73 |
| **GSM363366** | 4.212 | 4.335 | 7.631 | 6.021 | -288.378 |
| **GSM363368** | 5.71 | 4.751 | 8.703 | 7.296 | -794.611 |
| **GSM363371** | 4.091 | 4.487 | 10.05 | 7.864 | -3950.26 |
| **GSM363376** | 6.207 | 4.451 | 9.011 | 8.962 | 159.7941 |
| **GSM363378** | 4.012 | 4.15 | 4.896 | 6.115 | 93.43462 |
| **GSM363384** | 6.42 | 5.406 | 8.825 | 8.21 | -406.15 |
| **GSM363386** | 5.267 | 4.997 | 6.854 | 8.158 | 625.943 |
| **GSM363388** | 5.917 | 4.699 | 10.634 | 9.748 | -4380.97 |
| **GSM363391** | 4.508 | 4.218 | 6.439 | 7.598 | 334.3751 |
| **GSM363393** | 4.663 | 4.025 | 6.211 | 7.567 | 345.6283 |
| **GSM363400** | 4.129 | 5.451 | 4.62 | 6.793 | 234.4237 |
| **GSM363404** | 3.997 | 4.35 | 4.019 | 7.068 | 266.9471 |
| **GSM712542** | 3.878 | 5.819 | 6.79 | 8.521 | 989.6416 |


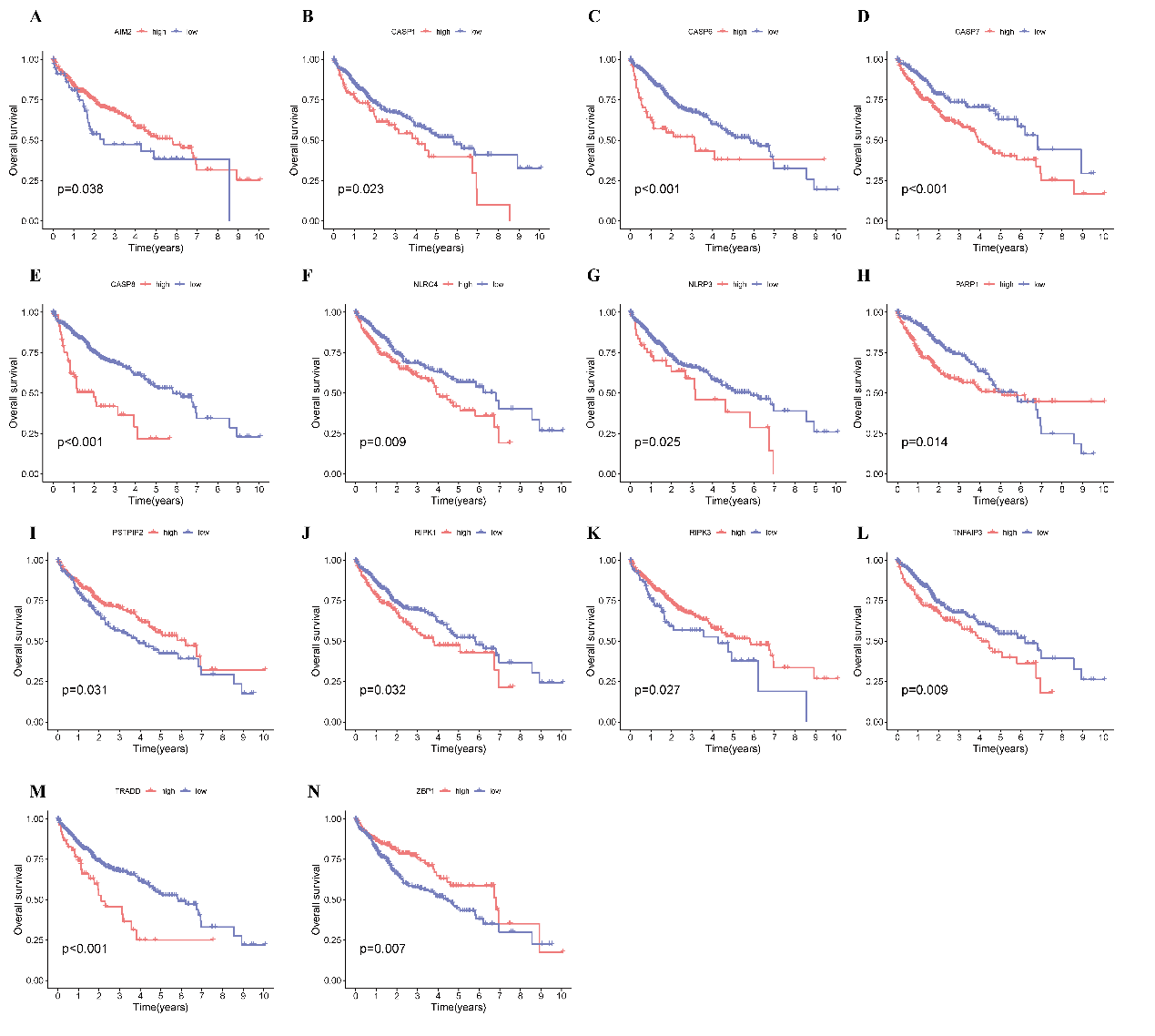


**Figure S1** Prognosis significance of PANoptosis-related genes of HCC patients. (A-K) K-M survival curve displays the HCC OS of different genes (AIM2, CASP1, CASP6, CASP7, CASP8, NLRC4, NLRP3, PARP1, PSTPIP2, RIPK1, RIPK3, TNFAIP3, TRADD, ZBP1).


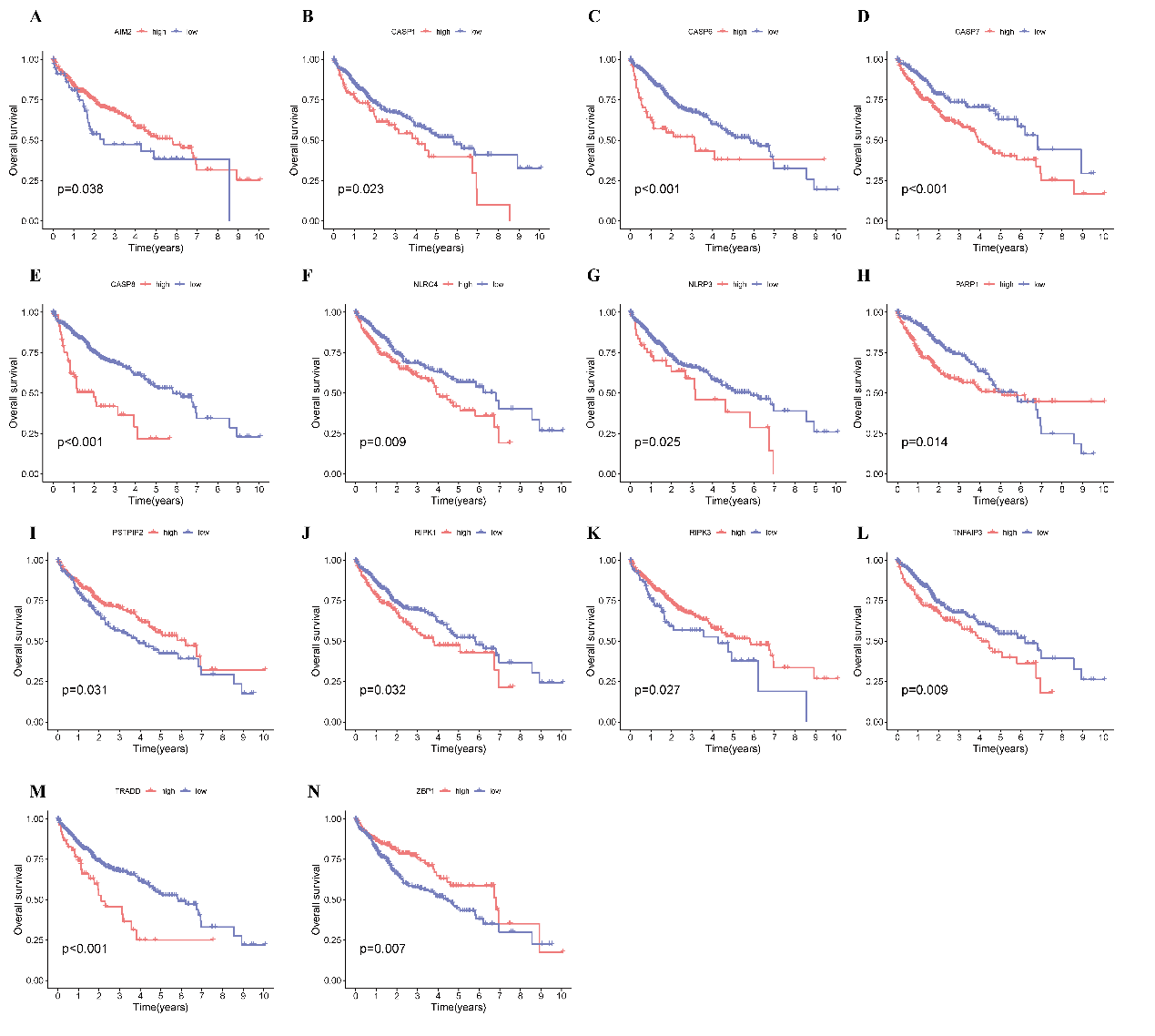


**Figure S2** Evaluation of tumor microenvironment in high- and low-risk groups. (A-E) Relationship between risk score and different immune cell types.


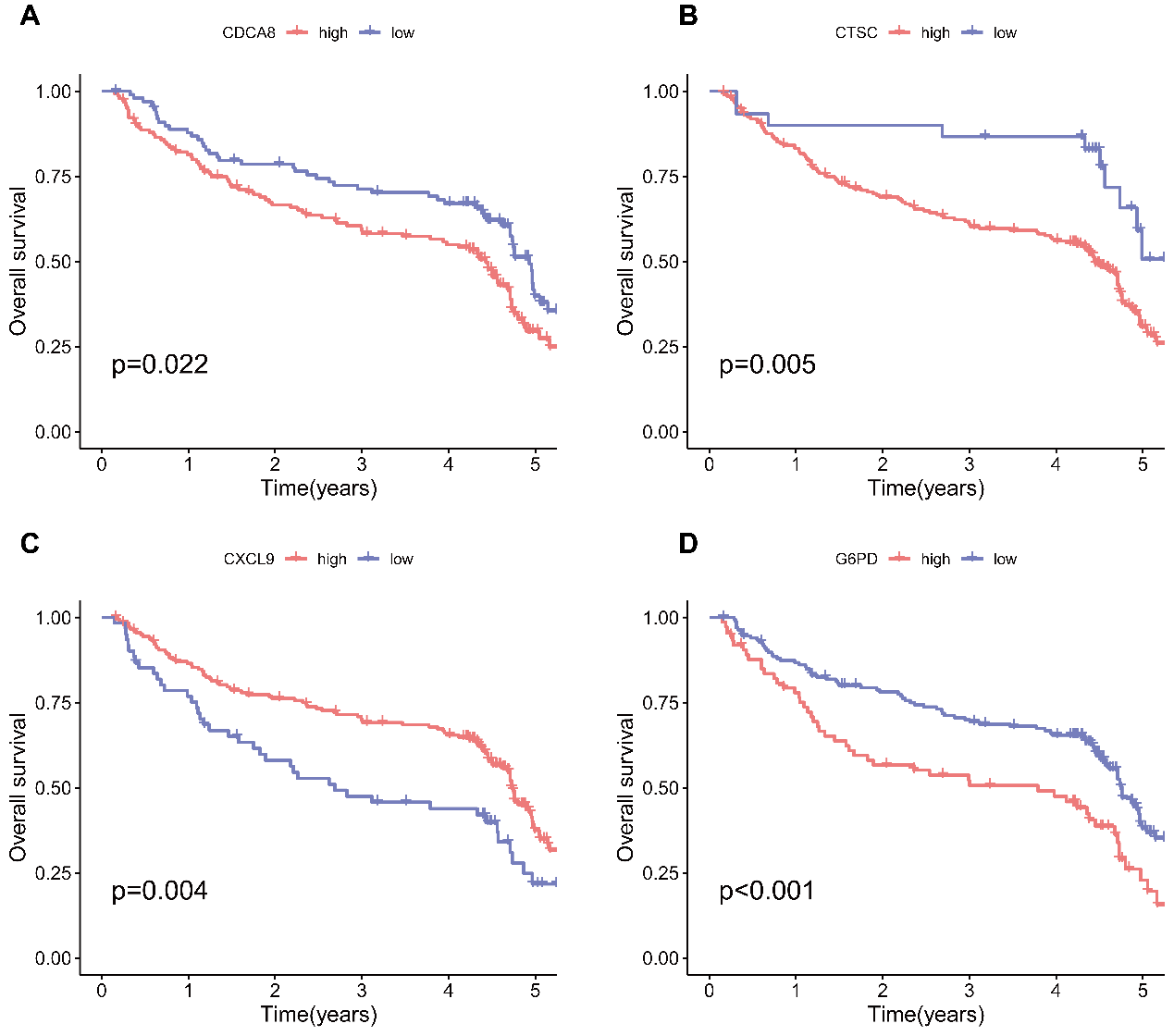


**Figure S3** Prognosis significance of risk genes of HCC patients in GSE14520. (A-K) K-M survival curve displays the HCC OS of different genes (CDCA8, CTSC, CXCL9, G6PD).


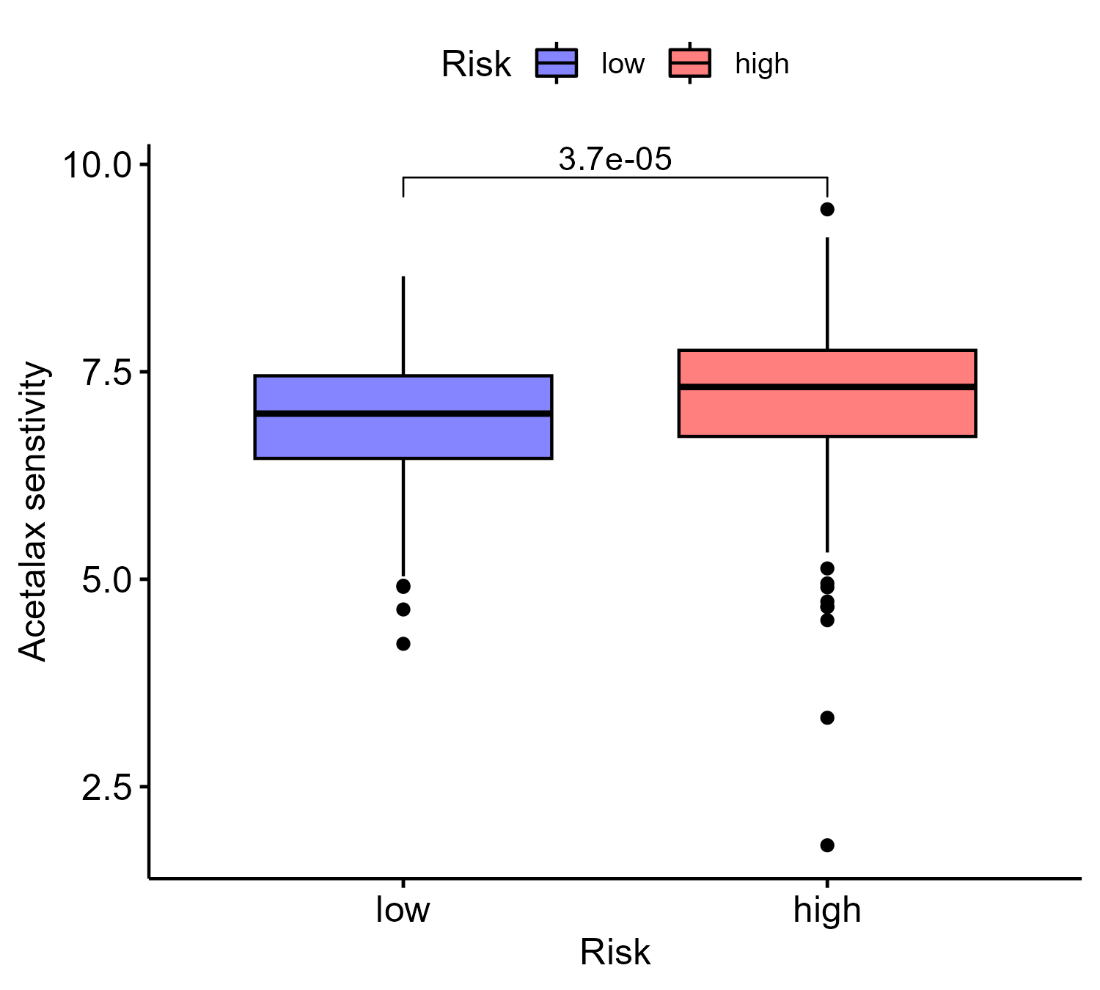


**Figure S4** Significant IC50 difference from **Acetalax** between high- and low-risk subgroups


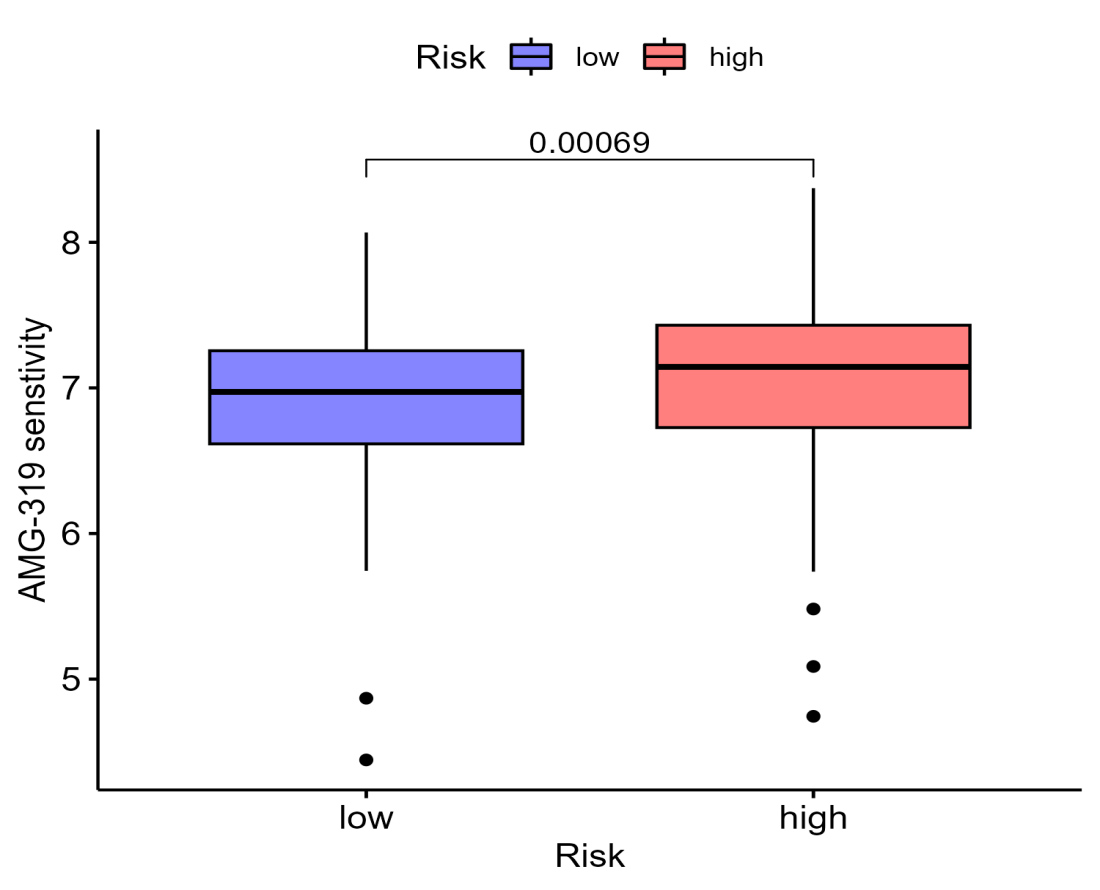


**Figure S5** Significant IC50 difference from AMG-319 between high- and low-risk subgroups


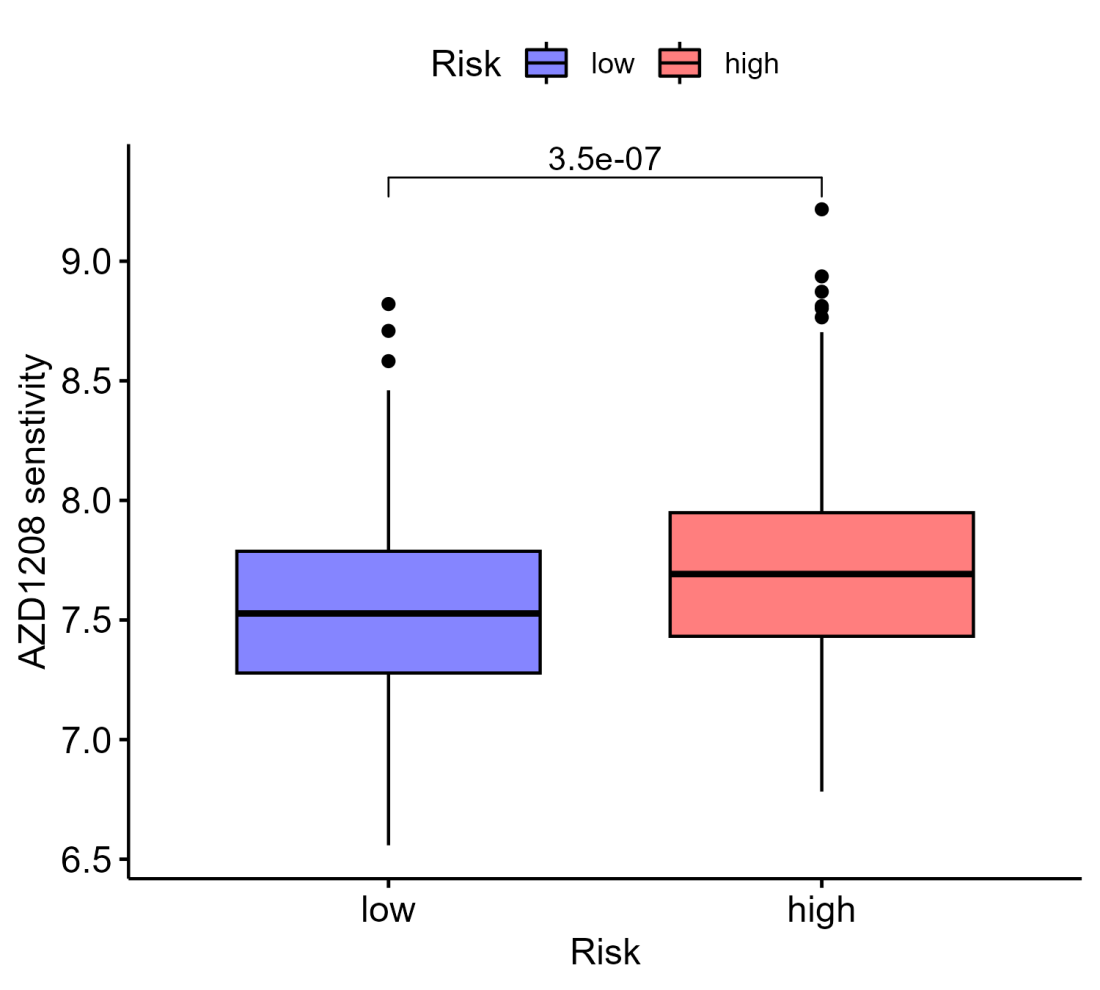


**Figure S6** Significant IC50 difference from AZD1208 between high- and low-risk subgroups


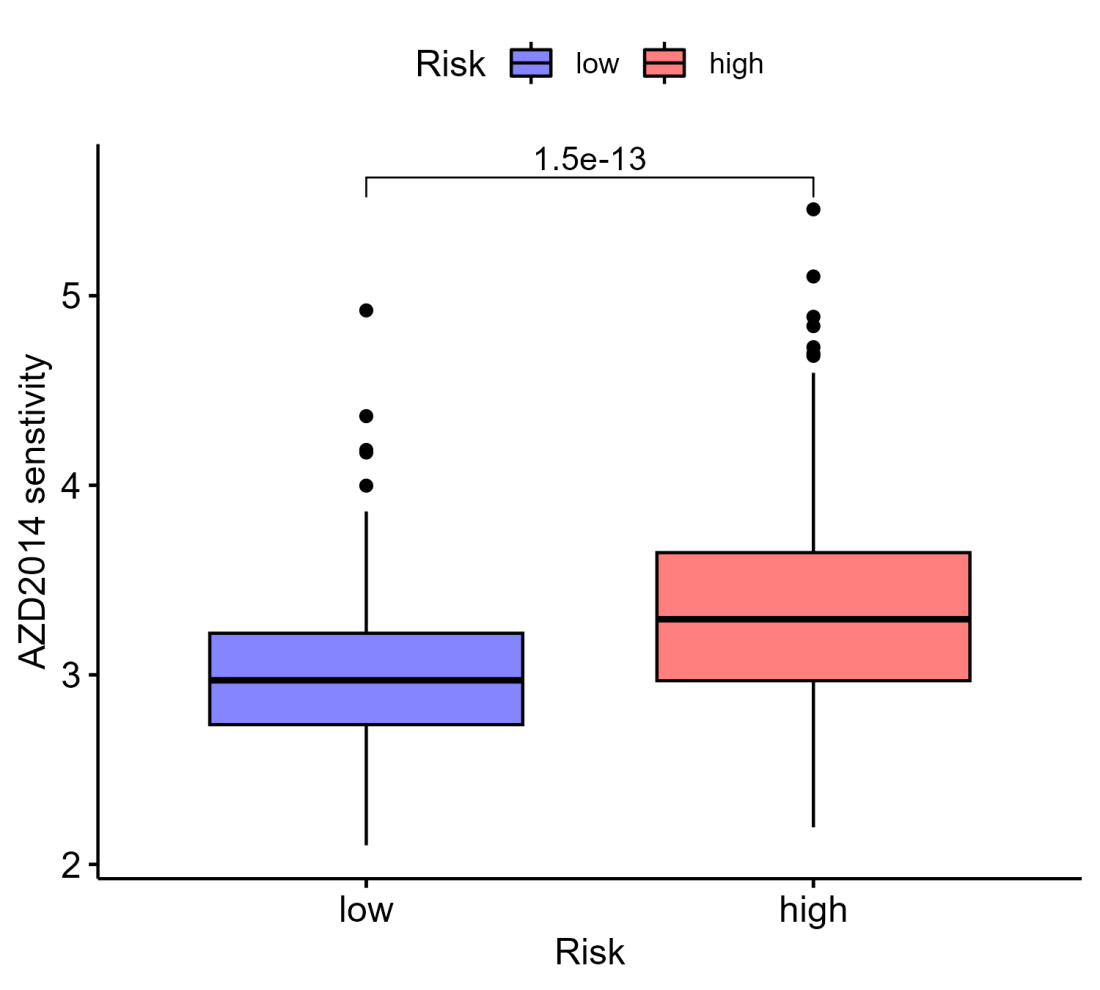


**Figure S7** Significant IC50 difference from AZD2014 between high- and low-risk subgroups


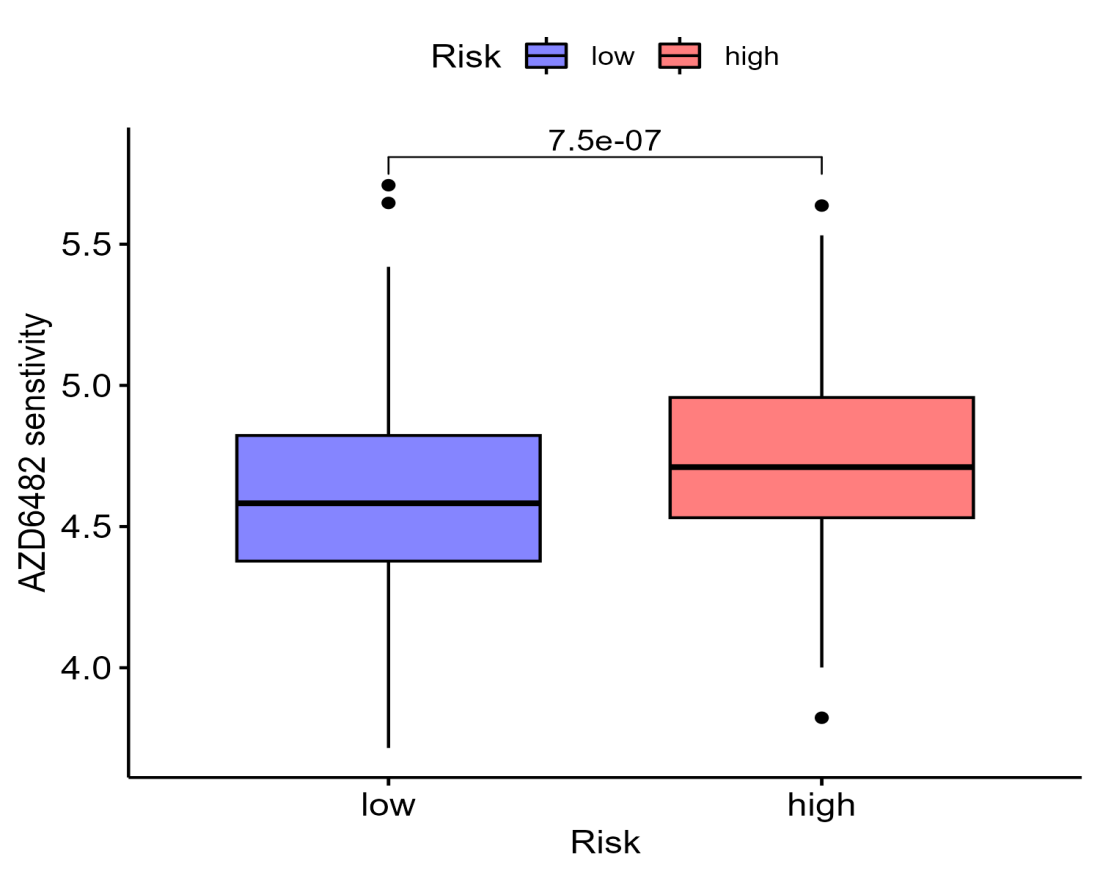


**Figure S8** Significant IC50 difference from AZD6482 between high- and low-risk subgroups


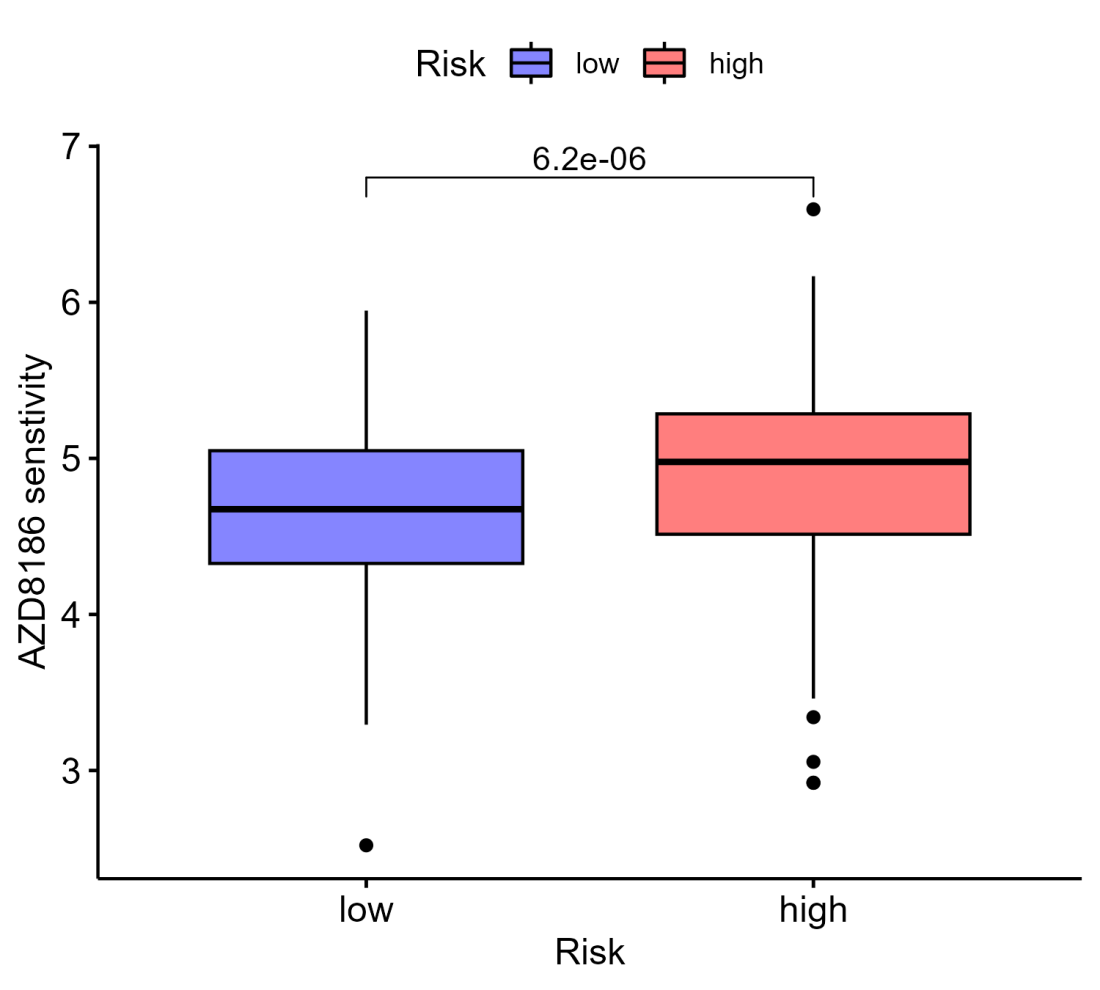


**Figure S9** Significant IC50 difference from AZD8186 between high- and low-risk subgroups


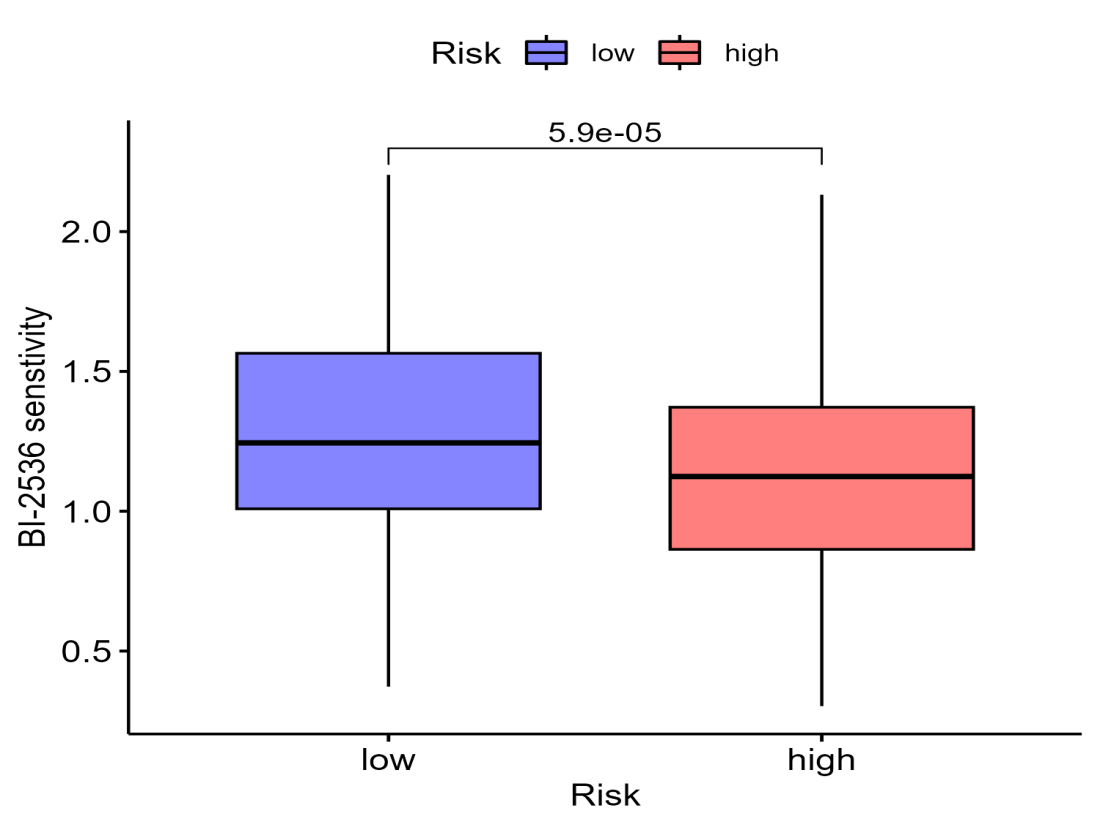


**Figure S10** Significant IC50 difference from BI-2536 between high- and low-risk subgroups


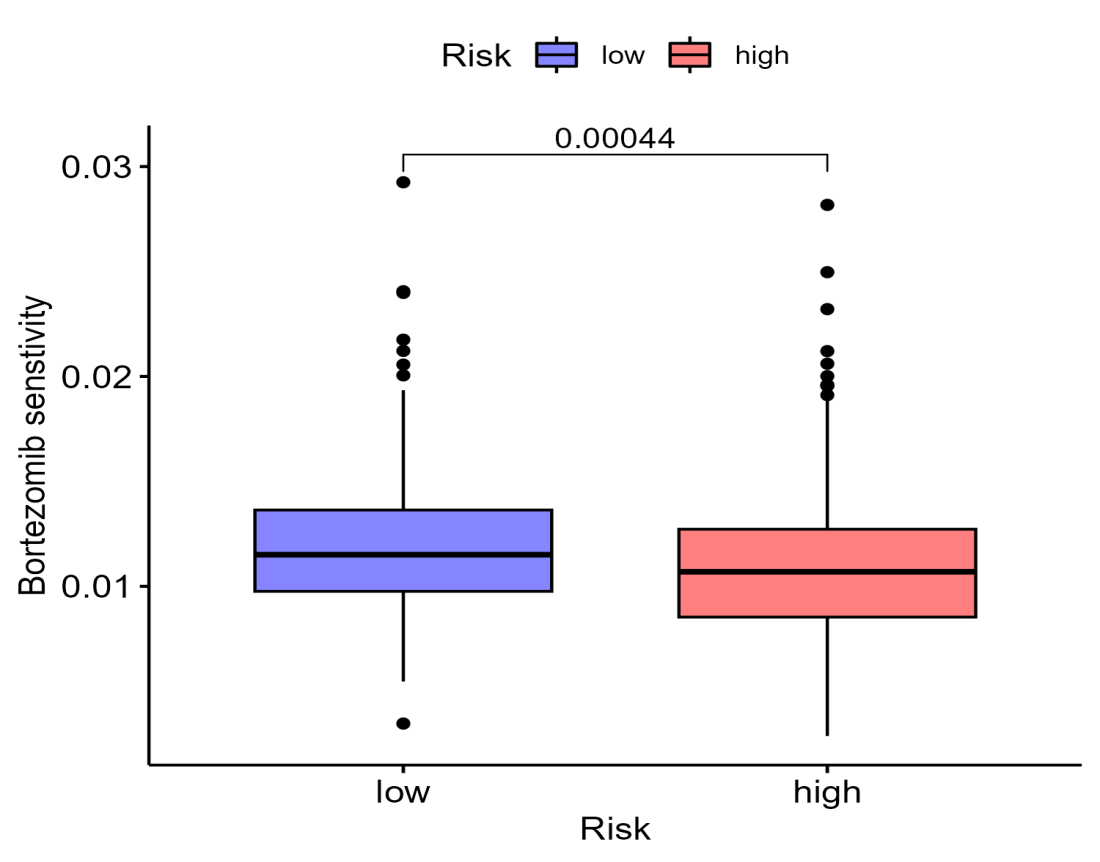


**Figure S11** Significant IC50 difference from Bortezomib between high- and low-risk subgroups


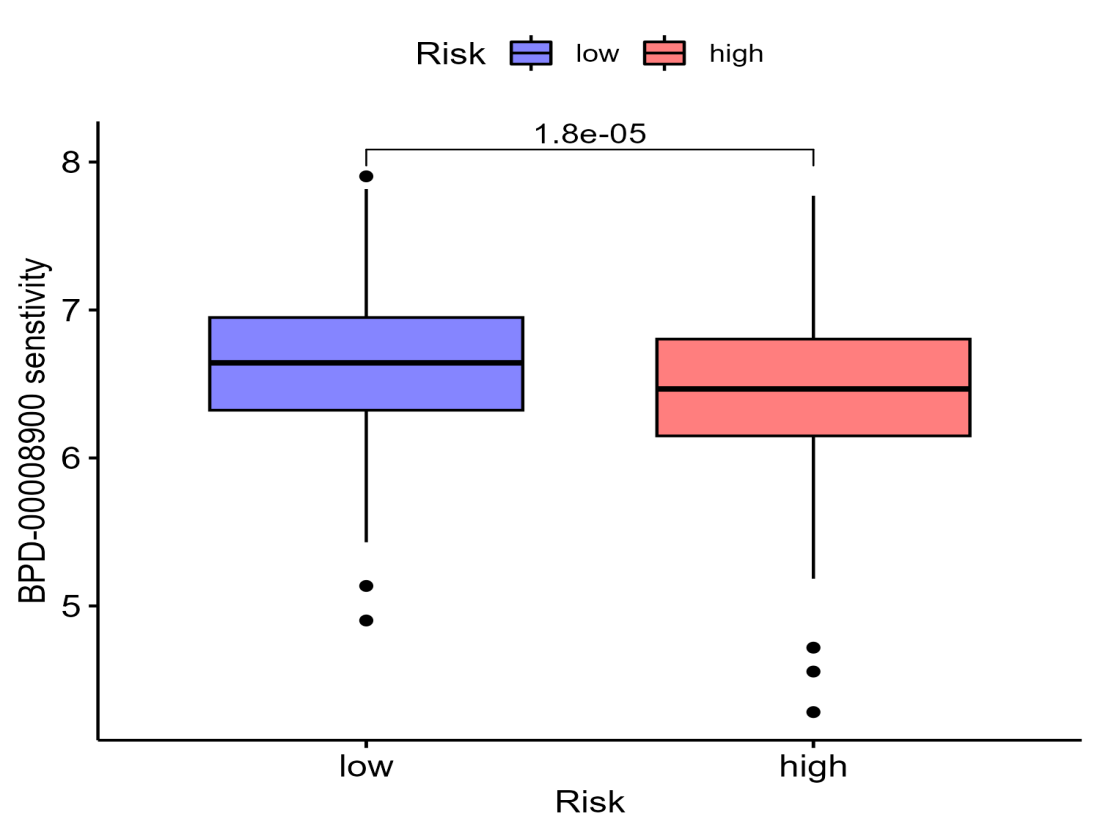


**Figure S12** Significant IC50 difference from BPD-00008900 between high- and low-risk subgroups


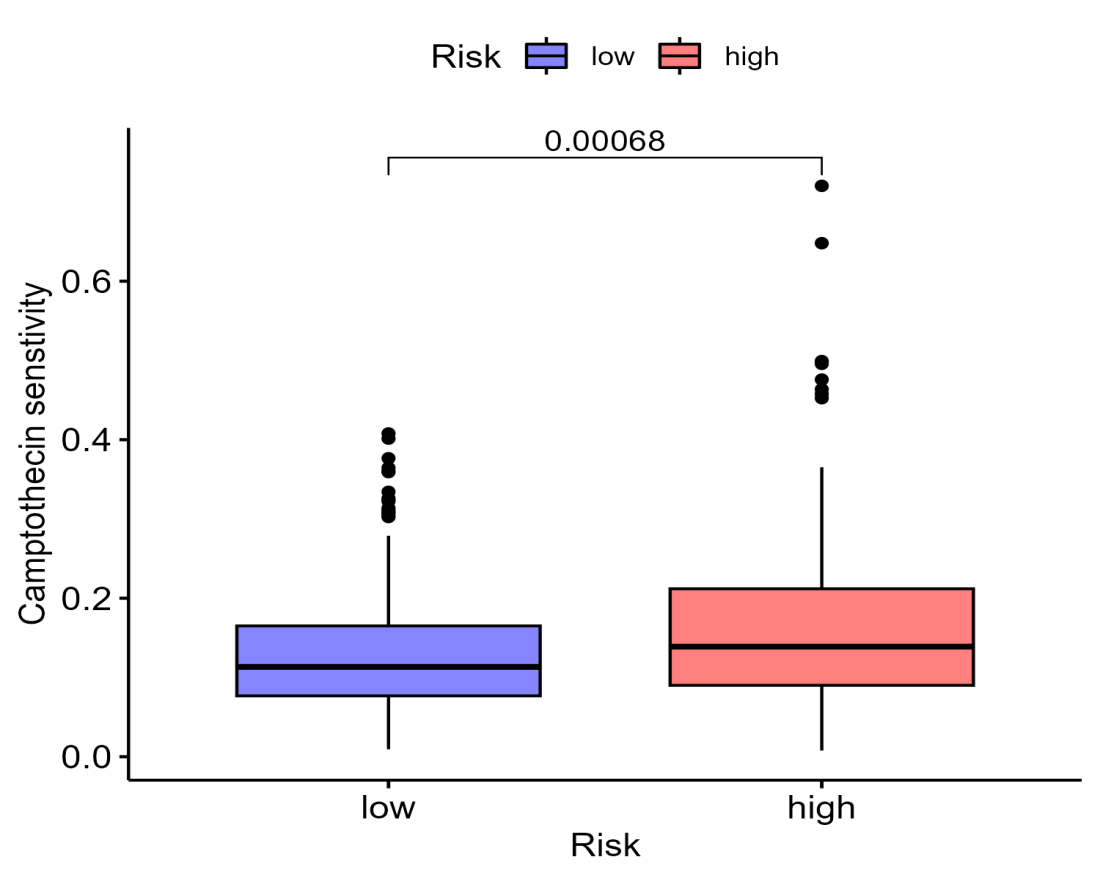


**Figure S13** Significant IC50 difference from Camptothecin between high- and low-risk subgroups


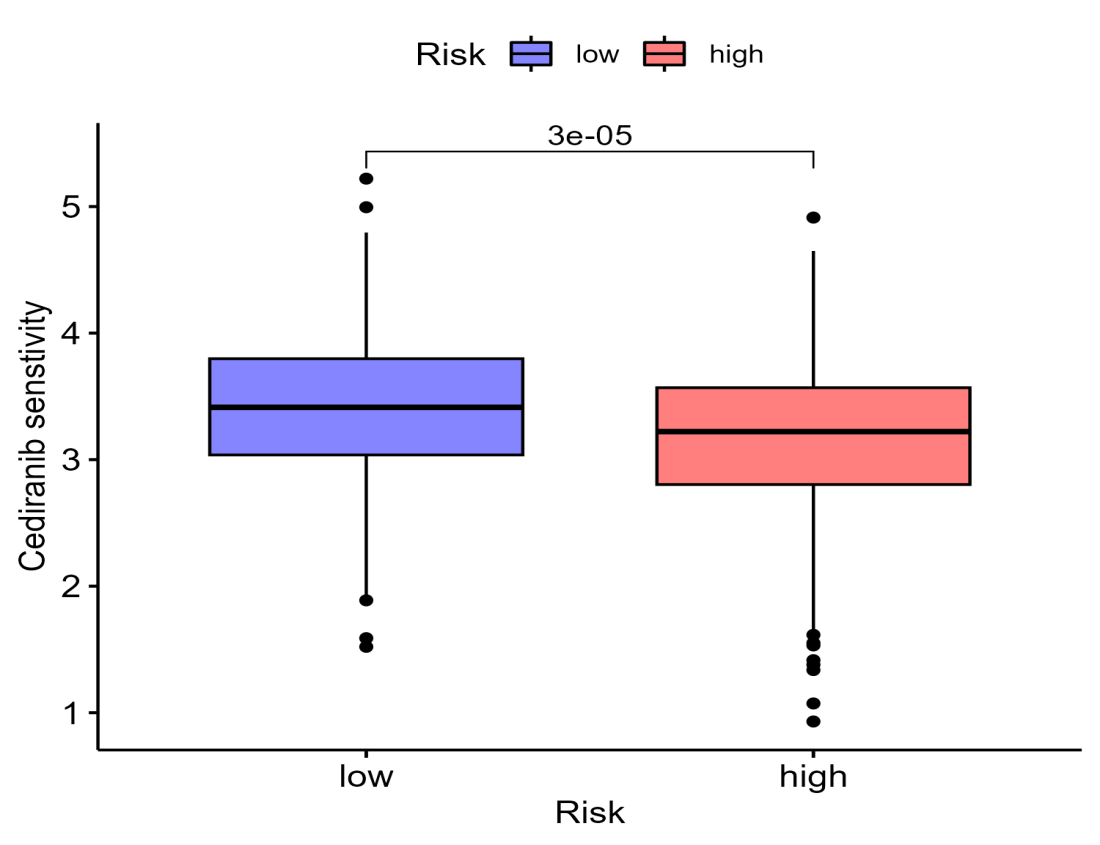


**Figure S14** Significant IC50 difference from Cediranib between high- and low-risk subgroups


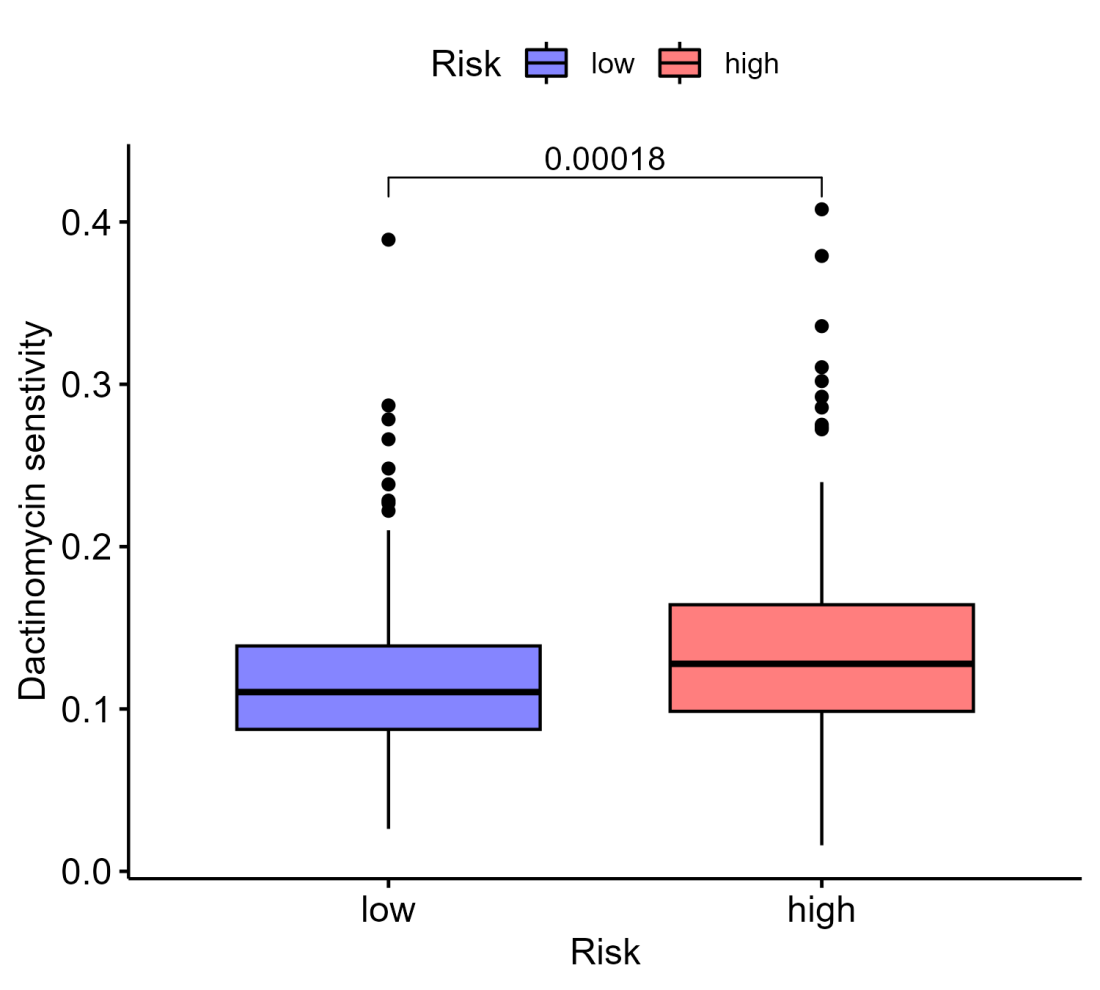


**Figure S15** Significant IC50 difference from Dactinomycin between high- and low-risk subgroups


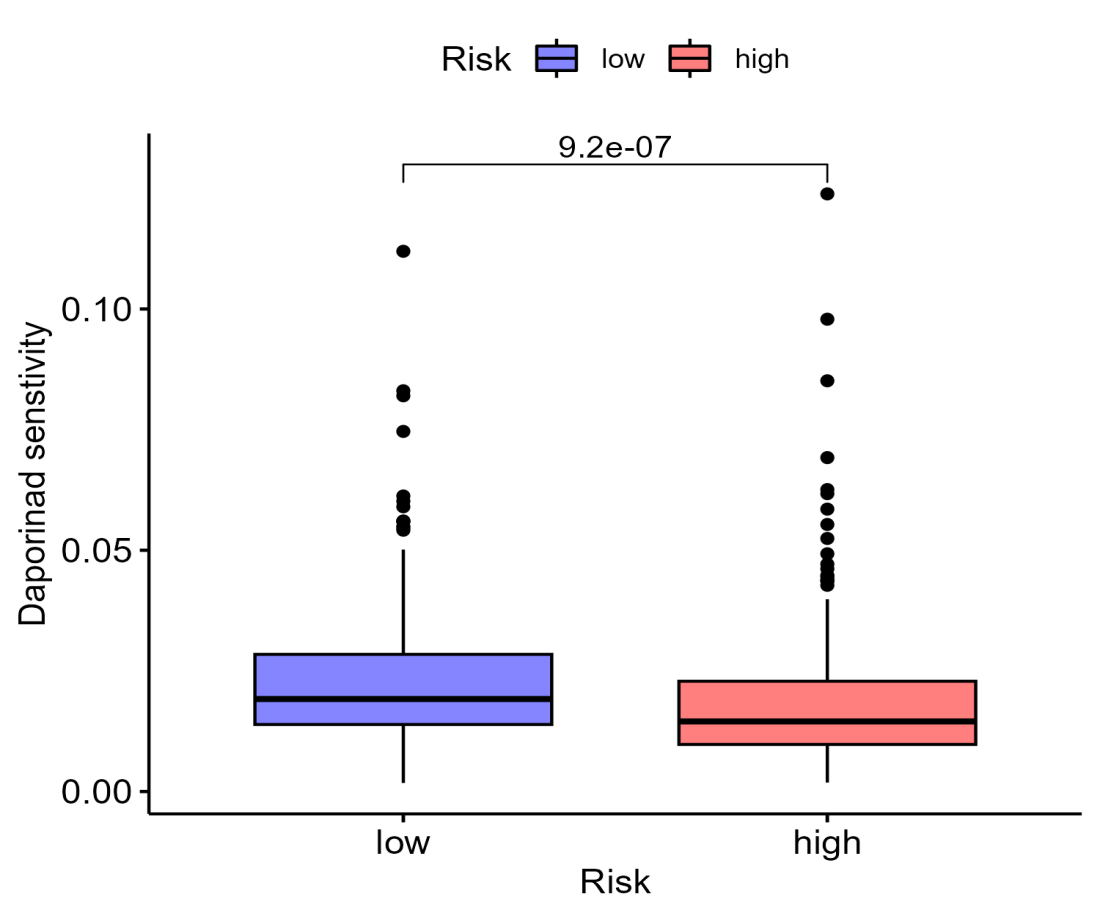


**Figure S16** Significant IC50 difference from Daporinad between high- and low-risk subgroups


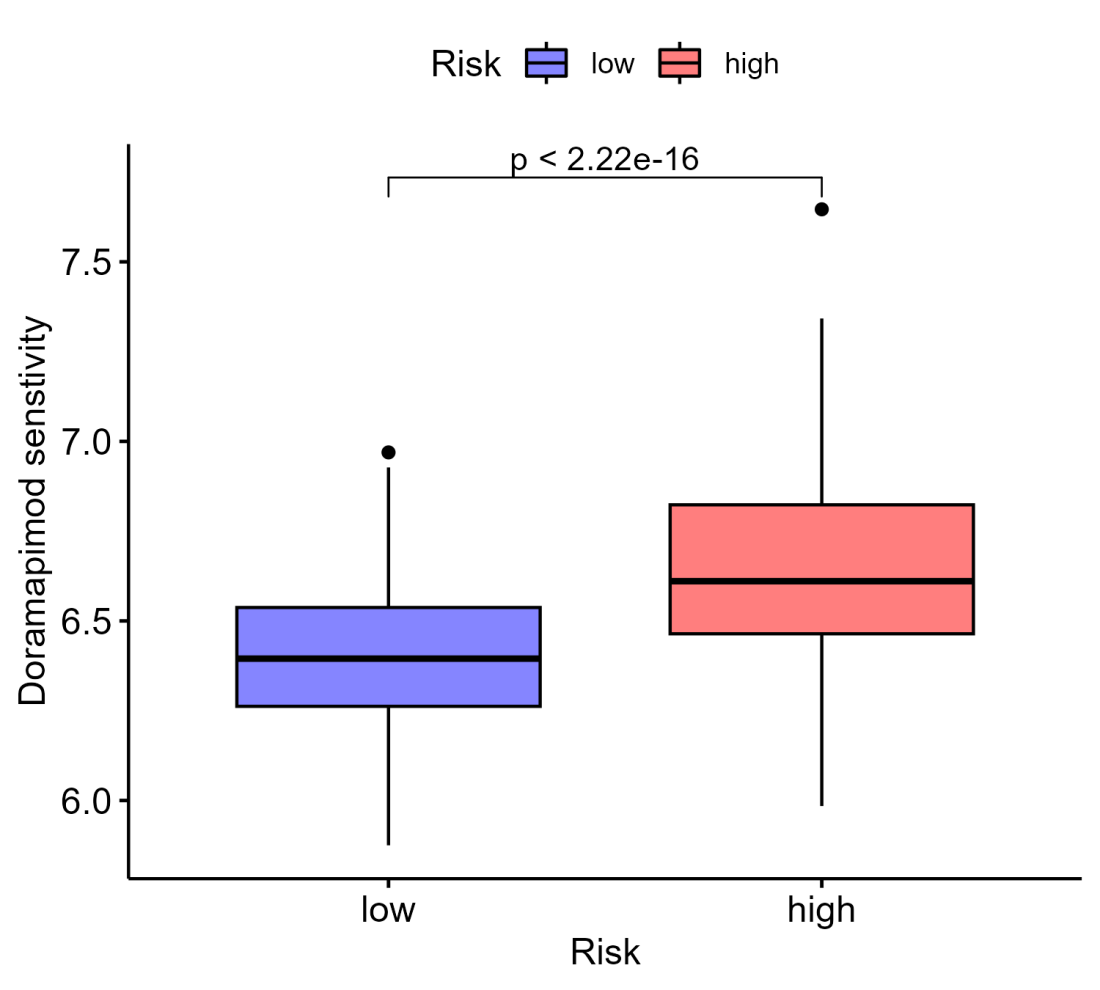


**Figure S17** Significant IC50 difference from Doramapimod between high- and low-risk subgroups


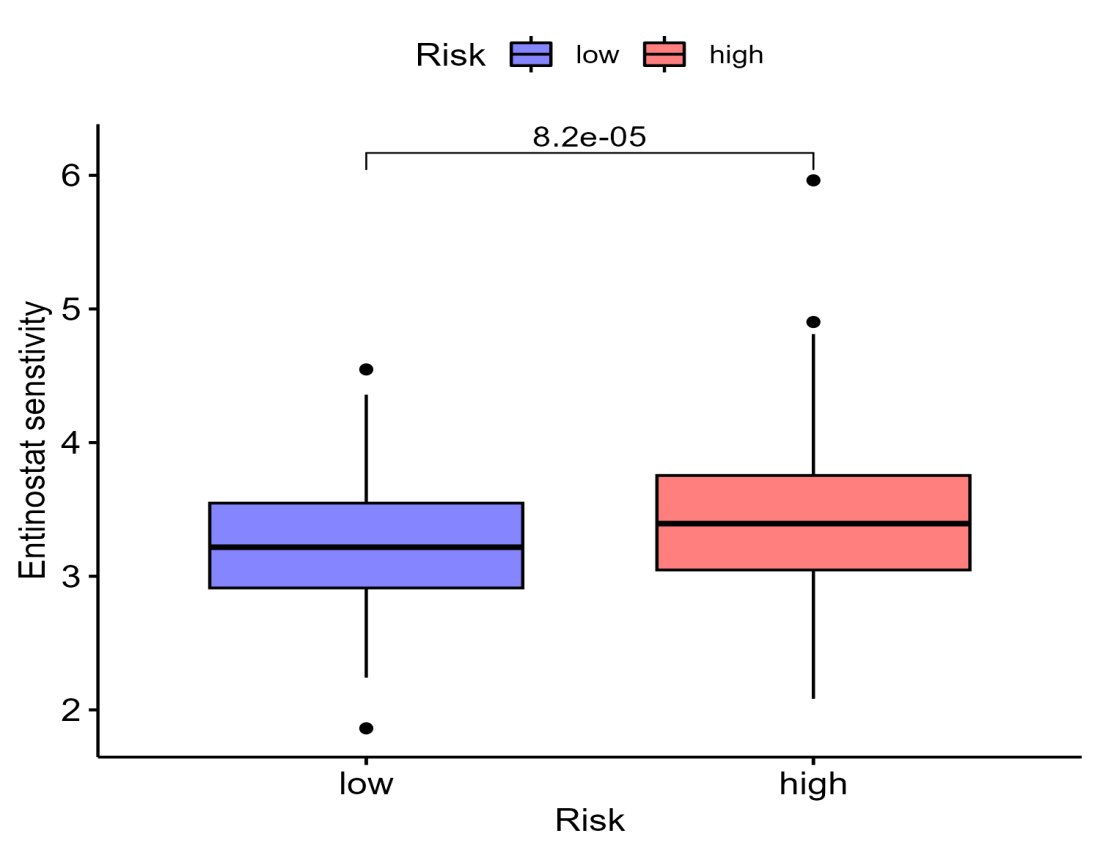


**Figure S18** Significant IC50 difference from Entinostat between high- and low-risk subgroups


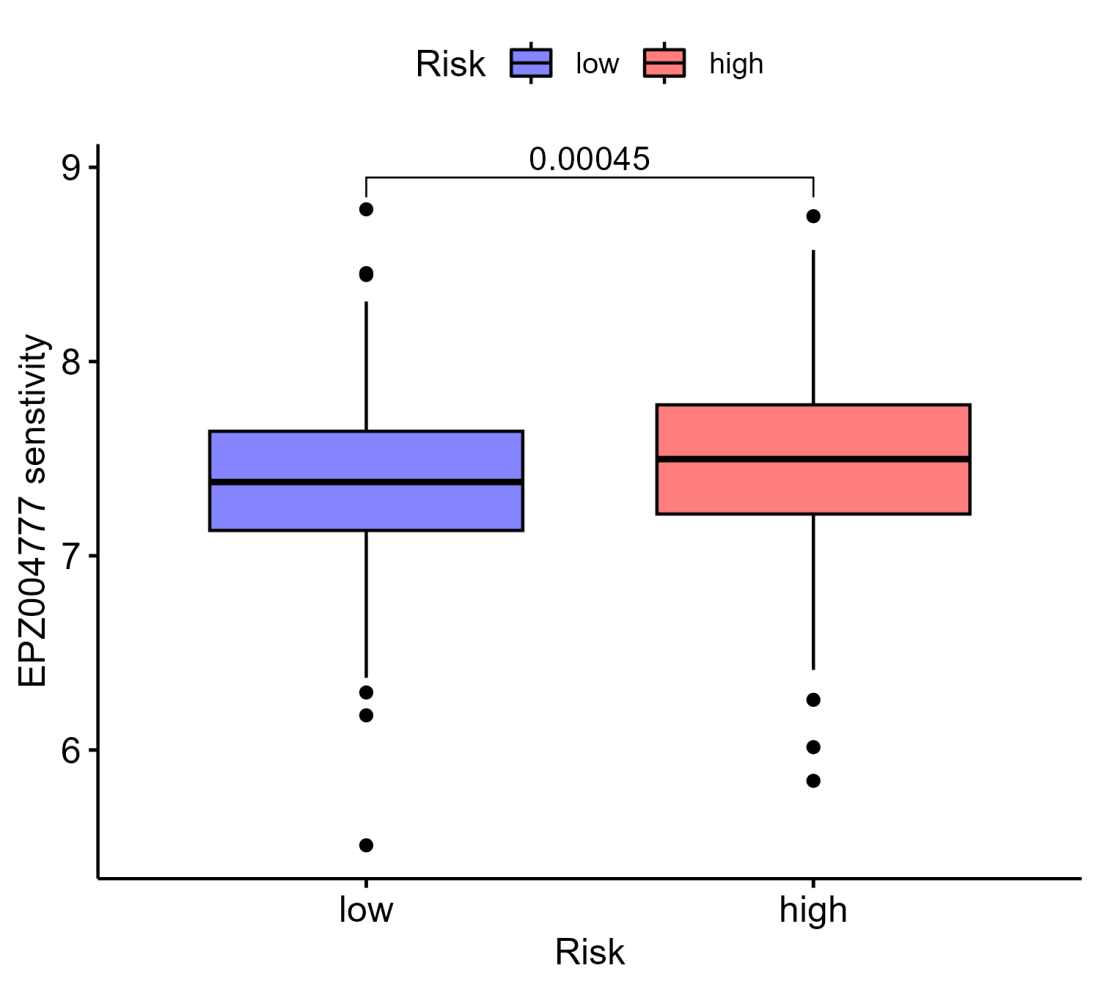


**Figure S19** Significant IC50 difference from EPZ004777 between high- and low-risk subgroups


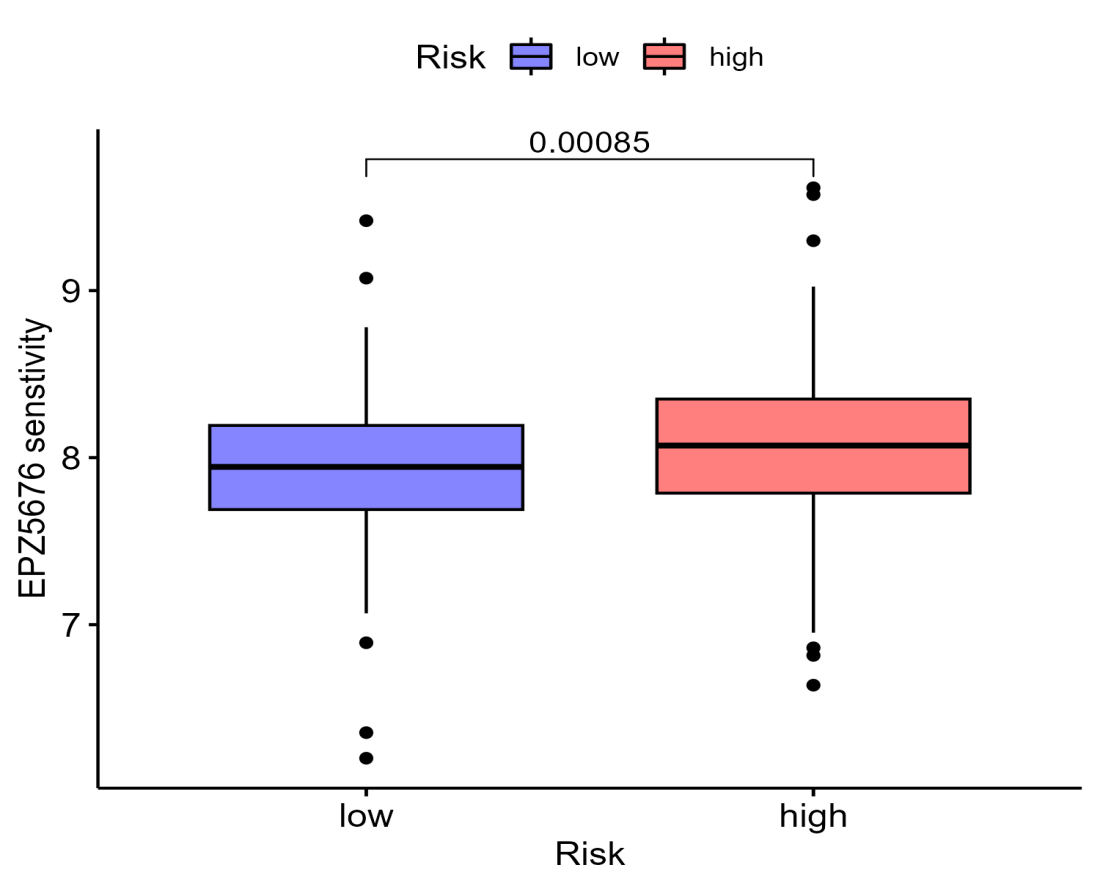


**Figure S20** Significant IC50 difference from EPZ5676 between high- and low-risk subgroups


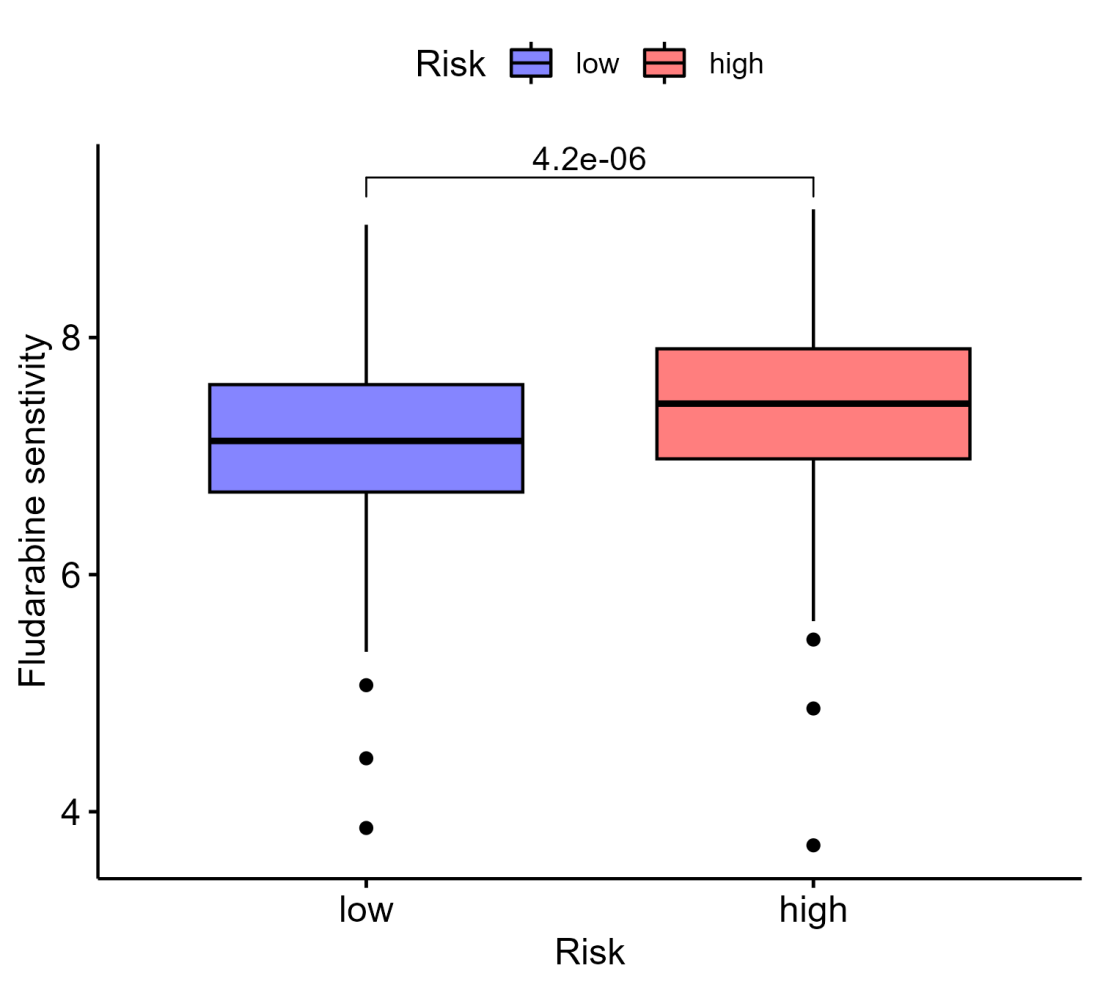


**Figure S21** Significant IC50 difference from Fludarabine between high- and low-risk subgroups


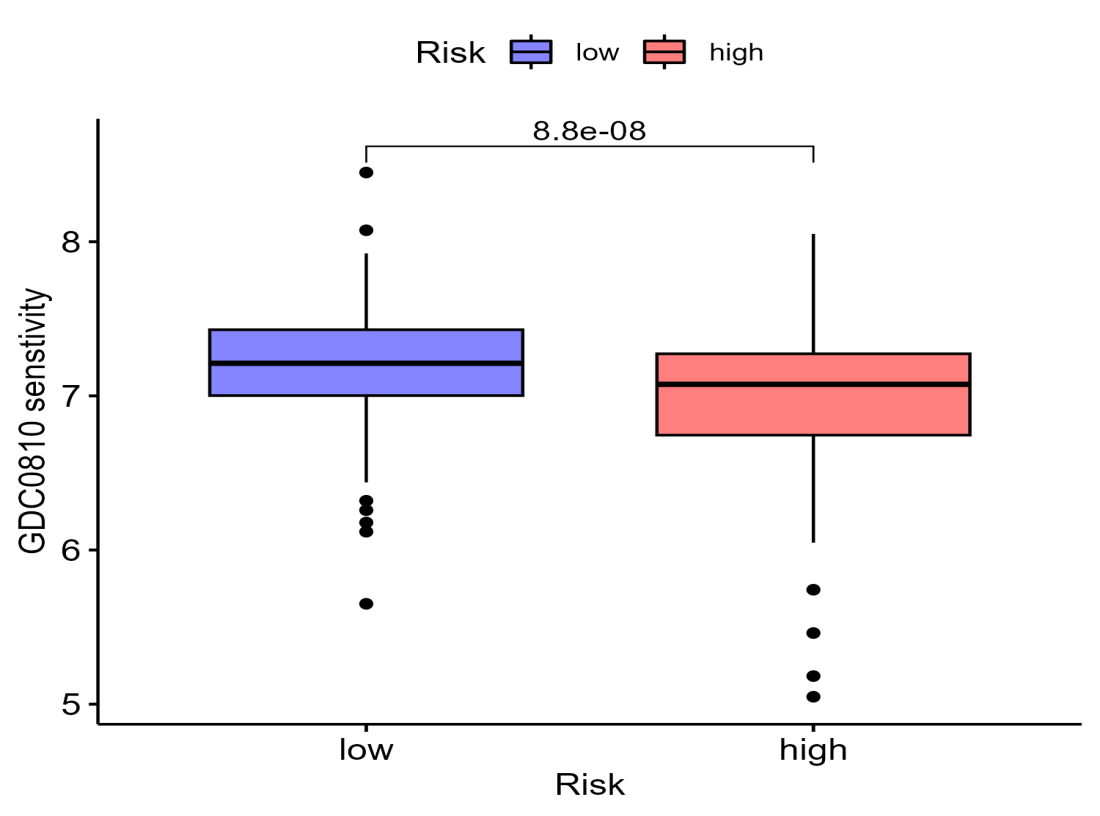


**Figure S22** Significant IC50 difference from GDC0810 between high- and low-risk subgroups


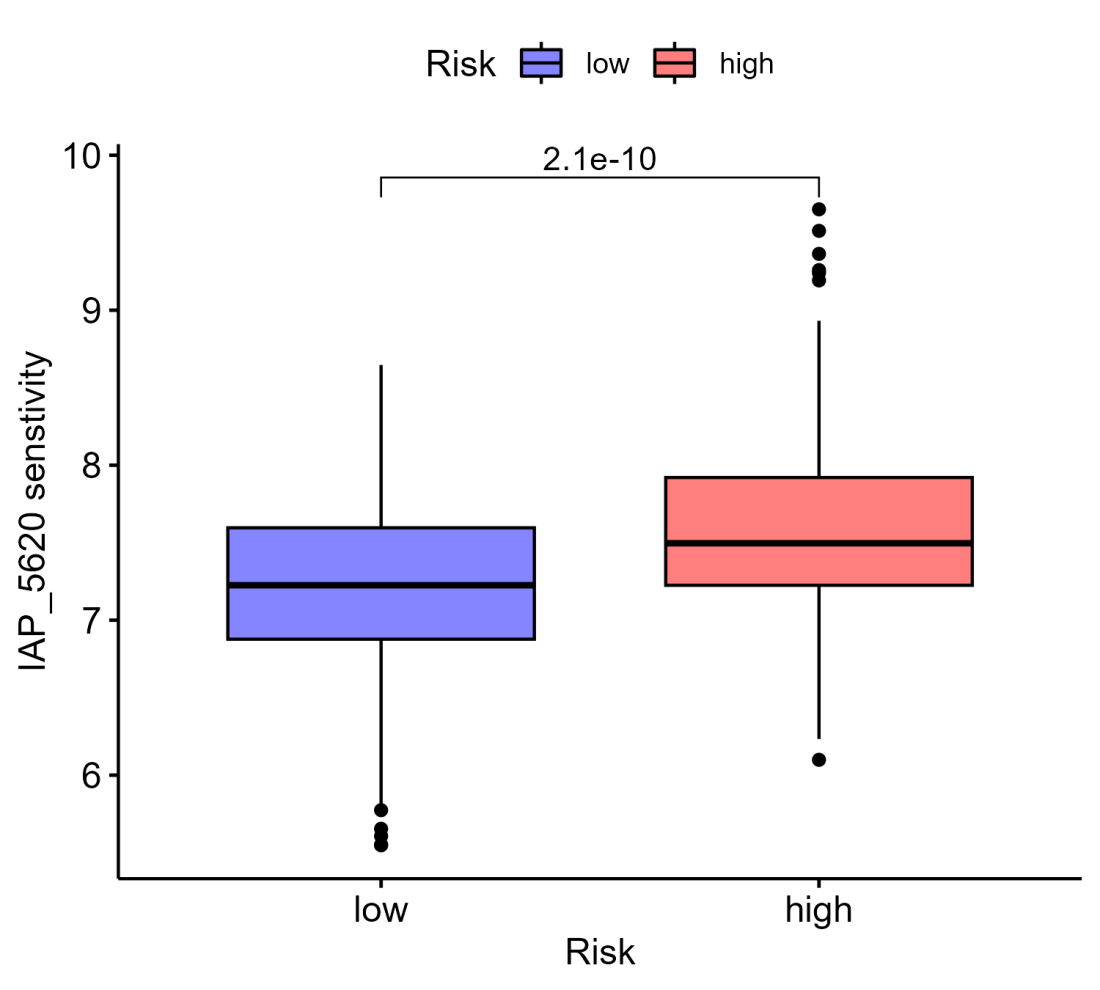


**Figure S23** Significant IC50 difference from IAP_5620 between high- and low-risk subgroups


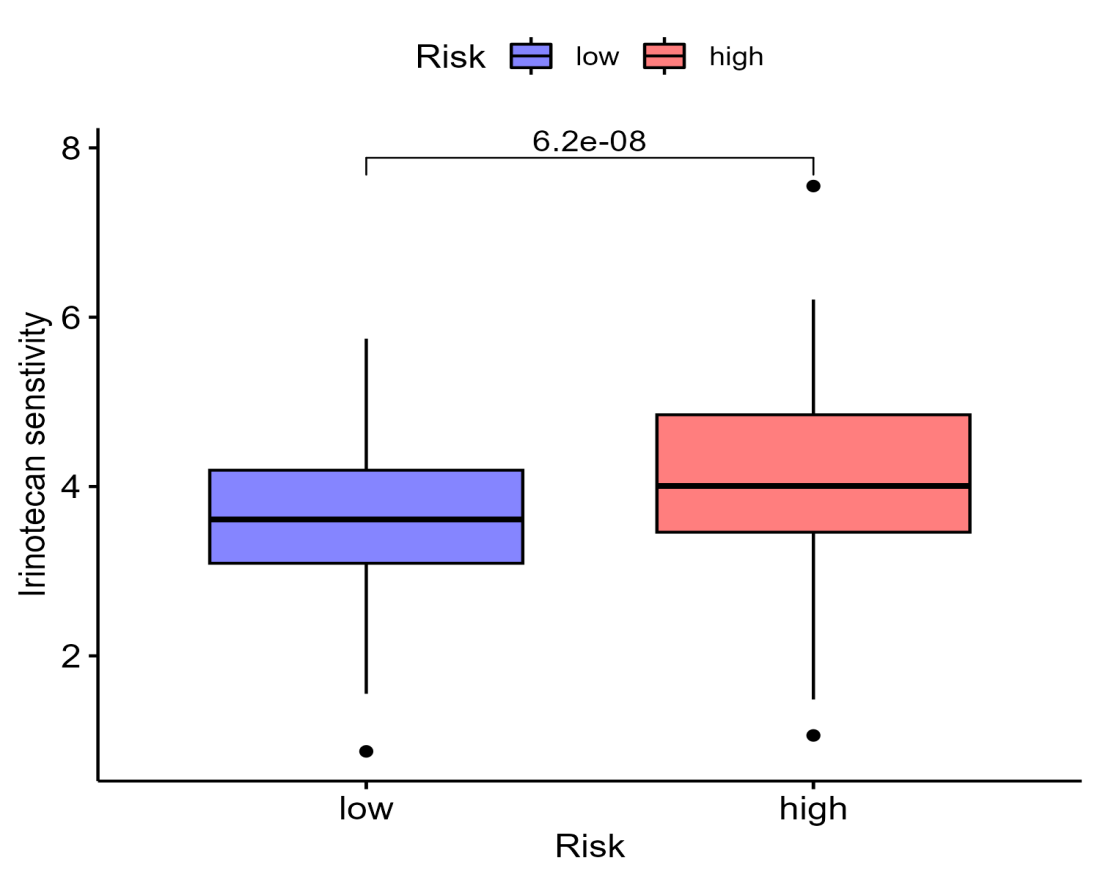


**Figure S24** Significant IC50 difference from Irinotecan between high- and low-risk subgroups


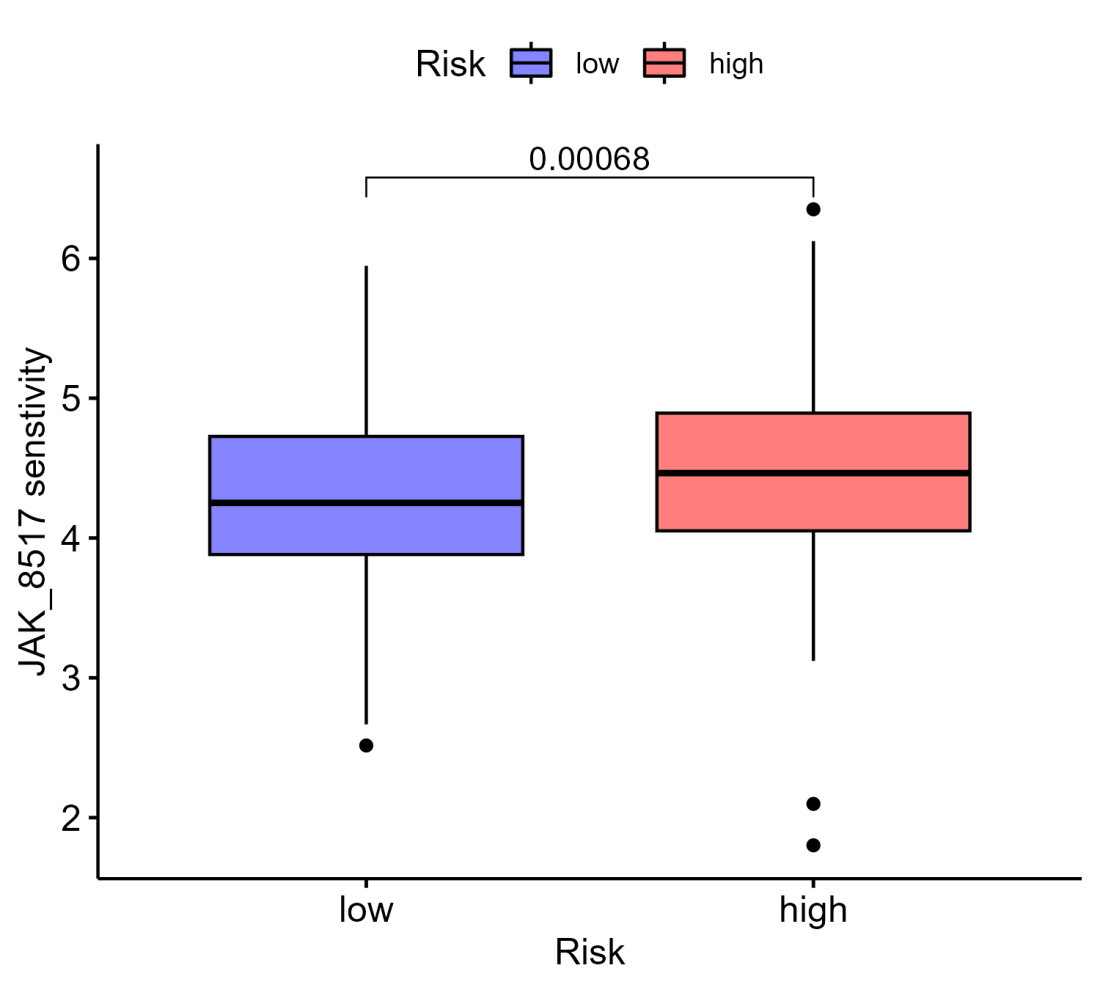


**Figure S25** Significant IC50 difference from JAK_8517 between high- and low-risk subgroups


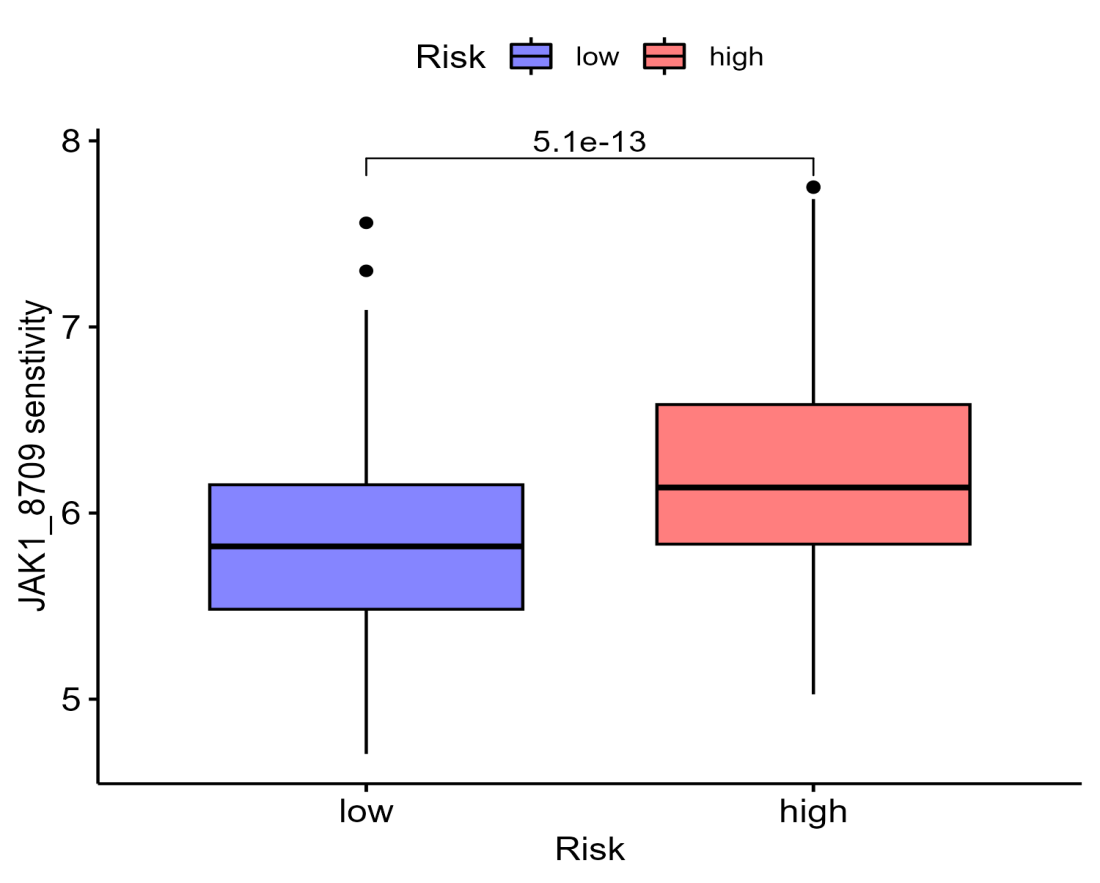


**Figure S26** Significant IC50 difference from JAK1_8709 between high- and low-risk subgroups


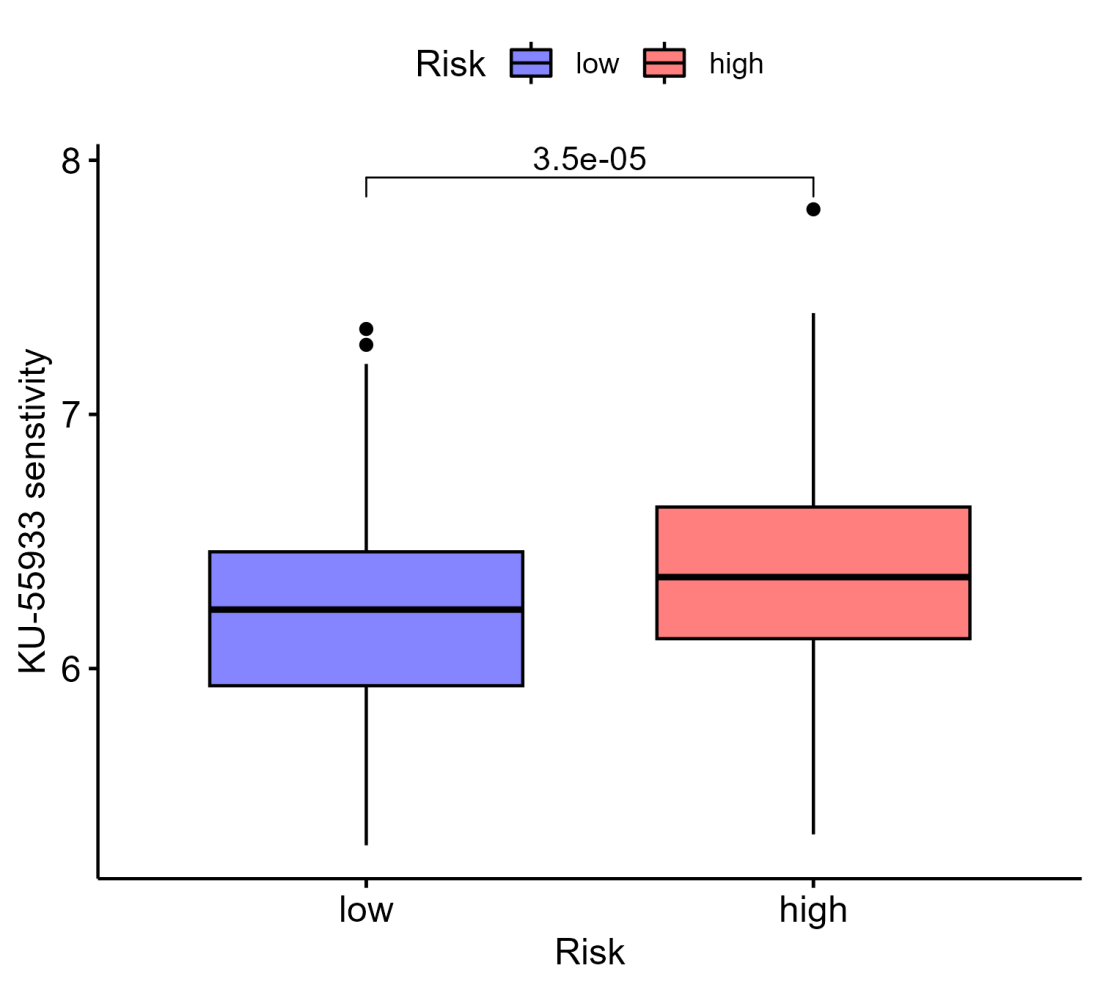


**Figure S27** Significant IC50 difference from KU-55933 between high- and low-risk subgroups


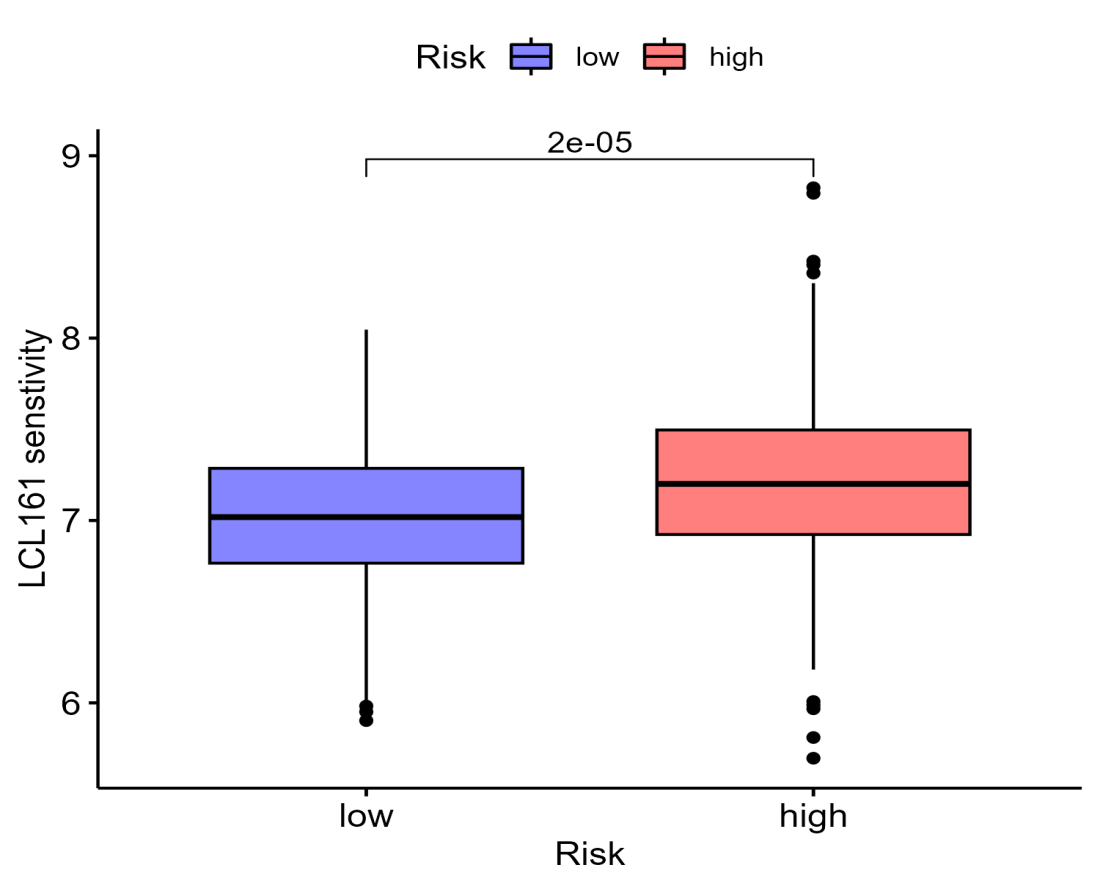


**Figure S28** Significant IC50 difference from LCL161 between high- and low-risk subgroups


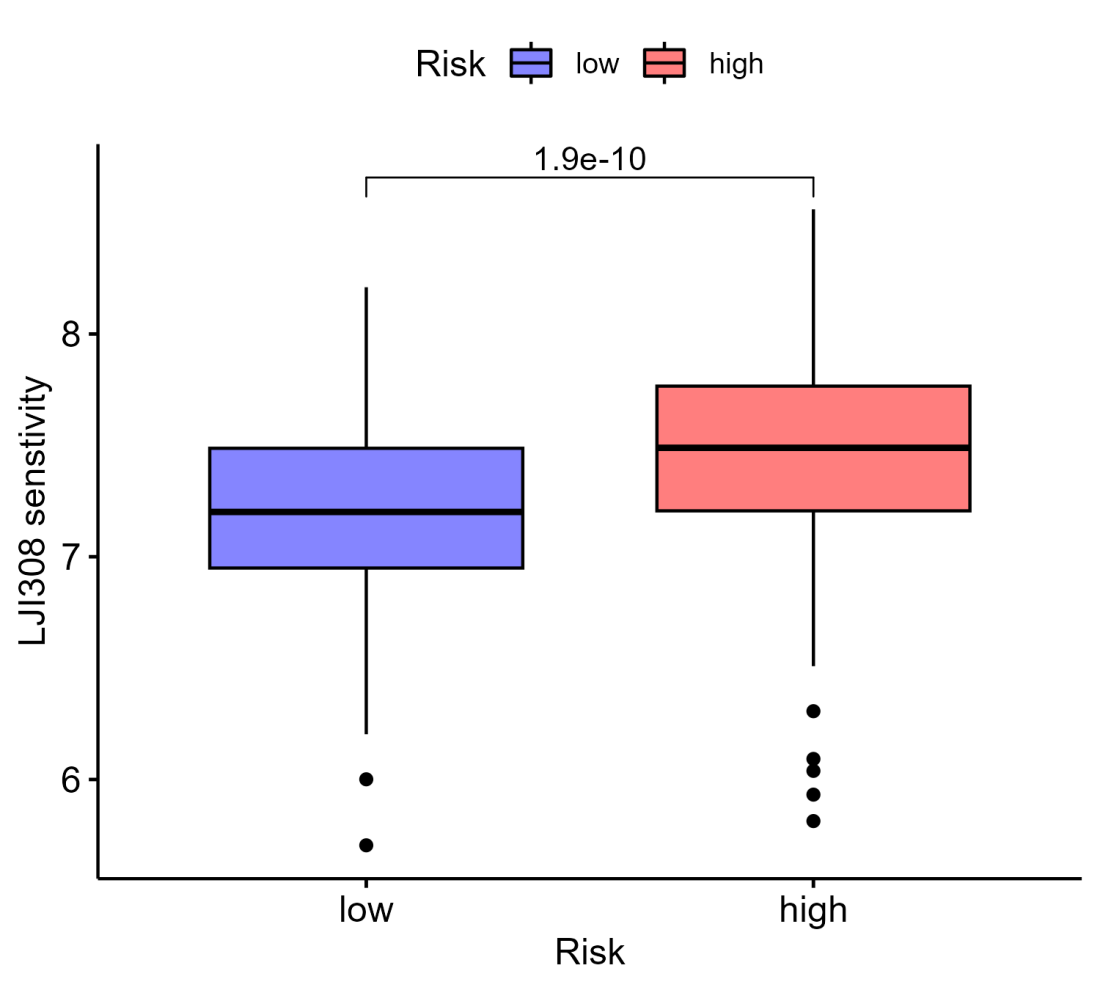


**Figure S29** Significant IC50 difference from LJI308 between high- and low-risk subgroups


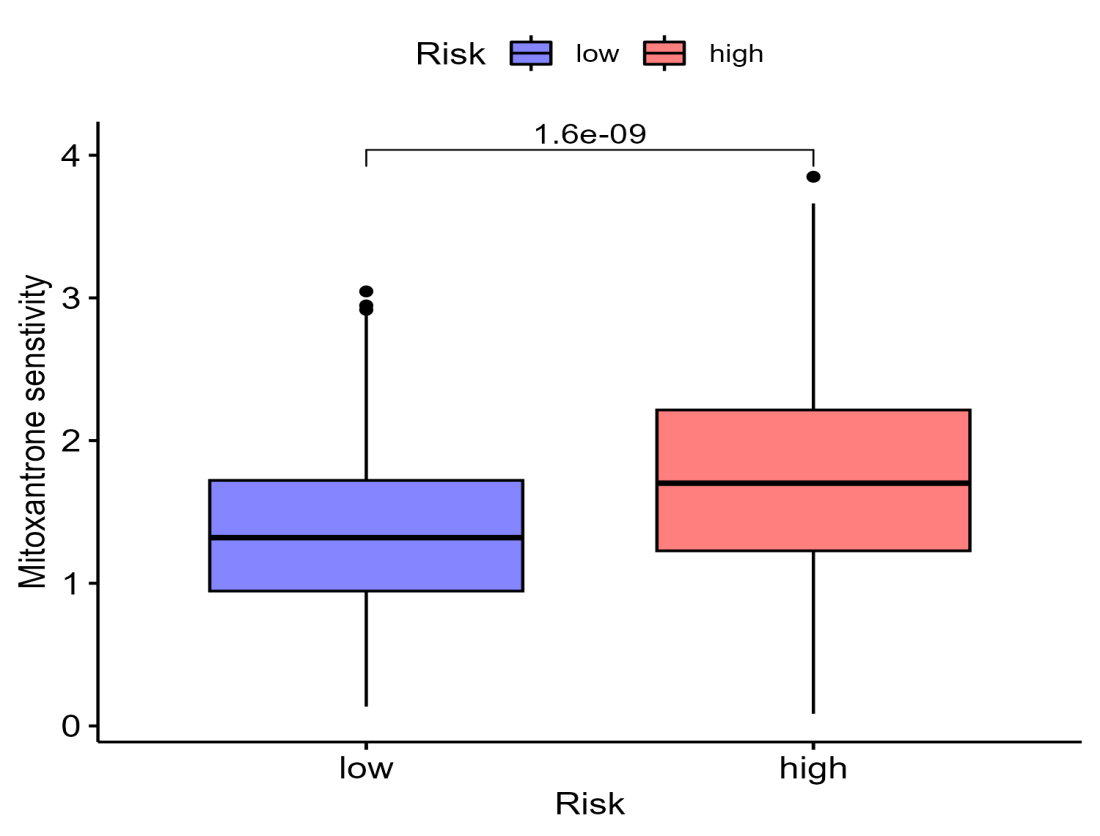


**Figure S30** Significant IC50 difference from Mitoxantrone between high- and low-risk subgroups


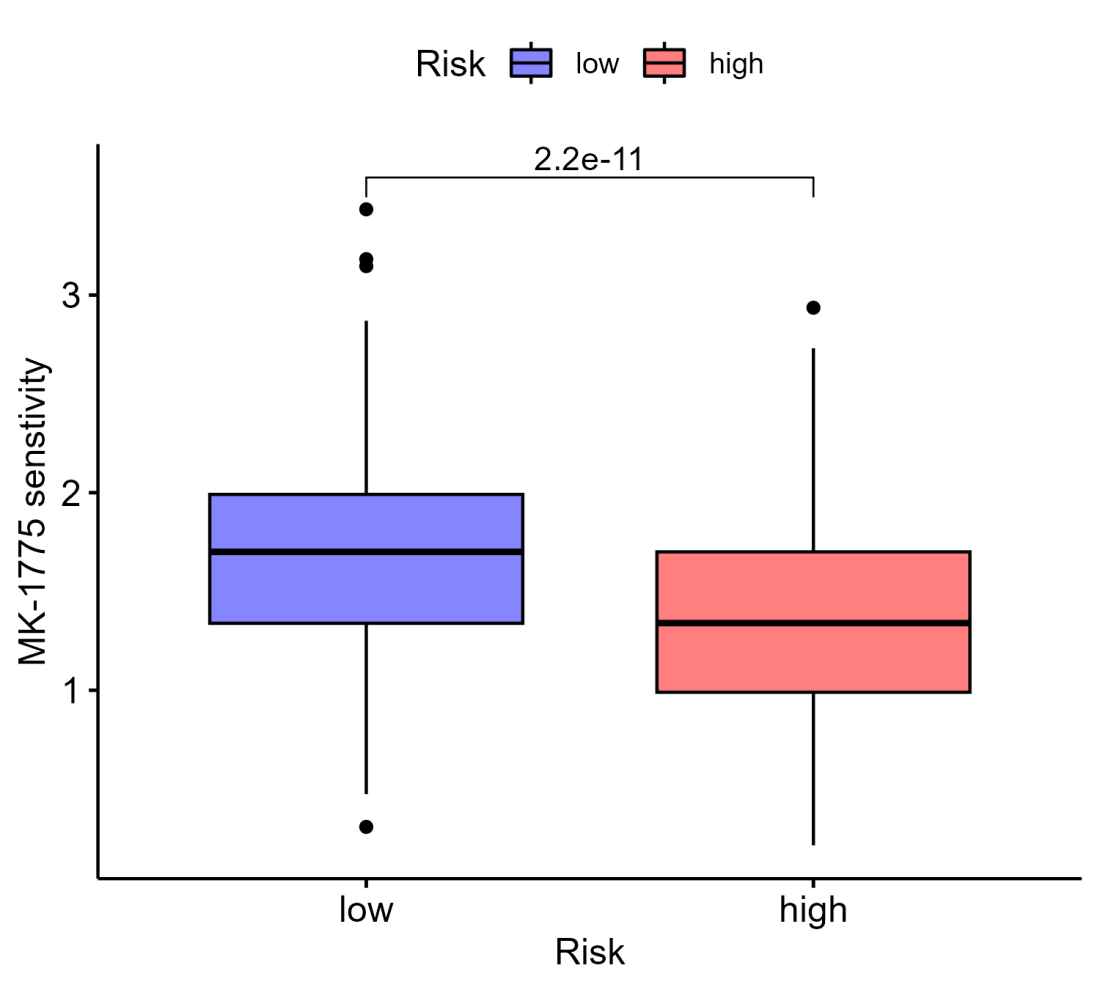


**Figure S31** Significant IC50 difference from MK-1775 between high- and low-risk subgroups


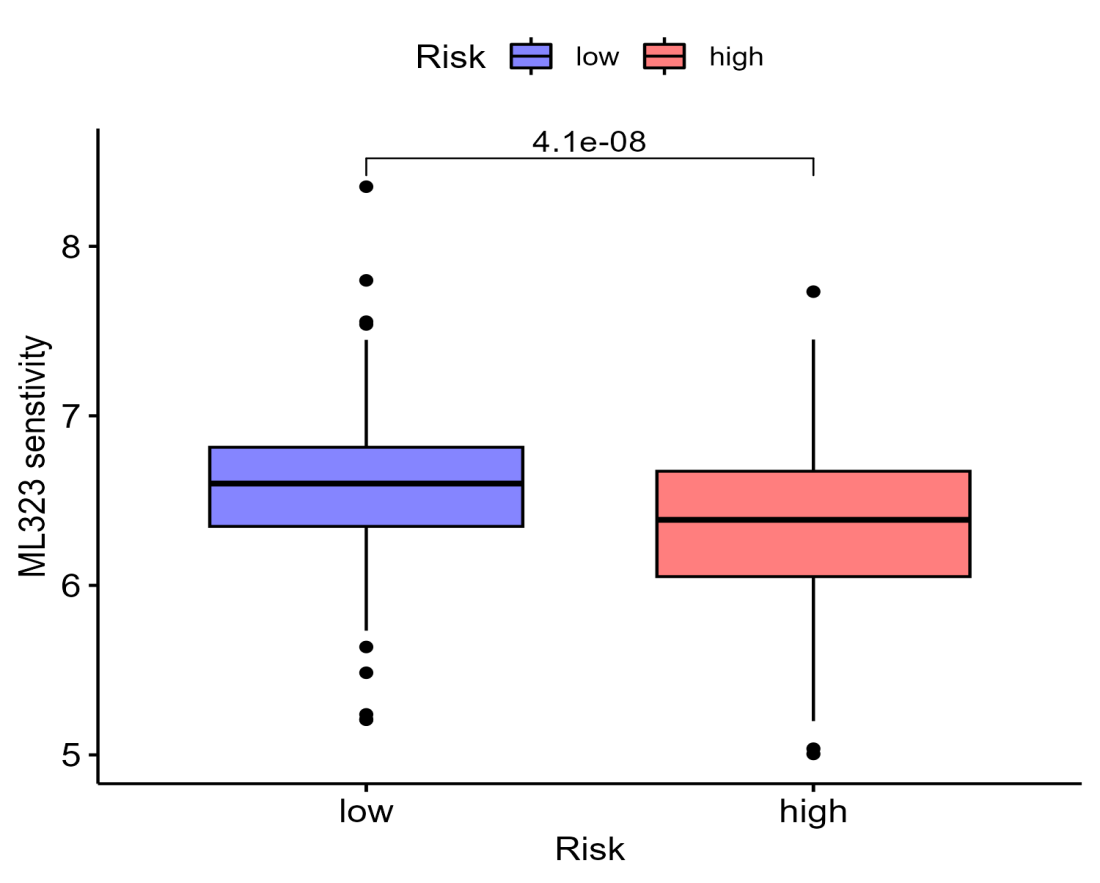


**Figure S32** Significant IC50 difference from ML323 between high- and low-risk subgroups


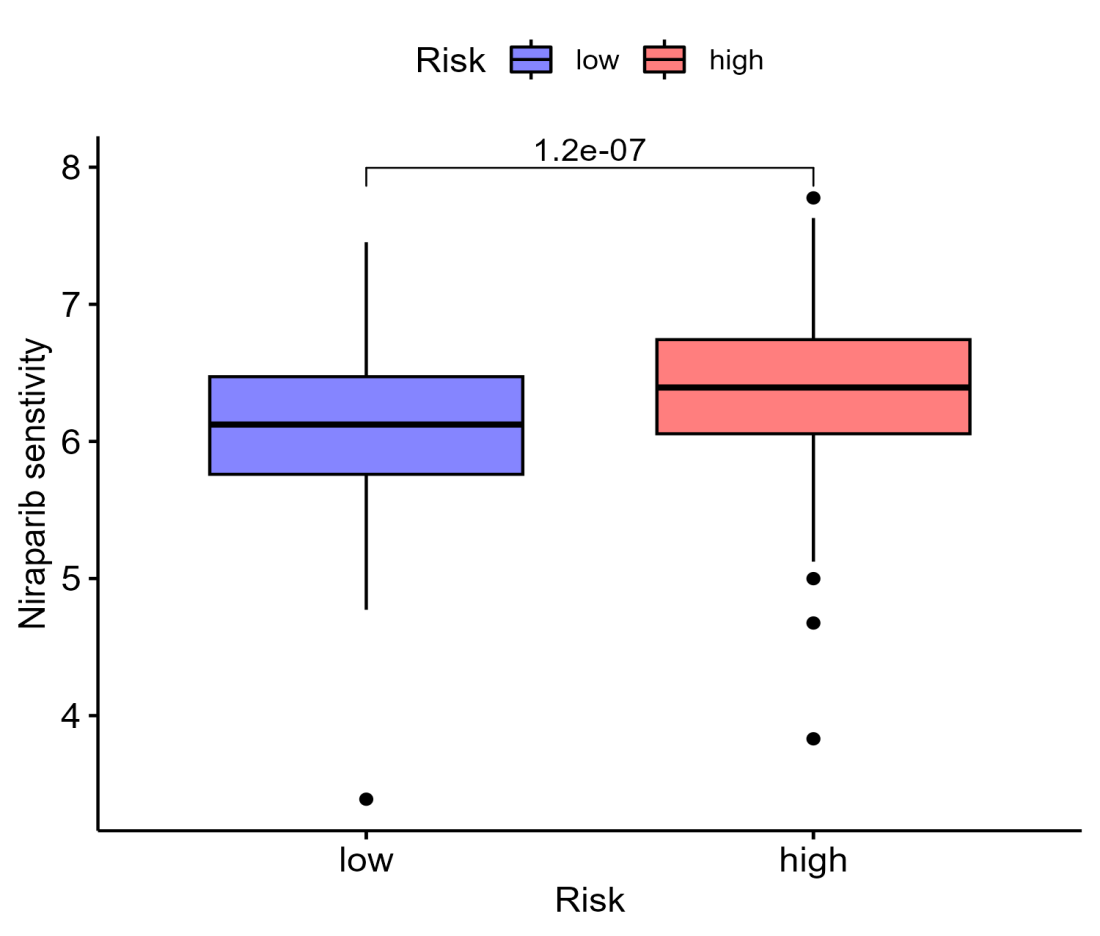


**Figure S33** Significant IC50 difference from Niraparib between high- and low-risk subgroups


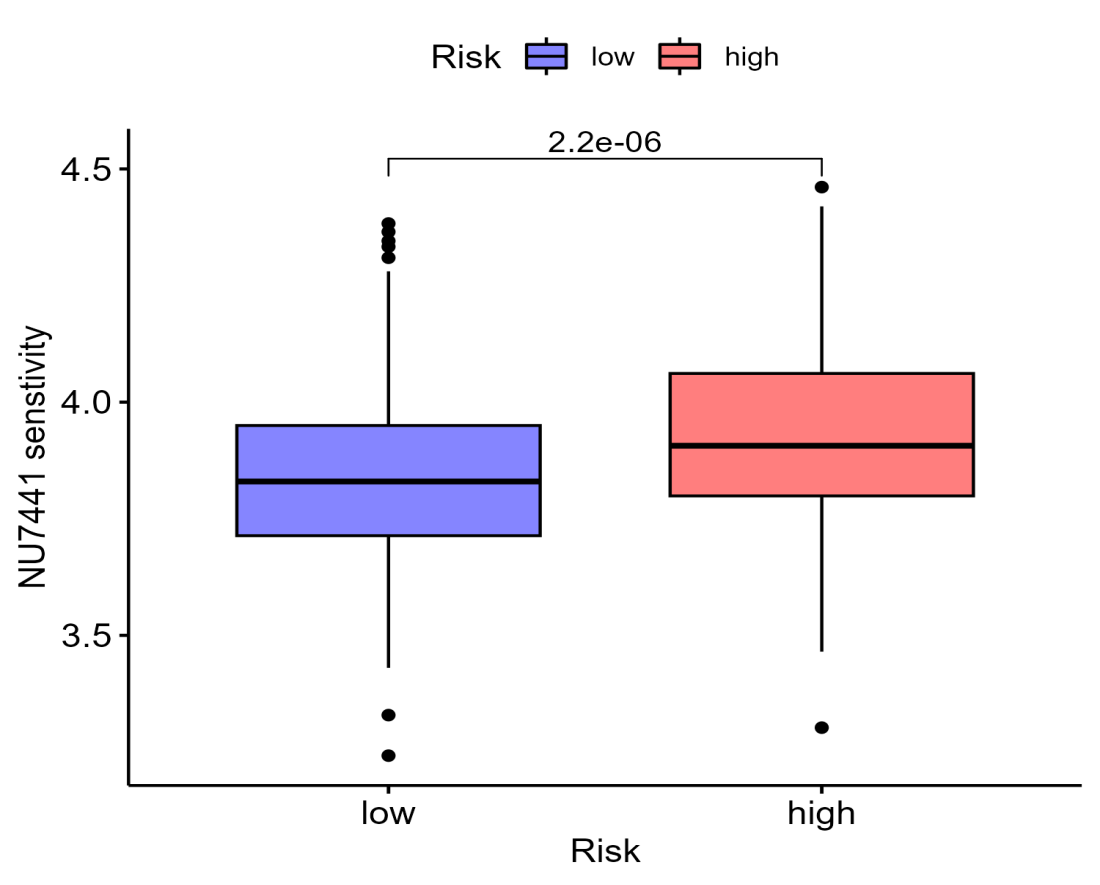


**Figure S34** Significant IC50 difference from NU7441 between high- and low-risk subgroups


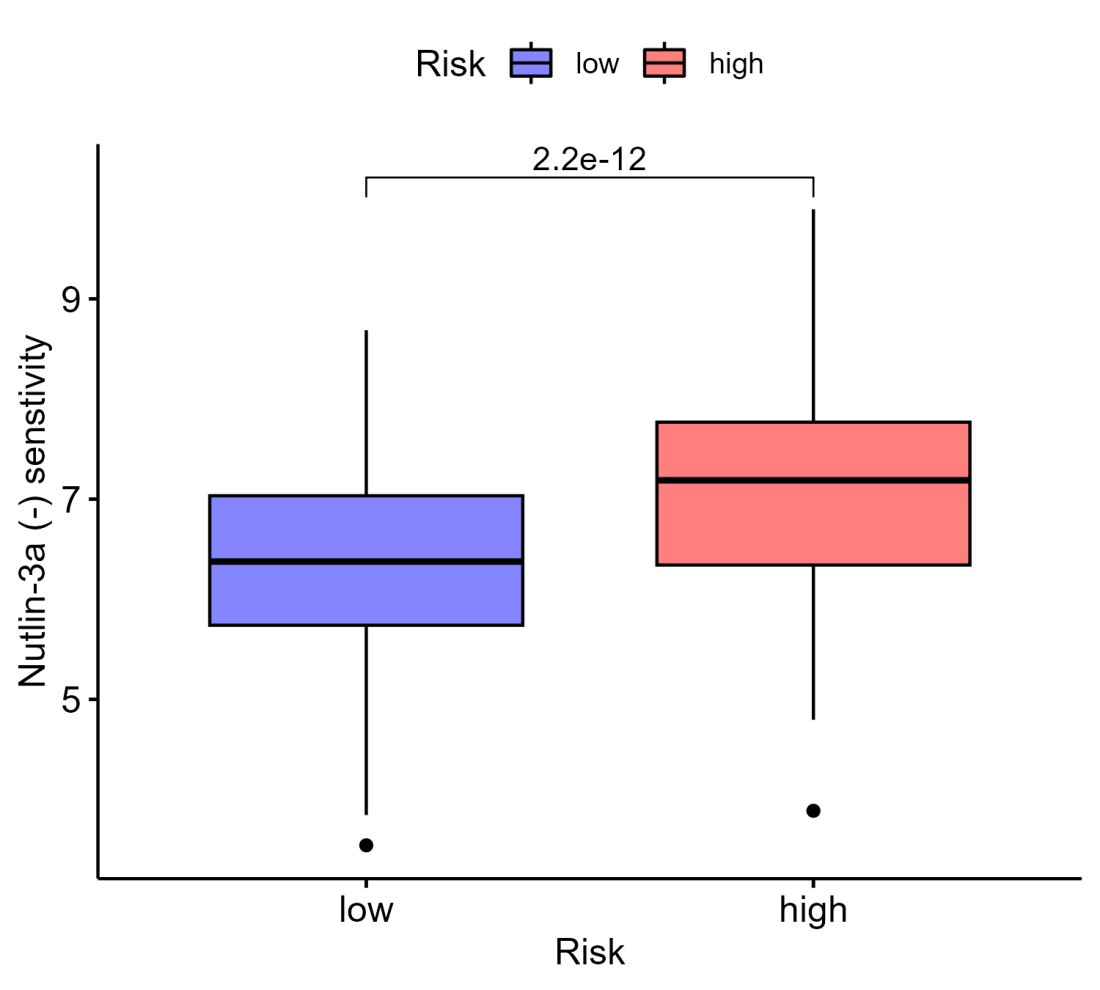


**Figure S35** Significant IC50 difference from Nutlin-3a (-) between high- and low-risk subgroups


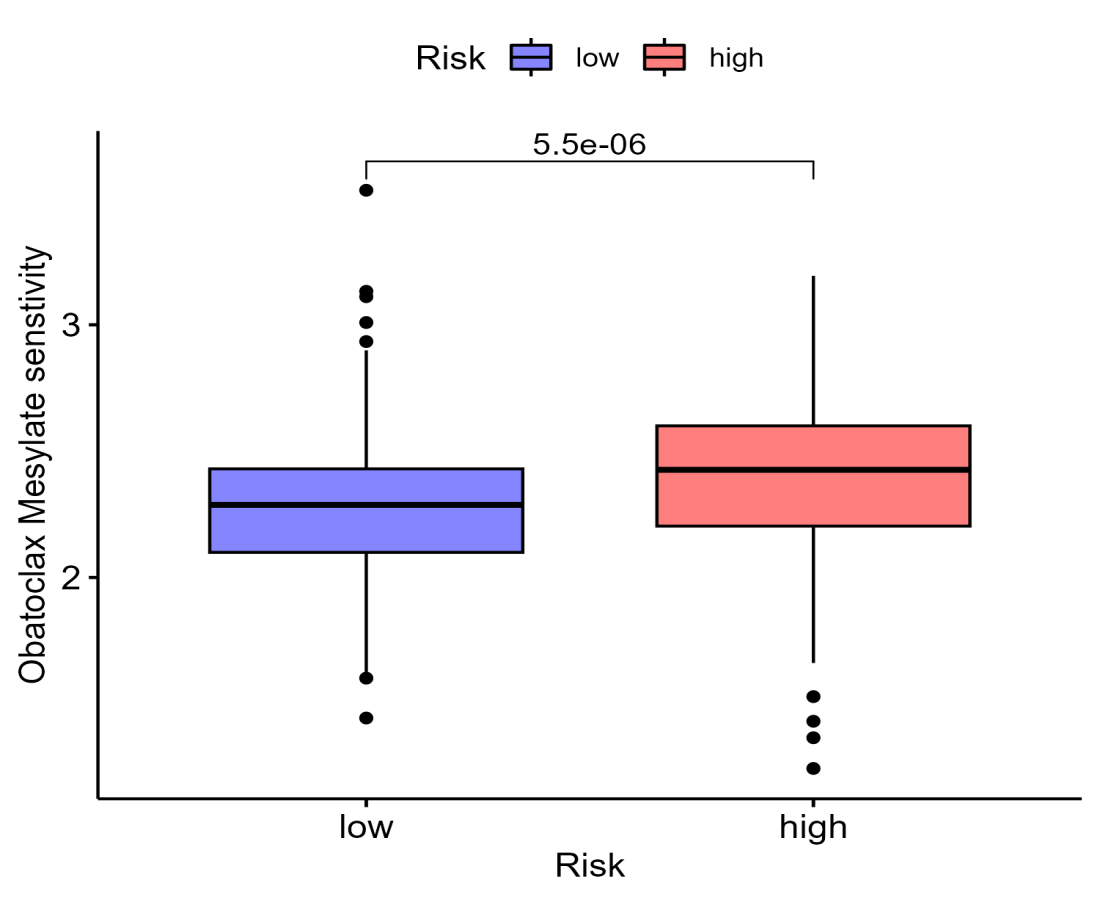


**Figure S36** Significant IC50 difference from Obatoclax Mesylate between high- and low-risk subgroups


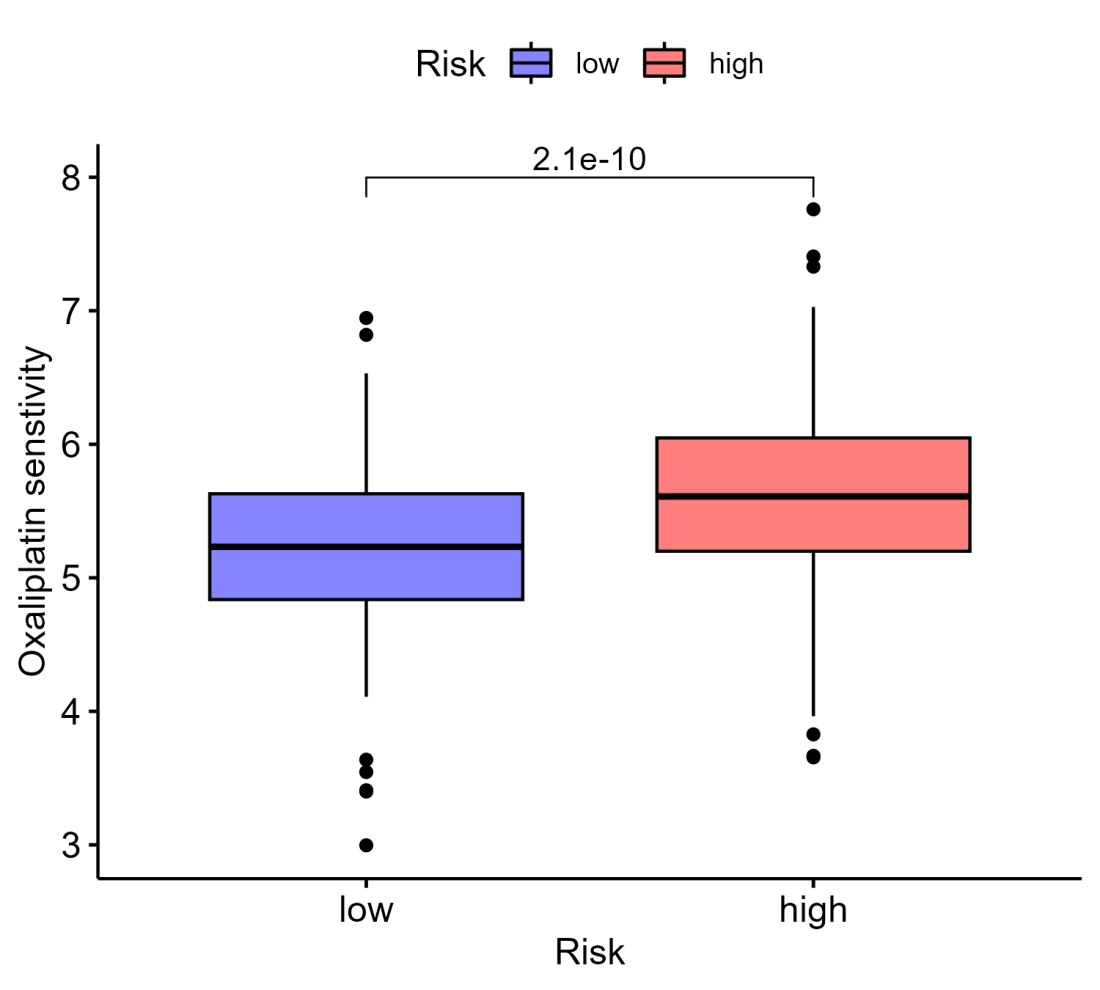


**Figure S37** Significant IC50 difference from Oxaliplatin between high- and low-risk subgroups


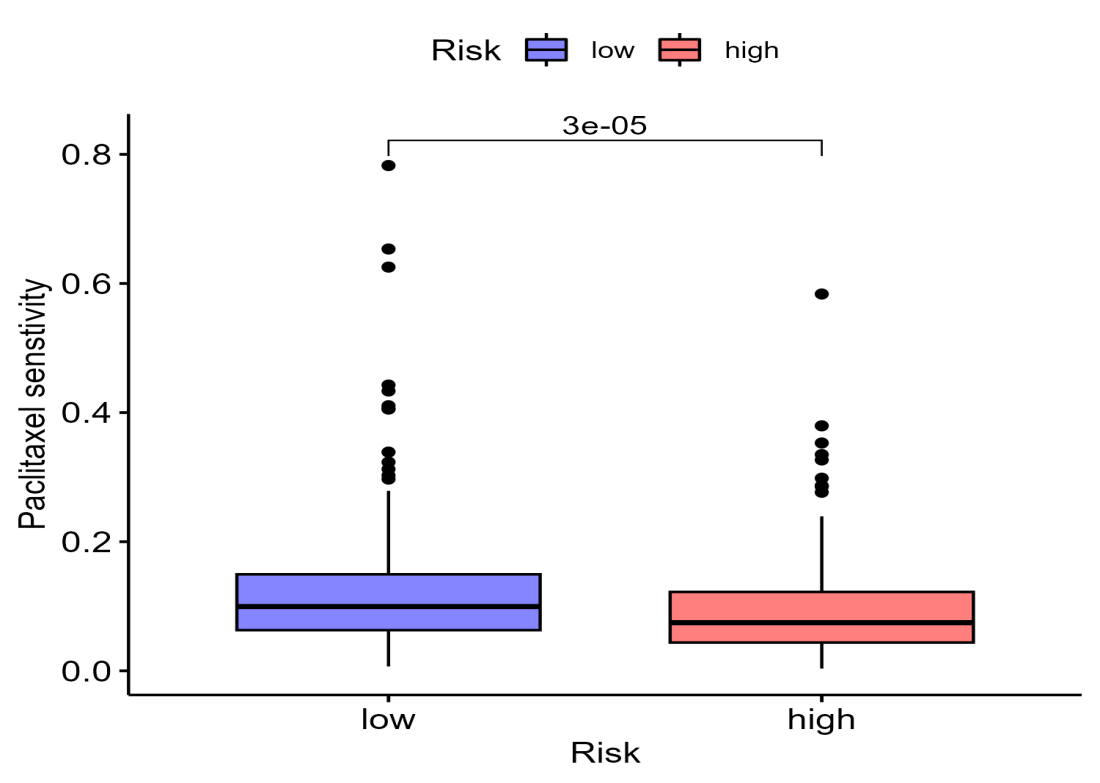


**Figure S38** Significant IC50 difference from Paclitaxel between high- and low-risk subgroups


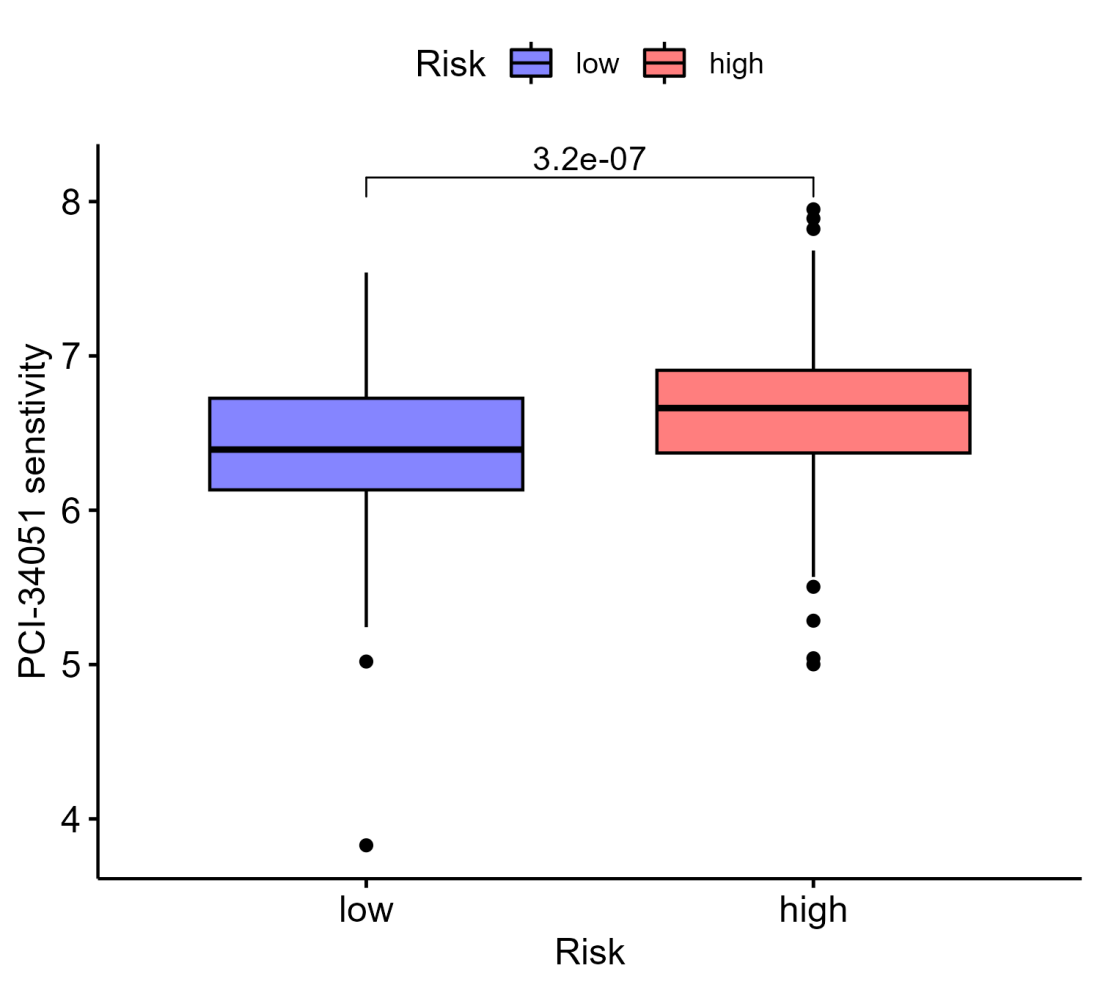


**Figure S39** Significant IC50 difference from PCI-34051 between high- and low-risk subgroups


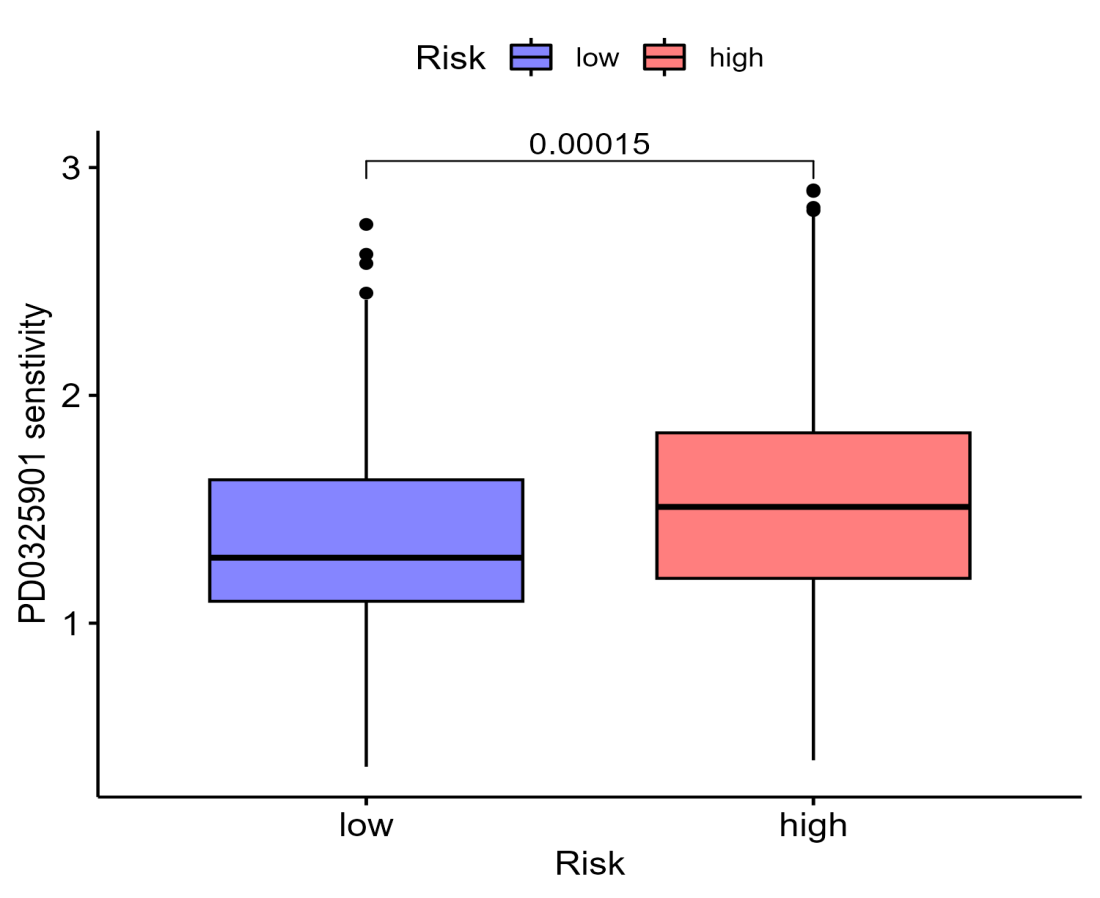


**Figure S40** Significant IC50 difference from PD0325901 between high- and low-risk subgroups


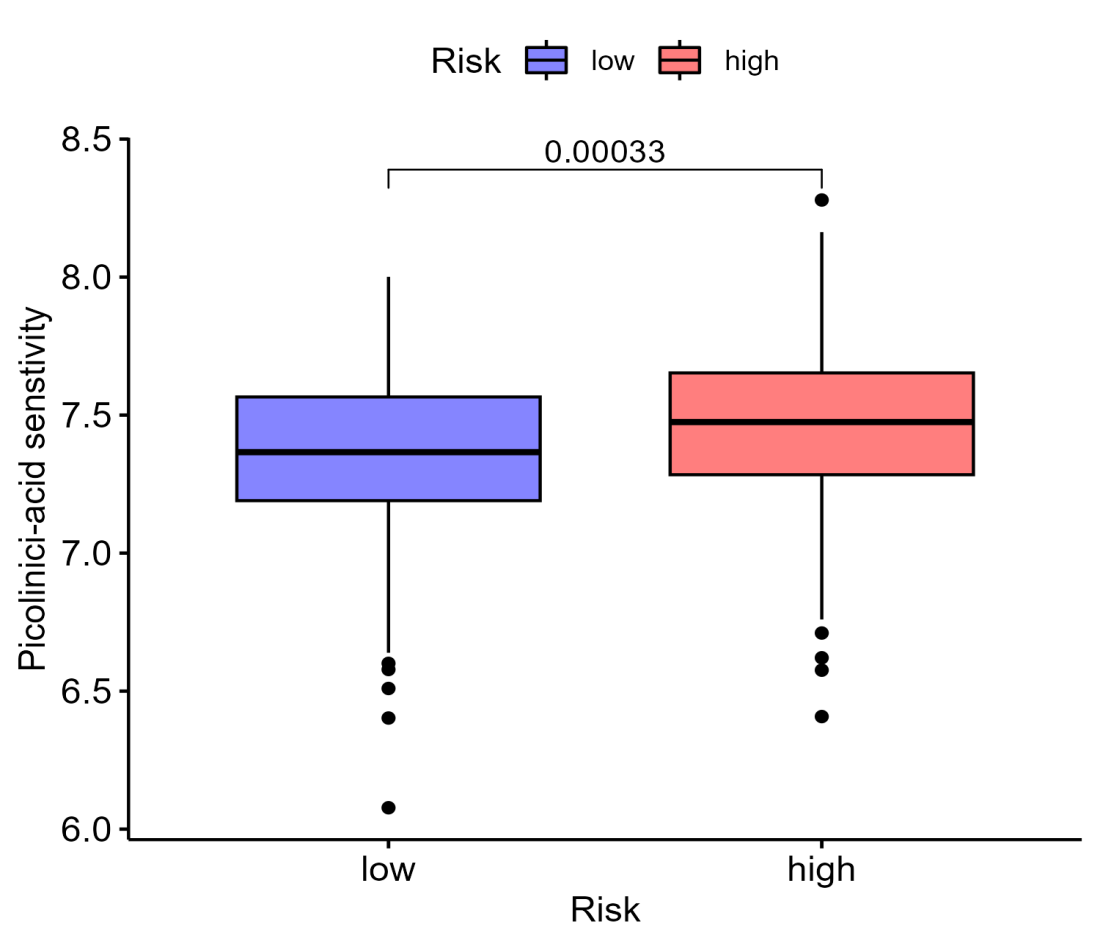


**Figure S41** Significant IC50 difference from Picolinici-acid between high- and low-risk subgroups


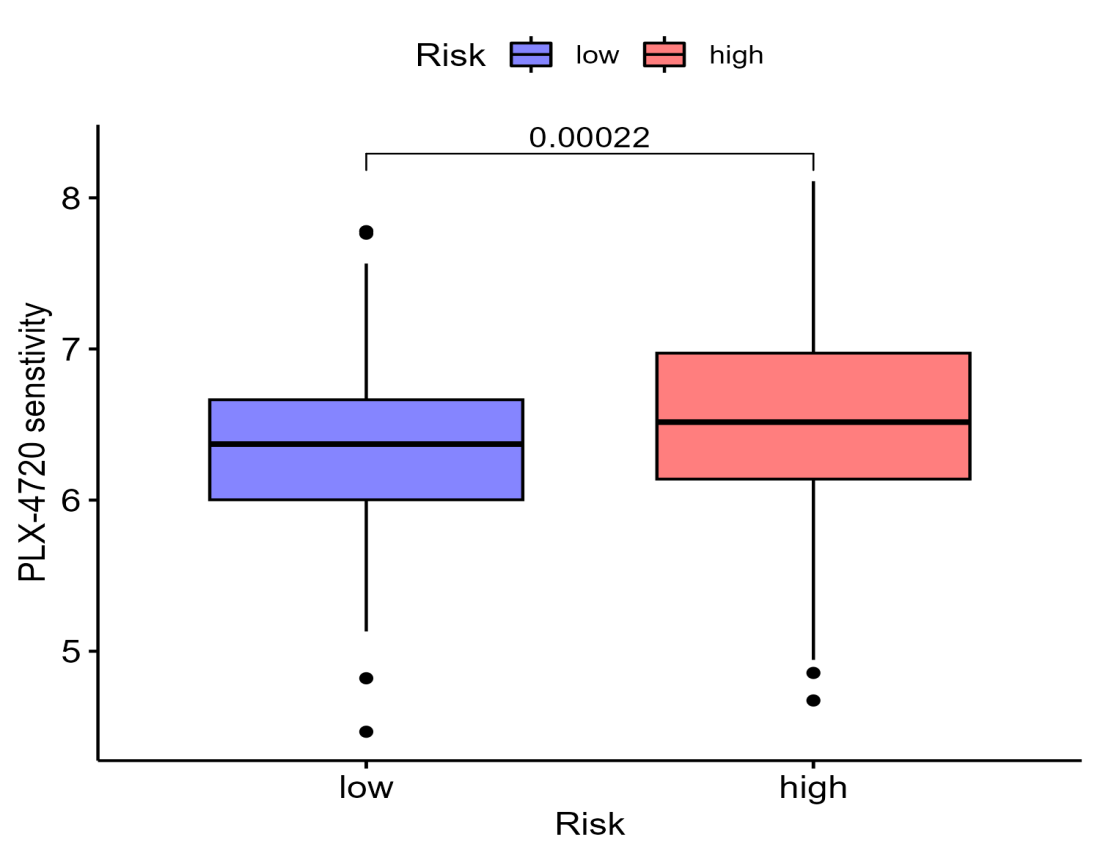


**Figure S42** Significant IC50 difference from PLX-4720 between high- and low-risk subgroups


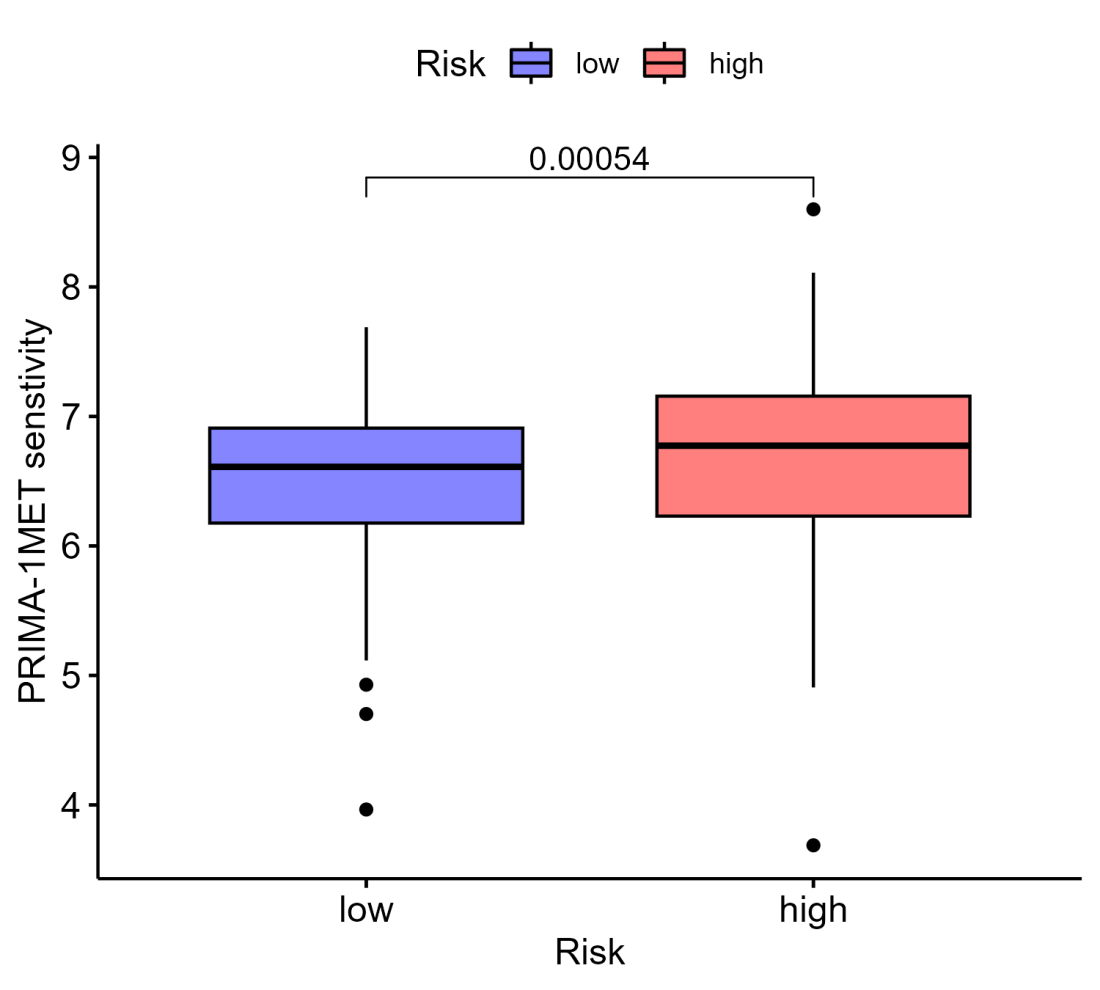


**Figure S43** Significant IC50 difference from PRIMA-1MET between high- and low-risk subgroups


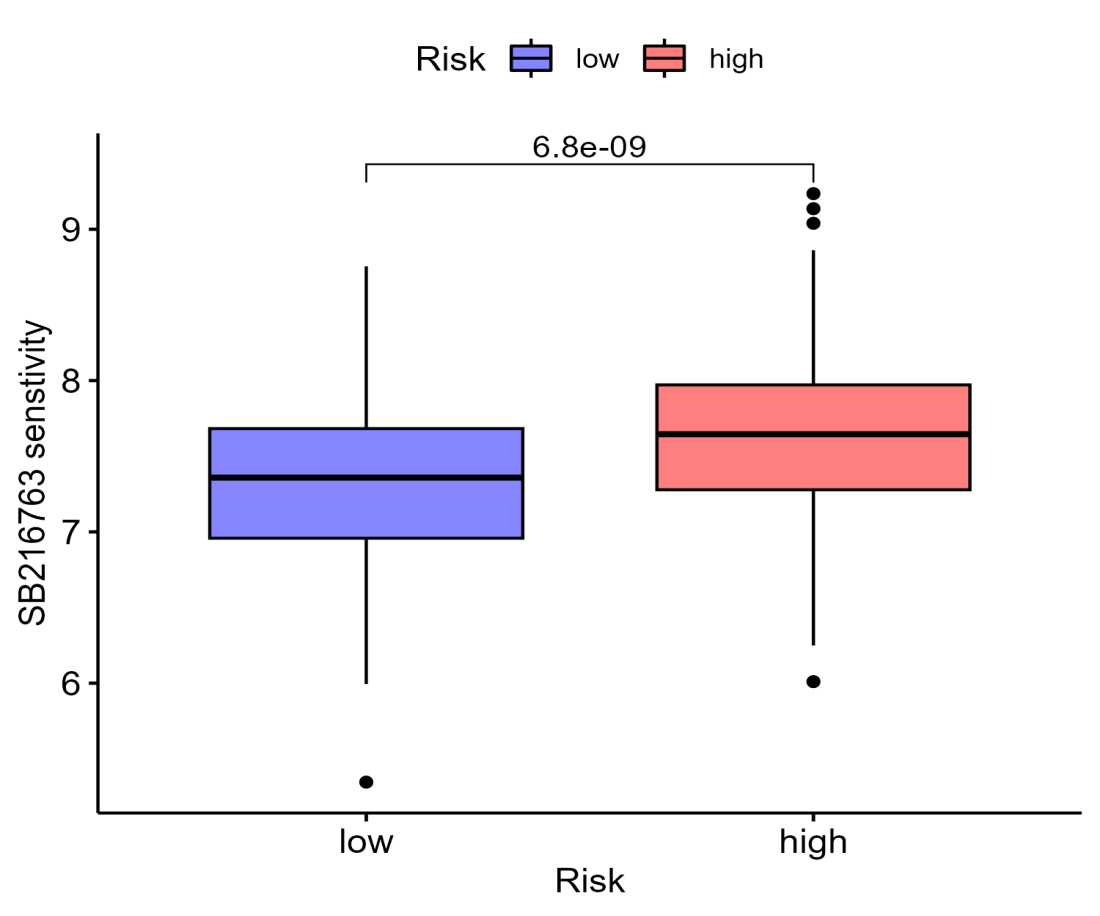


**Figure S44** Significant IC50 difference from SB216763 between high- and low-risk subgroups


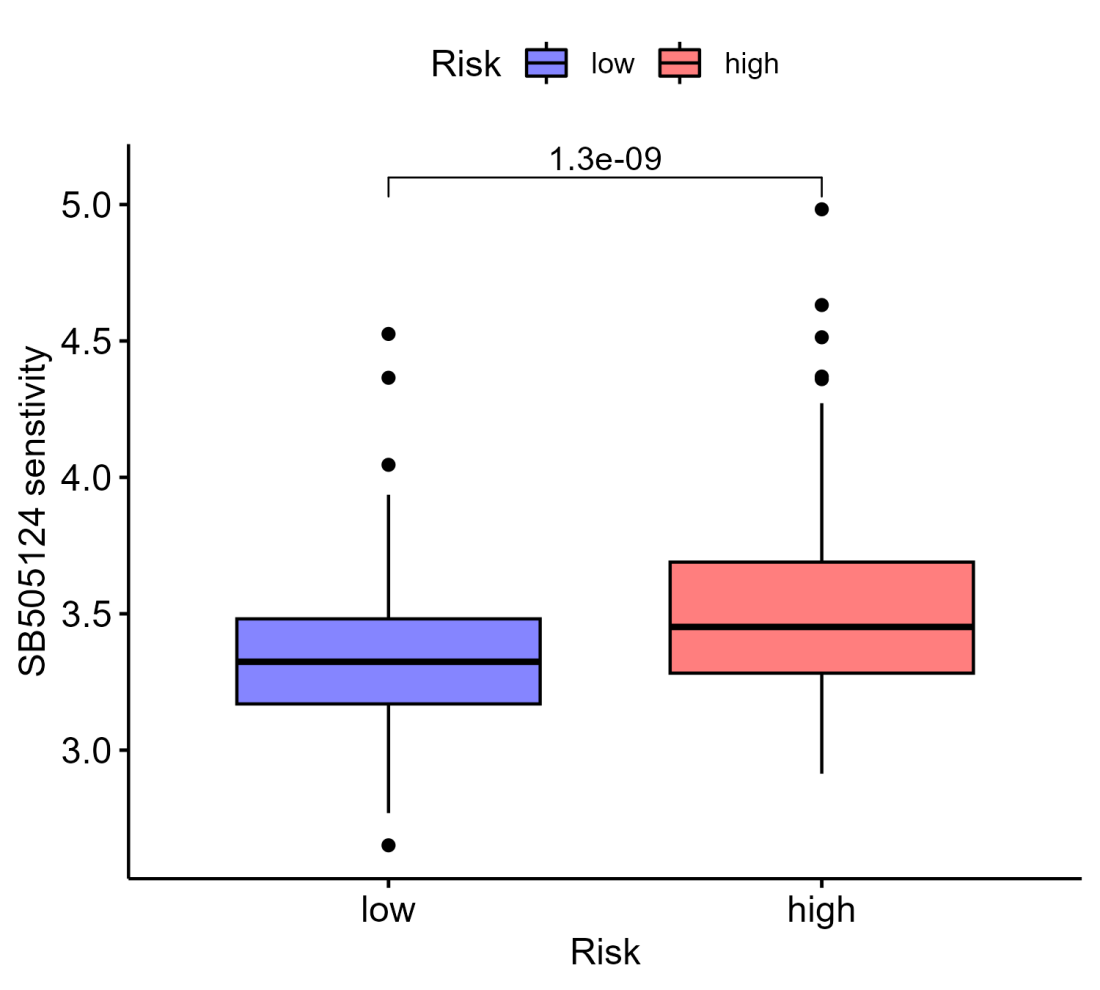


**Figure S45** Significant IC50 difference from SB505124 between high- and low-risk subgroups


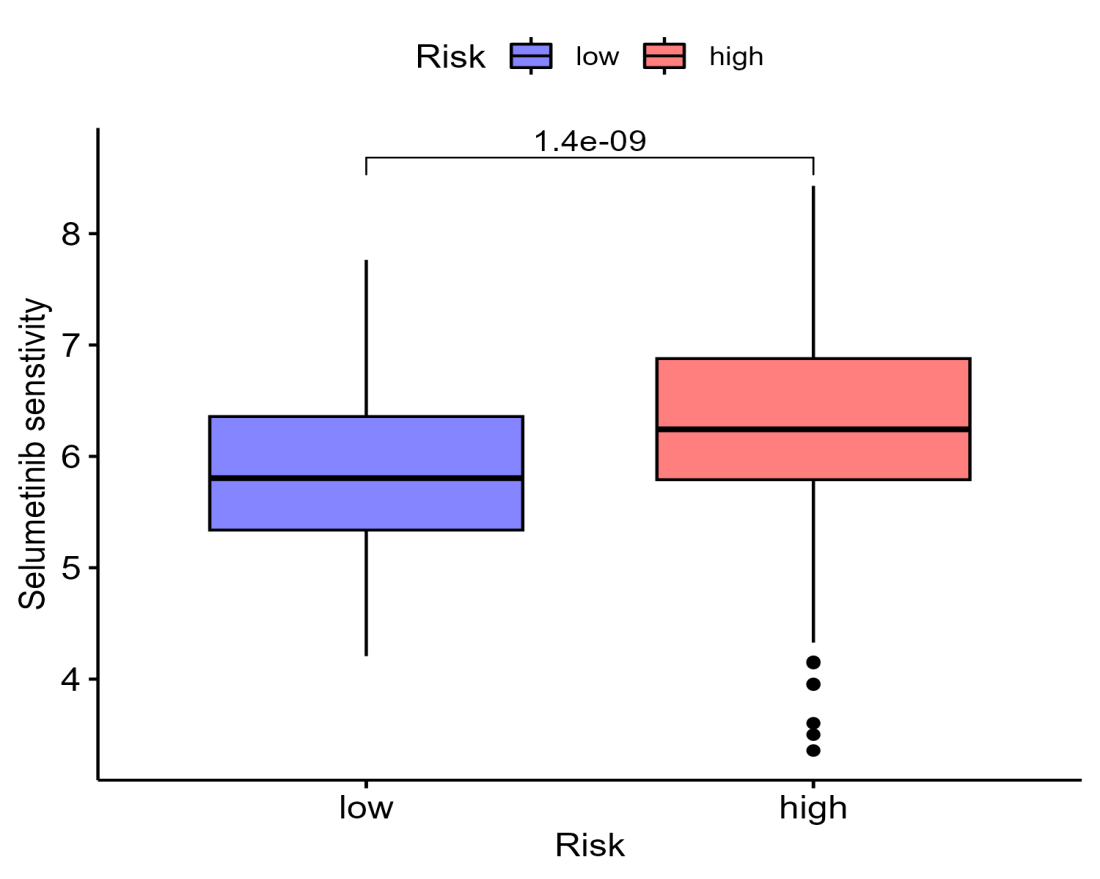


**Figure S46** Significant IC50 difference from Selumetinib between high- and low-risk subgroups


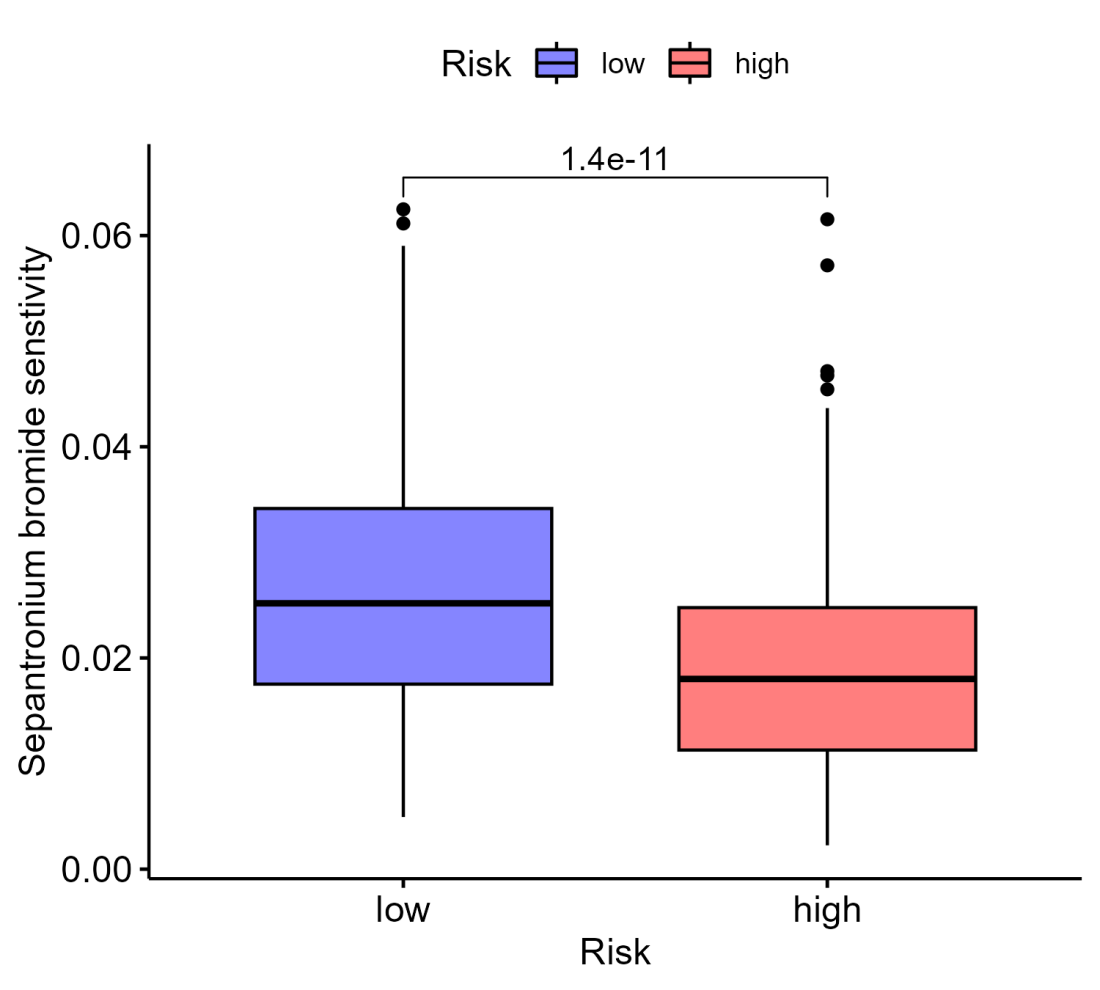


**Figure S47** Significant IC50 difference from Sepantronium bromide between high- and low-risk subgroups


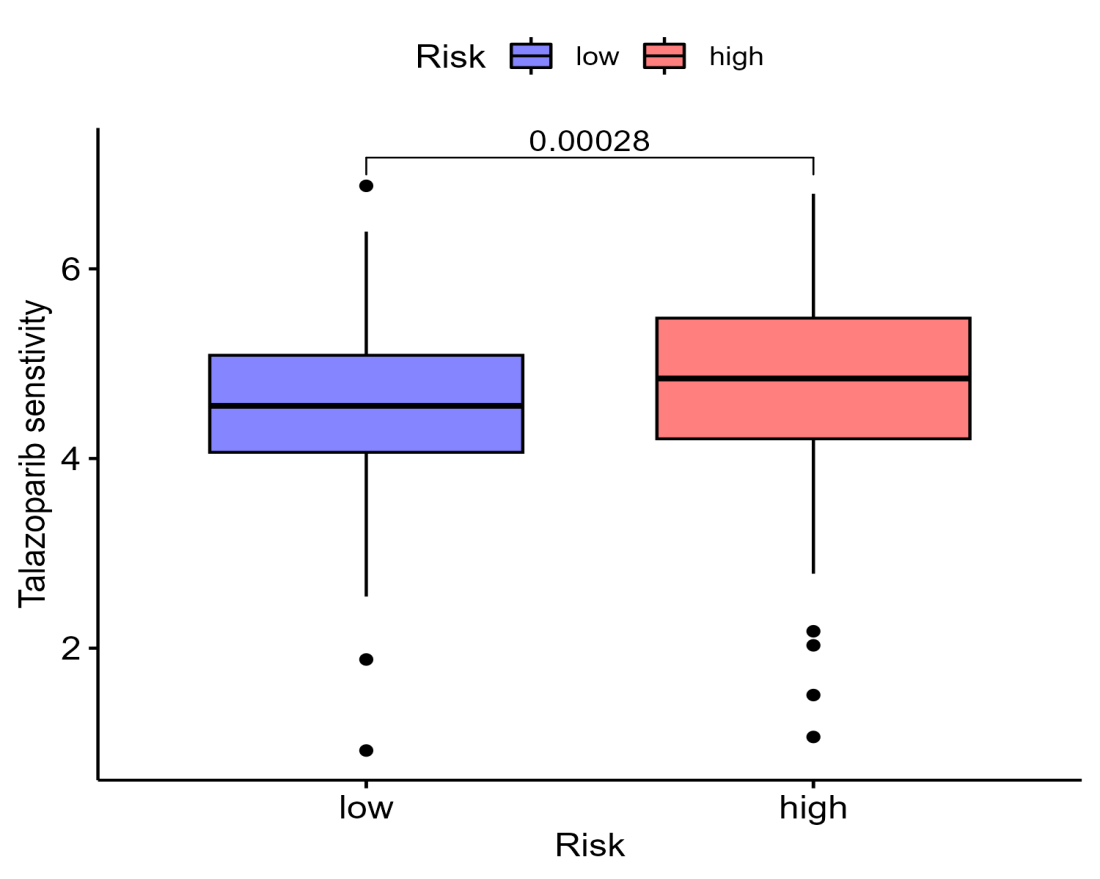


**Figure S48** Significant IC50 difference from Talazoparib between high- and low-risk subgroups


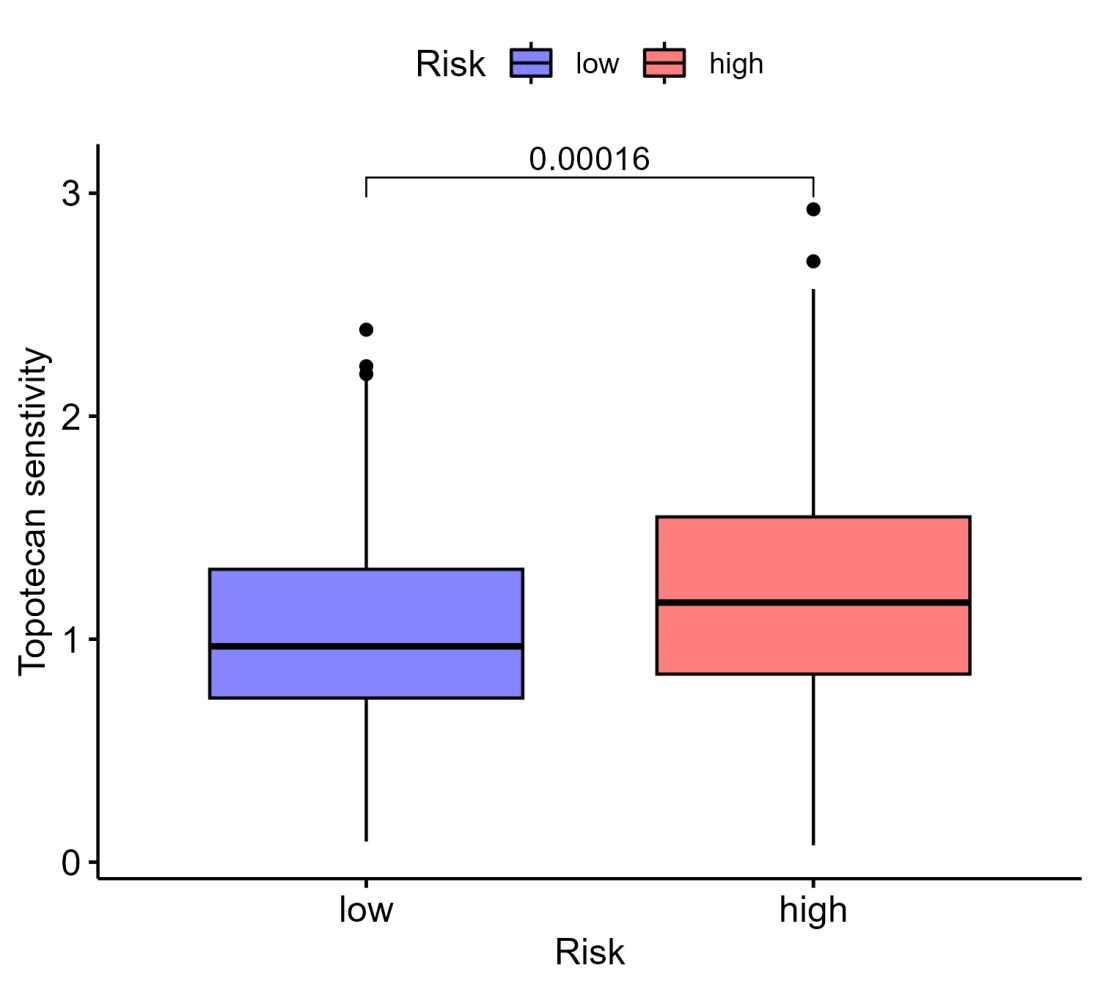


**Figure S49** Significant IC50 difference from Topotecan between high- and low-risk subgroups


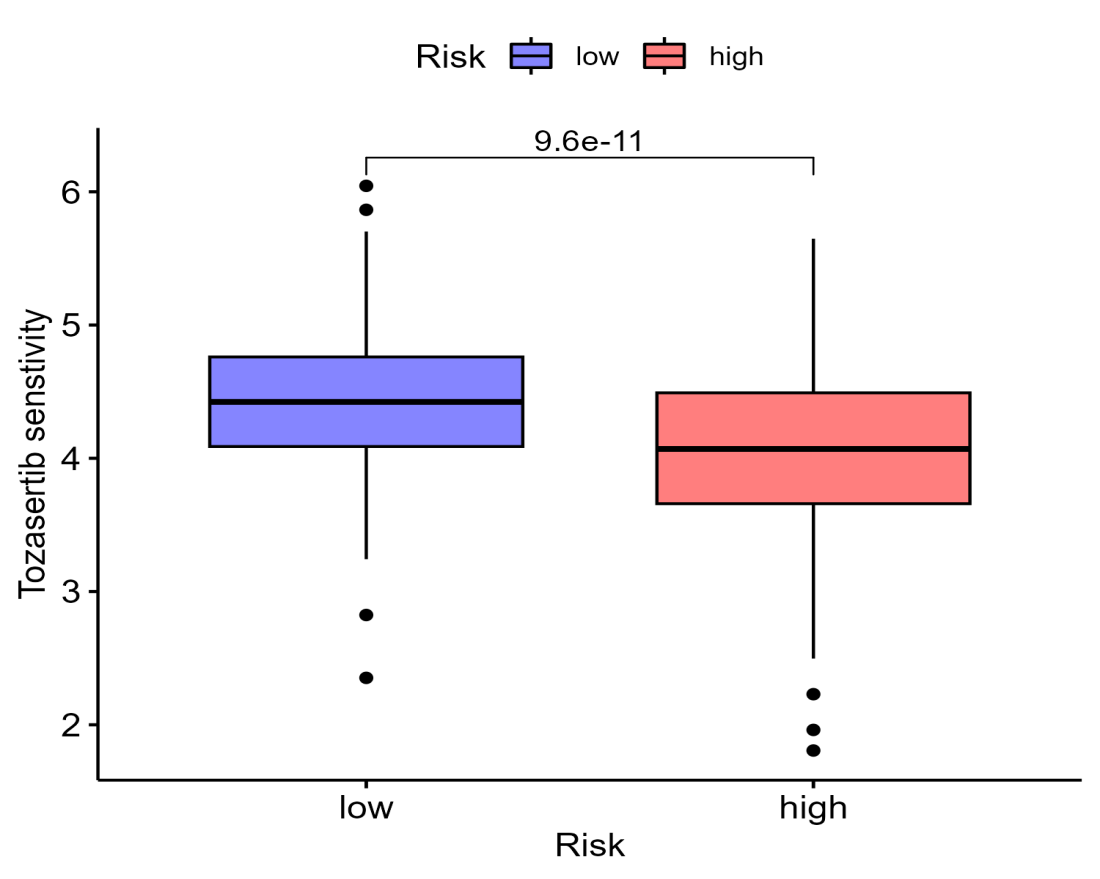


**Figure S50** Significant IC50 difference from Tozasertib between high- and low-risk subgroups


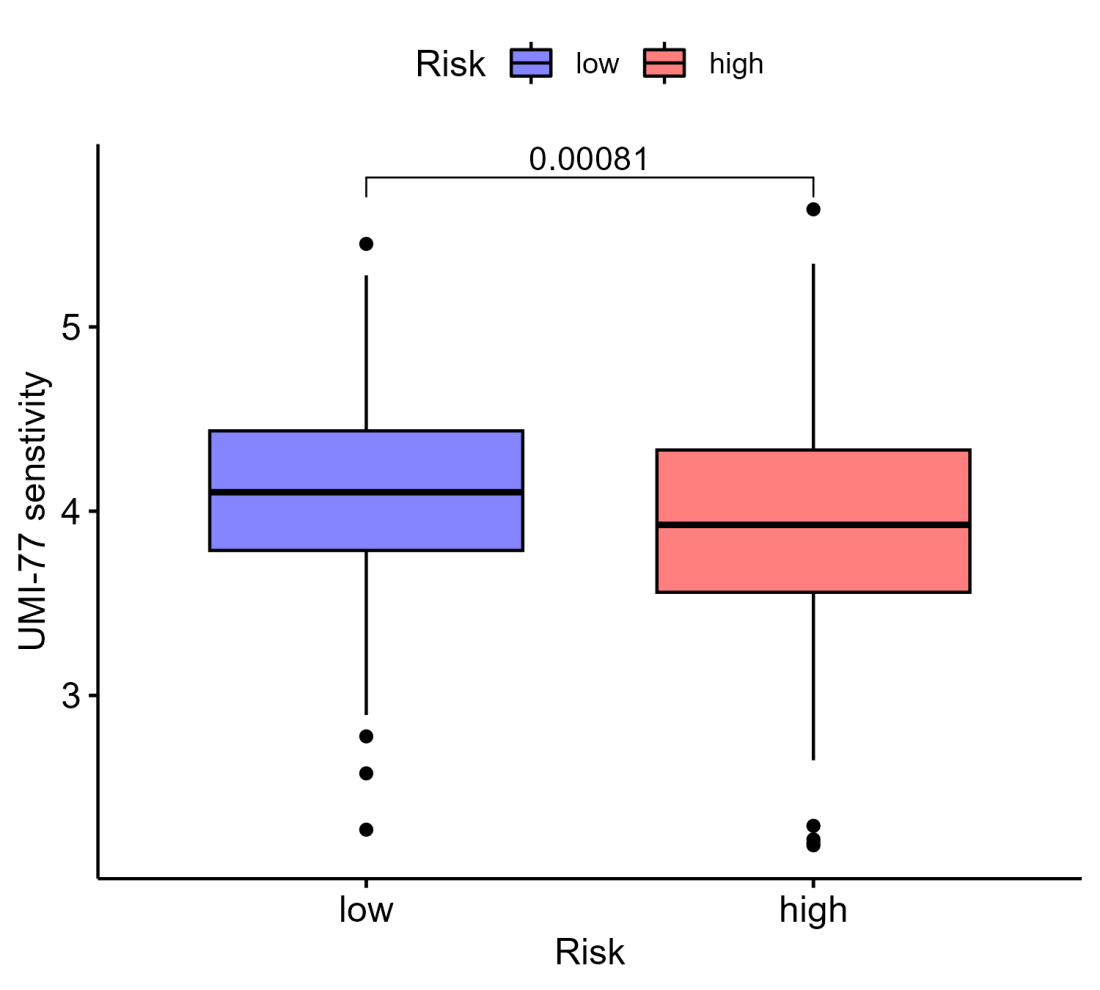


**Figure S51** Significant IC50 difference from UMI-77 between high- and low-risk subgroups


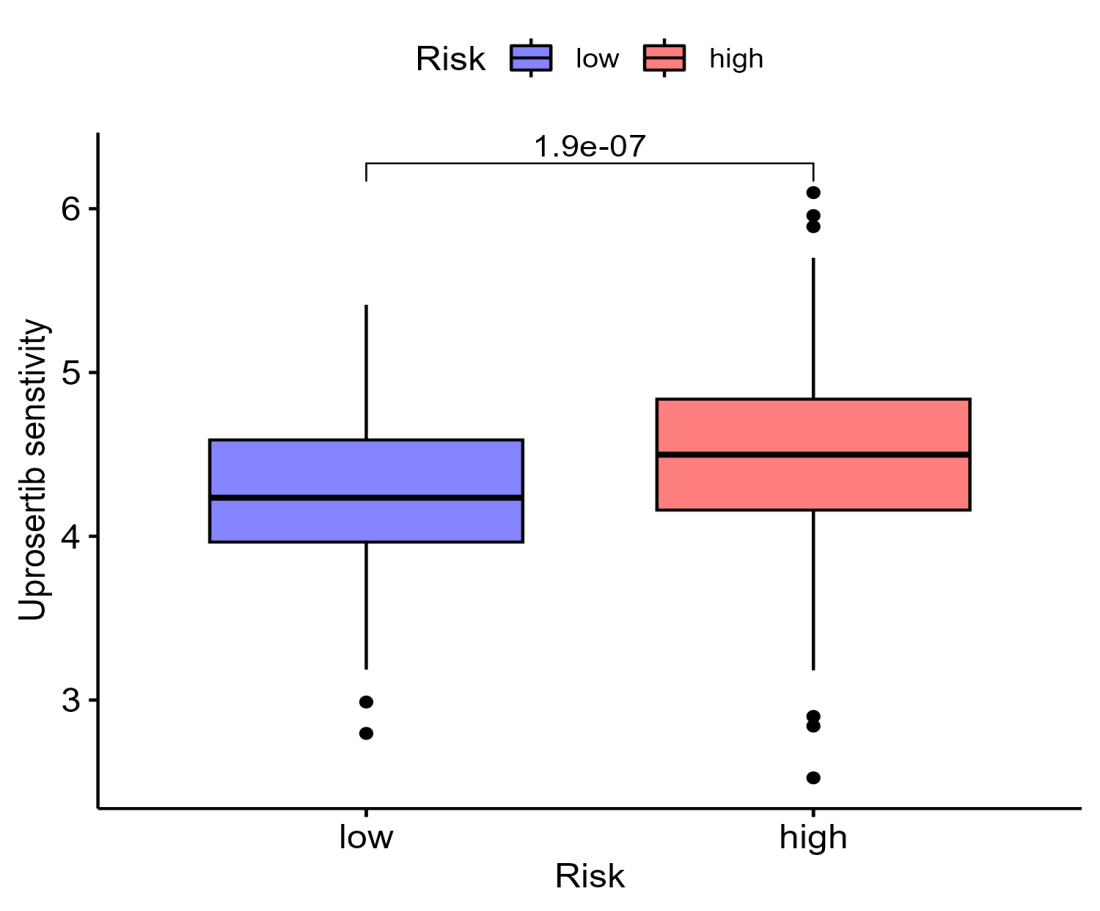


**Figure S52** Significant IC50 difference from Uprosertib between high- and low-risk subgroups


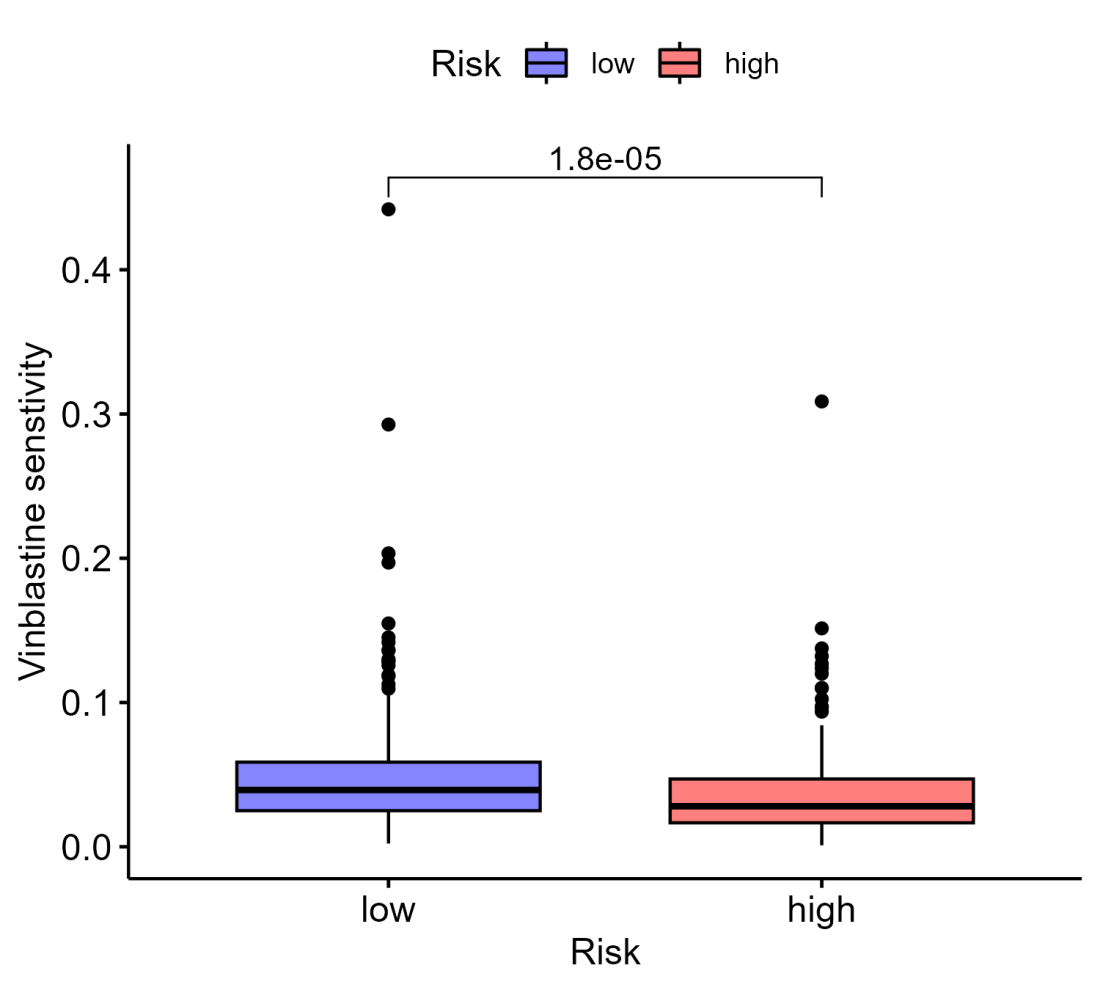


**Figure S53** Significant IC50 difference from Vinblastine between high- and low-risk subgroups


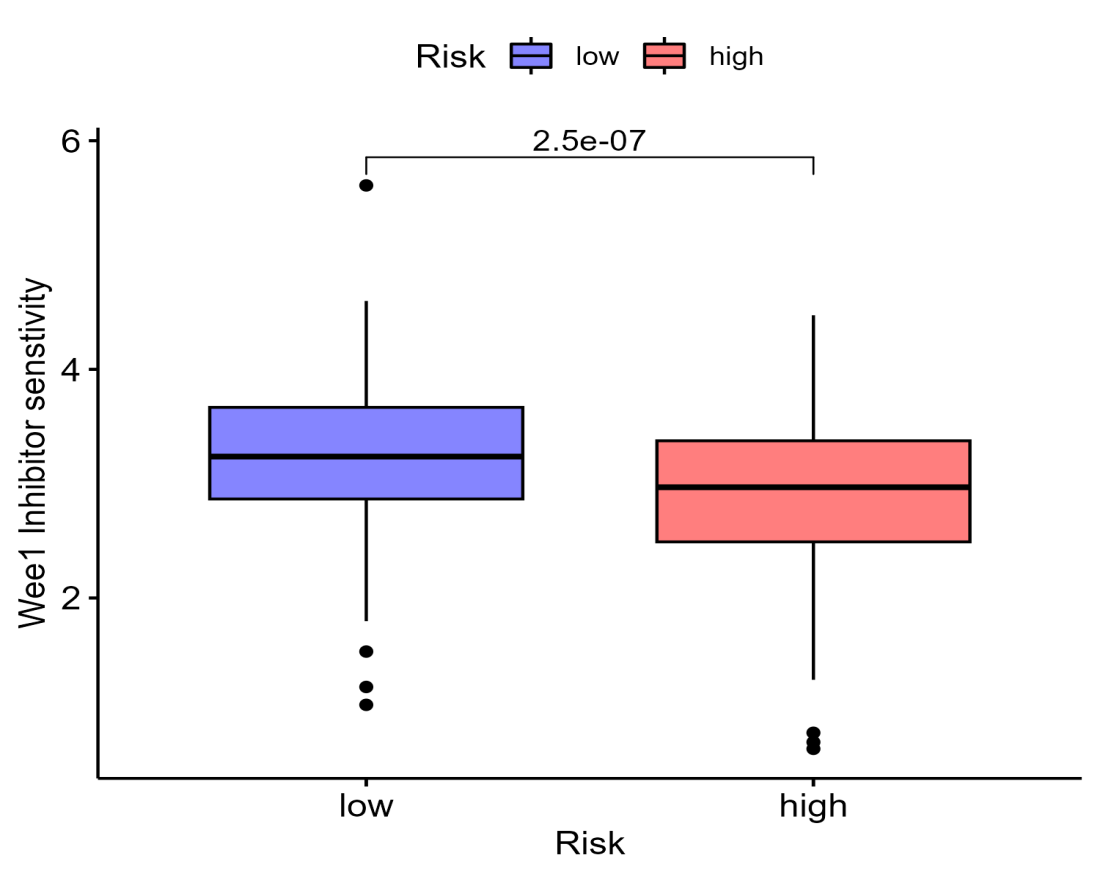


**Figure S54** Significant IC50 difference from Wee1 Inhibitor between high- and low-risk subgroups


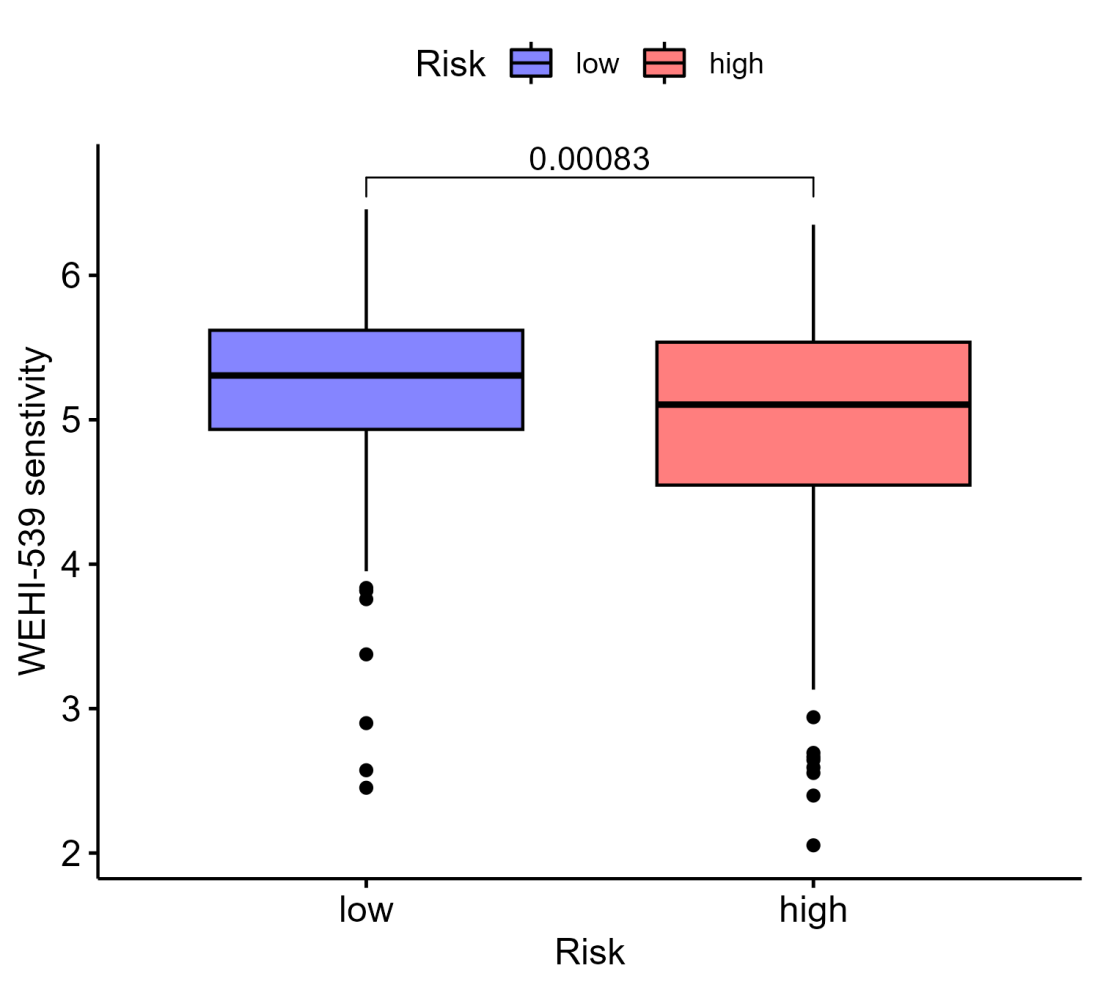


**Figure S55** Significant IC50 difference from WEHI-539 between high- and low-risk subgroups


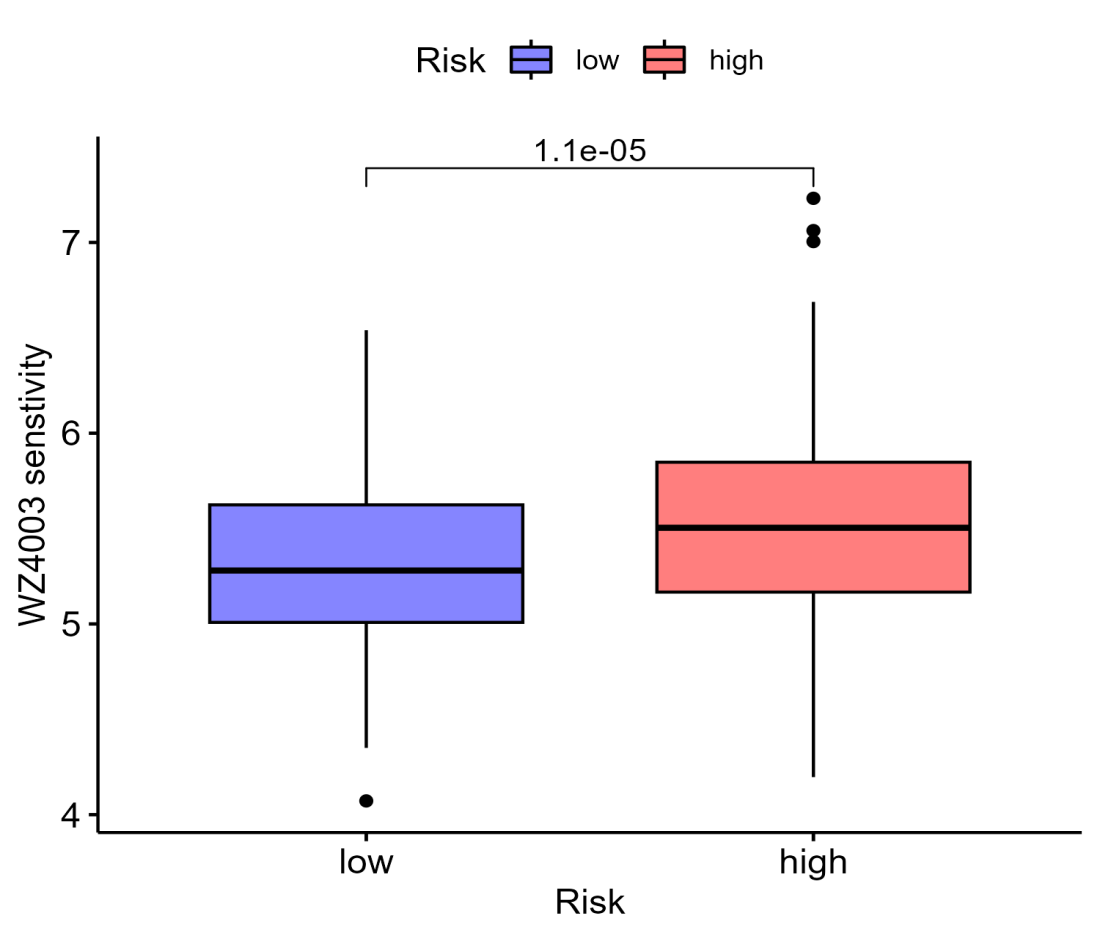


**Figure S56** Significant IC50 difference from WZ4003 between high- and low-risk subgroups


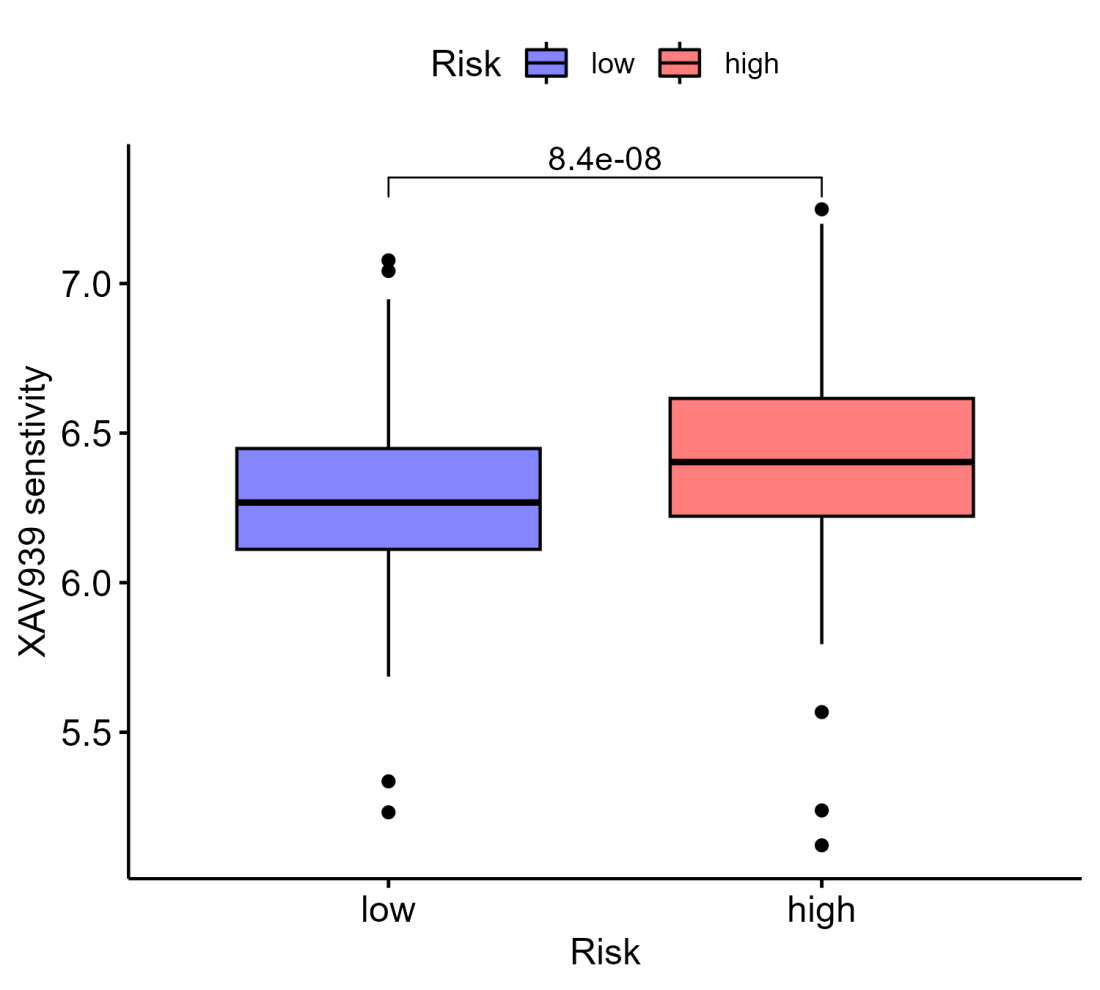


**Figure S57** Significant IC50 difference from XAV939 between high- and low-risk subgroups


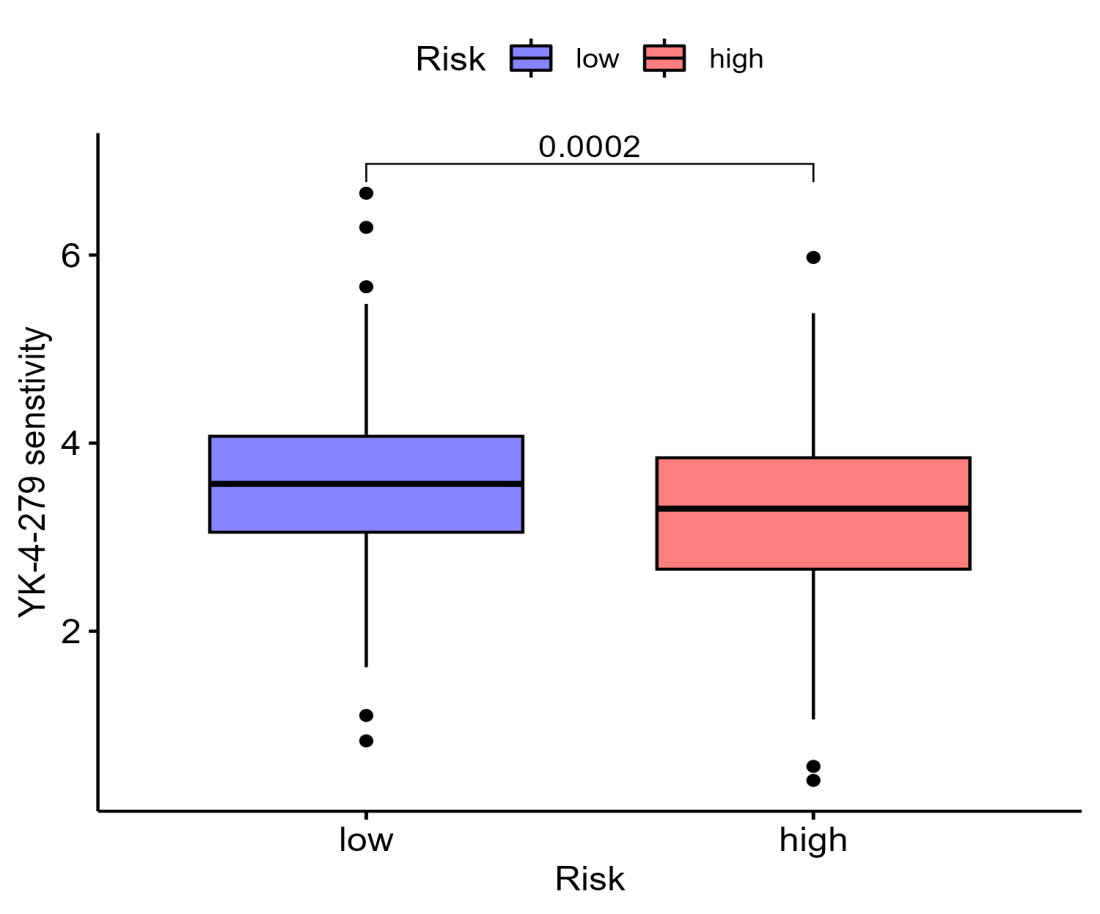


**Figure S58** Significant IC50 difference from YK-4-279 between high- and low-risk subgroups


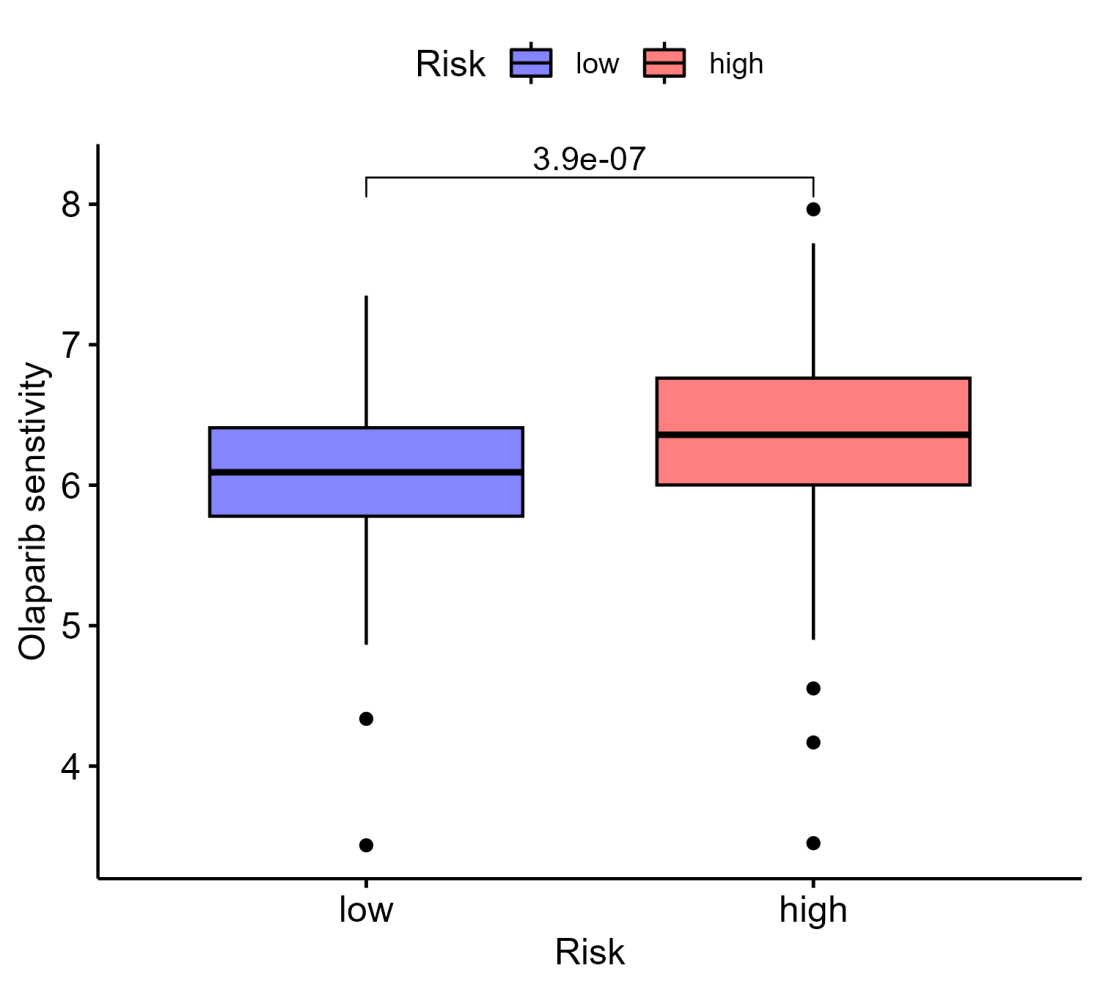


**Figure S59** Significant IC50 difference from Olaparib between high- and low-risk subgroups
